# Efficacy and safety of mesenchymal stem cells co-infusion in allogeneic hematopoietic stem cell transplantation: a systematic review and meta-analysis

**DOI:** 10.1186/s13287-021-02304-x

**Published:** 2021-04-20

**Authors:** Teng Li, Chengxin Luo, Jiasi Zhang, Ling Wei, Wei Sun, Qin Xie, Yan Liu, Yongli Zhao, Shuangnian Xu, Lihua Wang

**Affiliations:** 1 Center for Hematology, Southwest Hospital, Army Medical University (Third Military Medical University), #30 Gaotanyan Street, Shapingba District, Chongqing, 400038 People’s Republic of China; 2 Admin Office of Southwest Hospital, Army Medical University (Third Military Medical University), #30 Gaotanyan Street, Shapingba District, Chongqing, 400038 People’s Republic of China; 3 Teaching-Research Office of Nursing, Southwest Hospital, Army Medical University (Third Military Medical University), Chongqing, People’s Republic of China; 4 School of Nursing, Army Medical University (Third Military Medical University), Chongqing, People’s Republic of China

**Keywords:** Hematopoietic stem cell transplantation, Mesenchymal stem cells, Engraftment, Graft-versus-host disease

## Abstract

**Background:**

Allogeneic hematopoietic stem cell transplantation (allo-HSCT) is life-saving for severe hematological conditions. However, its outcomes need further improvement, and co-infusion of mesenchymal stem cells (MSCs) may show promise. A growing body of research on this subject exists, while the results of different trials are conflicting. A systematic review and meta-analysis is needed to appraise the real efficacy and safety of MSC co-transplantation in allo-HSCT.

**Methods:**

Studies comparing MSC co-transplantation in allo-HSCT with allo-HSCT alone were searched in six medical databases from inception to June 10, 2020. The primary outcomes were engraftment and graft-versus-host disease (aGVHD and cGVHD, respectively). Other outcomes included overall survival (OS), relapse rate (RR), non-relapse mortality (NRM), and immune reconstitution. Information was independently extracted by two investigators. Methodological quality was assessed using the Cochrane Collaboration tool. Meta-analysis was performed using RevMan 5.4.

**Results:**

Six randomized controlled trials (RCTs) and 13 non-randomized controlled trials (nRCTs) were included. MSC co-infusion resulted in shorter times to neutrophil engraftment (RCTs: standardized mean difference (SMD) − 1.20, *p* = 0.04; nRCTs: SMD − 0.54, *p* = 0.04) and platelet engraftment (RCTs: SMD − 0.60, *p* = 0.04; nRCTs: SMD − 0.70, *p* = 0.01), a lower risk of cGVHD (RCTs: risk ratio (RR) 0.53, *p* = 0.01; nRCTs: RR 0.50, *p* <  0.01), and a slightly positive trend towards reducing the risk of aGVHD and NRM, without affecting RR and OS. Subgroup analyses revealed that when MSCs were co-transplanted, children and adolescents, and patients receiving human leukocyte antigen (HLA)-nonidentical HSCT showed improvements in engraftment and incidence of GVHD and NRM; adults and patients who received HLA-identical HSCT had lower cGVHD; patients with malignancies exhibited improvements in GVHD and NRM incidence; and patients with non-malignancies experienced accelerated engraftment. Notably, a reduced OS was observed in patients with hematological malignancies undergoing HLA-identical HSCT.

**Conclusion:**

MSC co-infusion generally improved engraftment and reduced cGVHD, without increasing mortality or relapse. Regarding aGVHD and NRM, the effects of MSCs were not quite significant. Specifically, our data support the utilization of MSC co-transplantation in children and young individuals with HLA-nonidentical HSCT, but not in adult patients with hematological malignancies undergoing HLA-identical HSCT.

## Background

Throughout the past few decades, allogeneic hematopoietic stem cell transplantation (allo-HSCT) has been a life-saving strategy for many malignant or non-malignant hematological disorders, providing an opportunity for the complete recovery of blood cellular constituents and graft-versus-leukemia (GVL) effects [1]. Classically, prior to the infusion of hematopoietic stem cells (HSCs), recipients must receive a conditioning regiment (irradiation-based or chemotherapy-based) according to the patient’s physical condition, disease type, stage, and donor options, to eliminate malignant hematologic cells and suppress the immune system [1]. Successful engraftment of donor stem cells is the precondition for the therapeutic effect of HSCs. However, several severe complications can restrict the success of transplantation. For example, graft-versus-host disease (GVHD), either in acute or chronic form, is a specific and potentially fatal complication of allo-HSCT [2], as along with disease relapse and opportunistic bacterial, viral, or fungal infections. All of these factors can cause significant morbidity and mortality in allo-HSCT recipients.

Mesenchymal stem cells (MSCs) are a population of multipotent, non-hematopoietic stem cells with the capability to differentiate into various cell types, such as adipocytes, osteocytes, chondrocytes, and cells present in other connective tissues [3, 4]. MSCs, which are present in adult and fetal tissues, could be cultured in vitro and transplanted [3, 5]. The cells are mainly obtained from bone marrow (BM), adipose tissue, peripheral blood (PB), umbilical cord blood (UCB), and amniotic fluid and amnion [3, 5, 6]. Characterized by plasticity, self-renewal, immunomodulation, and anti-inflammatory properties, MSCs are potentially considered for regeneration medicine, transplantation, and cell therapy for many diseases [3, 5, 7]. Unlike HSCs, MSCs can avoid host immune responses because they do not express human leukocyte antigen (HLA) class-II and many costimulatory molecules, indicating that allogeneic MSCs could be infused without being rejected [5, 8]. MSCs also modulate immune responses by secreting various mediators and participating in complex interactions with dendritic cells (DCs) and B and T cells, including T regulatory cells, natural killer cells (NK cells), and a variety of T helper cells [9]. For example, MSCs act on CD4+ T cells via interferon-γ and transforming growth factor-β [5]. MSCs suppress monocyte differentiation into DCs and indirectly moderate the eventual specific immune response [8]. In addition, MSCs increase the expression of various hematopoietic factors, providing stromal support for the survival and proliferation of HSCs [3, 5]. Furthermore, MSCs exert antitumorigenic effects by homing to tumors, inhibiting the vasculature and inducing cell cycle arrest [3]. Therefore, we hypothesized that these characteristics of MSCs would effectively improve the outcomes of allo-HSCT through the induction of mixed chimerism, treatment or prophylaxis of GVHD, acceleration of hematopoietic cell engraftment, and induction of the GVL effect [2, 5]. Nonetheless, MSCs not only suppress tumor growth but may also promote tumor growth [3, 10], and the dual roles of MSCs in tumor cell proliferation and apoptosis remain controversial [11]. Herein, a thorough understanding of the clinical safety and efficacy of MSCs in the treatment of hematological abnormalities and blood malignancies is needed.

Various clinical trials [12–17] have been carried out to investigate the safety and efficacy of MSCs co-infused in allo-HSCT recipients, but controversy persists, probably due to heterogeneous doses and sources of MSCs, diverse patient characteristics, and HSCT types. To date, a few literature reviews [18, 19] have summarized these conflicting results but have not yielded any encouraging findings. Focusing on the time point at which MSCs are co-infused during allo-HSCT, we updated the previous work [18] by adding several current original clinical studies and articles that were published in Chinese. Moreover, we performed subgroup analyses based on the aforementioned factors to identify the pros and cons of MSCs co-transplanted with allo-HSCs in a particular clinical situation.

## Methods

This systematic review was performed according to the Preferred Reporting Items for Systematic Reviews and Meta-Analysis (PRISMA) guidelines [20]. Ethical approval was not needed, as it was a systematic review of published summary data [21].

### Review objective

The specific research question was the following: Are the outcomes of MSC co-transplantation with allo-HSCs more effective than allo-HSCT alone in people with hematological diseases? The eligibility criteria were defined and listed in Additional file 1.

### Data sources and searches

We (Teng Li and Shuangnian Xu) systematically searched the literature in PubMed, Embase, Web of Science, SinoMed, Cochrane Library, and http://ClinicalTrials.gov from the date of record to June 10, 2020. The language of publications was restricted to Chinese and English. The detailed search strategies are presented in Additional file 2.

### Study selection

**Fig. 1:**
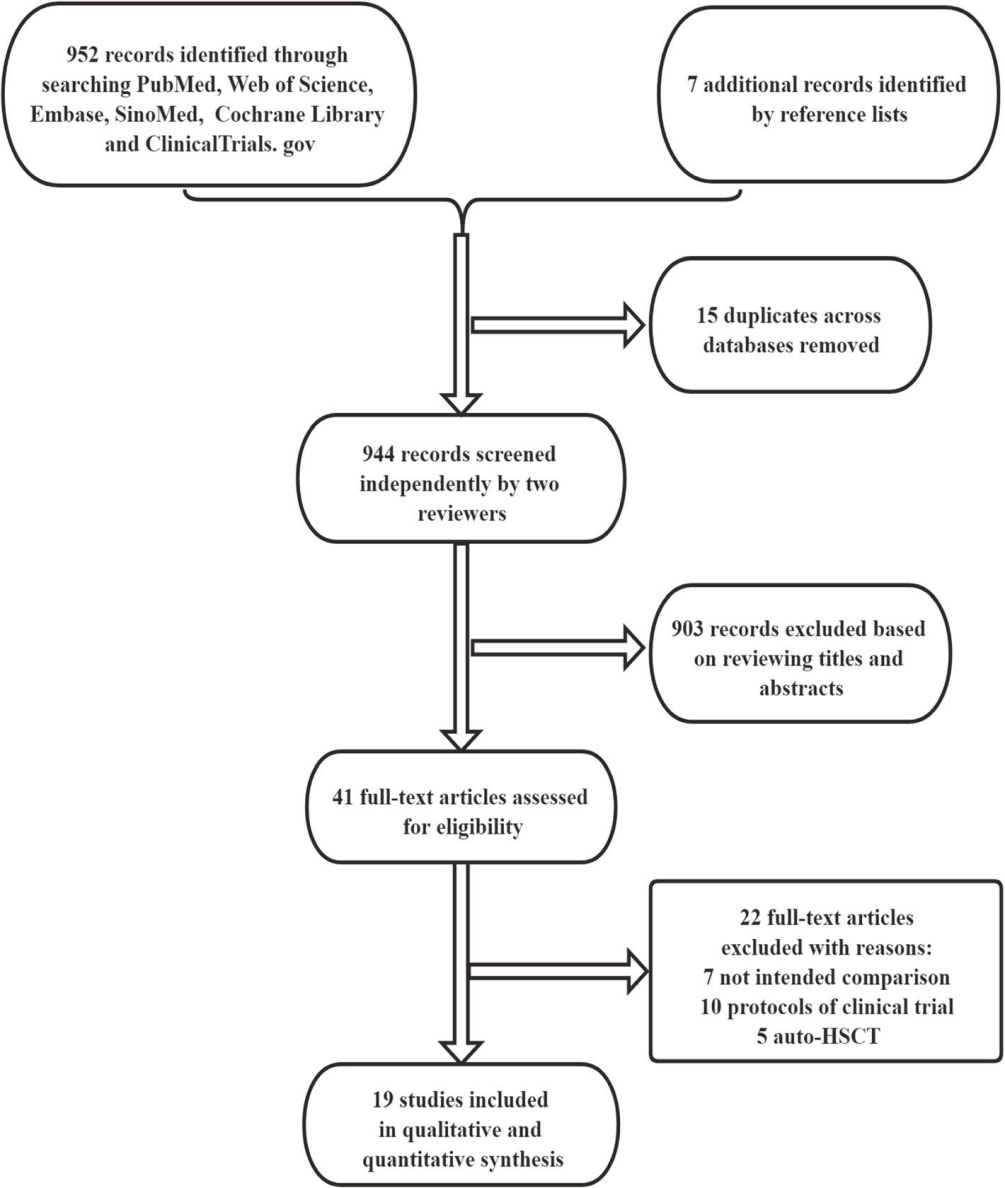
Flow diagram of study selection

The titles and abstracts of articles identified using the search strategies were independently screened by 2 reviewers (Teng Li and Chengxin Luo). Duplicates were eliminated electronically. All potentially relevant publications were retrieved in full, and then the eligibility criteria were applied to the full texts of these studies. Finally, through a discussion or resorting to other team members (Shuangnian Xu and Lihua Wang), the two reviewers reached a consensus on the papers that should be included. The flow diagram of the literature search and selection process is shown in Fig. 1.

### Definition of outcomes

We chose engraftment and the incidence of GVHD as primary outcomes and relapse rate (RR), overall survival (OS), treatment-related mortality or non-relapse mortality (TRM/NRM), and immune reconstitution as secondary outcomes. (I) Engraftment: neutrophil engraftment was defined as the time point of the first 3 consecutive days after HSCT when the patient reached an absolute neutrophil count (ANC) of ≥ 0.5 × 10^9^ cells/L [18, 22]; platelet engraftment was defined as the first day that the patient did not have a platelet transfusion and had a platelet count (PLT) of ≥ 20 × 10^9^ cells/L for 7 consecutive days [22]. (II) GVHD: The incidence and severity of GVHD were determined adhering to a National Institutes of Health consensus conference [23]. (III) RR: The relapse rate was defined as the recurrence of disease [18]. (IV) OS: Overall survival was defined as the time from transplantation until death from any cause [18]. (V) TRM/NRM: Death in the absence of persistent relapse was categorized as non-relapse mortality or treatment-related mortality [24]. (VI) Immune reconstitution: The recovery of NK cells, monocytes, DCs, B cells, T cells, and immunoglobulin levels [25, 26].

### Data extraction

Basic study information, patient characteristics, sources of HSCs and MSCs, donor types for HSCs and MSCs, HSC HLA matching, MSC dose, median follow-up time and major clinical outcomes (engraftment, GVHD, RR, OS, NRM/TRM, and immune reconstitution) were independently extracted from each included study by 2 investigators (Teng Li and Chengxin Luo) with specially designed forms. We resolved discrepancies by discussion or resorting to other team members (Shuangnian Xu and Lihua Wang). The primary characteristics of the included studies are presented in Table 1. All the outcomes extracted from each study are shown in Tables 2, 3, and 4.

**Table 1:** Characteristics of the included studies in systematic review (*N* = 19)

First author, year, and country	Study	Disease	No. (MSCs+/MSCs−)	Median/mean age (year) (MSCs+/MSCs−)	HSCs source (donor type)	HSCs HLA matching	MSCs source (donor type)	MSCs dose (10^6^/kg)	Median follow-up time (month) (MSCs+/MSCs−)
Ning, 2008, China [24]	RCT	HM	25 (10/15)	38/37	BM, PB, BM+PB (RD)	ID	BM (RD)	0.34 (0.03–1.53)	36.6 (0.6–44.0)
Ghavamzad, 2010, Iran [27]	RCT	NMD	48 (25/23)	17/16	PB, BM (RD)	ID	NA (RD)	1.45–1.80	10 (1–28)
Liu, 2011, China [22]	RCT	HM	55 (27/28)	30/31.5	BM+PB (RD)	HID	BM (RD, TPD)	0.3–0.5	23.7 (0.7–33.5)
Wu, 2013b, China [28]	RCT	HM	20 (8/12)	9.8/8.5	UCB (URD)	MM	UCB (URD)	7.19 (2.44–10.12)	16.5 (11–27)/18.5 (12–31)
Mareika, 2016, Belarus [29]	RCT	HM	22 (10/12)	13 (5–24)	NA (NA)	NA	BM (NA)	1.56 ± 0.4	38 (5.7–59.4)
Xiang, 2017, China [13]	RCT	HM	64 (32/32)	5.5 ± 1.4/5.2 ± 1.2	PB (URD)	ID	UCB (URD)	1.0	24
Ball, 2007, Netherlands [16]	HCT	HM and NMD	61 (14/47)	8 (1–16)/7.1 (1–17)	PB (RD)	HID	BM (RD)	1.6 (1.0–3.3)	(3–28)/(32–110)
Gonzalo-Daganzo, 2009, Spain [30]	PCT	HM	55 (9/46)	32/35	UCB (URD)	MM	BM (URD)	1.2 (1.04–2.22)	7.4 (1–22)/24 (1–107)
MacMillan, 2009, USA [31]	HCT	HM	30 (7/23)	7.5 (0.2–16)	UCB (URD)	MM	BM (RD)	2.1 (0.9–5.0)	81.6
Baron, 2010, Belgium [32]	HCT	HM	36 (20/16)	58/55	PB (URD)	MM	BM (URD)	NA	18.7 (13.3–30.3)
Hou, 2010 China [33]	PCT	HM	35 (15/20)	32 (14–45)/28.5 (12–48)	NA (RD)	HID	BM (RD, 2; TPD, 13)	0.41 (0.22–0.52)	6
Bernardo, 2011, Italy [15]	HCT	HM	52 (13/39)	2/4	UCB (URD)	MM	BM (RD)	1.9 (1–3.9)	36 (28/42)
Lee, 2013, Korea [14]	HCT	HM	16 (7/9)	6.9/9.5	UCB (URD)	MM	UCB (URD)	1.0 in 4 pts, 5.0 in 3 pts	24
Wu, 2013a, China [34]	PCT	HM and NMD	14 (5/9)	8.8/7.8	UCB (URD)	MM	UCB (URD)	5.76 (3.12–8.21)	27 (24–31)
Xiao, 2013, China [35]	PCT	HM and NMD	15 (7/8)	30 (12–60)/35.5 (16–54)	PB (URD)	NA	UCB (URD)	NA	15
Wang, 2015, China [36]	PCT	NMD	14 (7/7)	28 (22–43)	PB (RD)	MM	UCB (URD)	Total, 30.0	14.5 (6–74)
Zhang, 2015, China [37]	HCT	HM	49 (22/27)	22.5 (3–48)/23 (3–43)	PB (URD)	MM	UCB (URD)	1.0	22 (1–98)
Kang, 2017, China [38]	HCT	HM	47 (34/13)	7 (1.5–13)	PB, BM (RD)	HID	UCB (URD)	1.0	20 (0.5–67)
Ghavamzad, 2017, Iran [39]	PCT	NMD	70 (41/29)	NA	NA (RD)	ID	NA (URD)	1.0–2.0	35.76/31.44

*RCT* randomized controlled trial, *PCT* prospective controlled trial, *HCT* historical controlled trial, *HM* hematological malignancies, *NMD* non-malignant disorders, *PB* peripheral blood, *BM* bone marrow, *UCB* umbilical cord blood, *RD* related donor, *URD* unrelated donor, *NA* not available, *pts* patients, *ID* identical, *HID* haploidentical, *MM* mismatched

**Table 2:** Outcomes (engraftment and GVHD) of included studies

First author, year, and country	ANC ≥ 0.5 × 10^9^/L (days)	PLT ≥ 20 × 10^9^/L (days)	aGVHD (events/total)	cGVHD (events/total)
Ning, 2008, China [24]	16.25 ± 2.602 VS 15.25 ± 2.602	PLT > 50, 30 (16–45) VS 27 (15–64)	Grade I-IV, 4/9 VS 11/15; grade II-IV, 1/9 VS 8/15; grade I-II, 4/9 VS 11/15; grade III-IV, 0/9 VS 0/15	Lim, 1/7 VS 1/14; Ext, 0/7 VS 3/14
Ghavamzad, 2010, Iran [27]	14 VS 13, *p* = 0.16	16/15, *p* = 0.34	Grade III-IV, 6/25 VS 4/23; *P* = 0.73	NA
Liu, 2011, China [22]	NA	19.5 (8–52) VS 20 (10–80)	Grade I-IV, 16/27 VS 16/27; grade II-IV, 13/27 VS 9/27; grade I-II, 16/27 VS 15/27; grade III-IV, 0/27 VS 1/27	Lim, 9/27 VS 11/24; Ext, 4/27 VS 4/24
Wu, 2013b, China [28]	12 ± 2.31 VS 25.5 ± 7.94, *p* = 0.003*	30 (20–45) VS 73 (42–135), *p* = 0.004*	Grade I-IV, 4/8 VS 8/12; grade II-IV, 1/8 VS 3/12; grade I-II, 4/8 VS 7/12; grade III-IV, 0/8 VS 1/12	Lim, 1/8 VS 1/12; Ext, 0/8 VS 4/12
Mareika, 2016, Belarus [29]	19 (12–20) VS 24 (16–45), *p* = 0.09	18 (10–44) VS 23 (16–144), *p* = 0.05	Grade II-IV, 1/10 VS 3/12; grade III-IV, 0/10 VS 3/12	Ext, 1/10 VS 4/12
Xiang, 2017, China [13]	12.25 ± 1.59 VS 15.96 ± 2.20, *p* = 0.000*	15.42 ± 2.59 VS 16.02 ± 2.86, *p* = 0.382	NA	Overall, 1/32 VS 7/32; *P* = 0.023
Ball, 2007, Netherlands [16]	12 (10–17) VS 13 (9–28), *p* = 0.15	10 (9–18) VS 13 (9–100), *p* = 0.13	Grade I-IV, 2/14 VS 14/47; grade I-II, 2/14 VS 12/47; grade III-IV, 0/14 VS 2/47	Lim, 1/14 VS 4/47; Ext, 0/14 VS 2/47
Daganzo, 2009, Spain [30]	12 (10–31) VS 10 (9–36), *p*: NS	44 (27–98) VS 32 (13–97), *p*: NS	Grade I-IV, 5/9 VS 29/46; grade II-IV, 4/9 VS 11/46; grade I-II, 5/9 VS 23/46; grade III-IV, 0/9 VS 6/46	Lim, 1/8 VS 8/33; Ext, 0/8 VS 3/33
MacMillan, 2009, USA [31]	19 (8–28) VS 15 (11–30), *p* = 0.55	PLT > 50, 53 (36–98) VS 69 (31–129); *p* = 0.55	Grade II-IV, 3/8 VS 5/23	Overall, 0/8 VS 4/23; *p* = 0.23
Baron, 2010, Belgium [32]	ANC > 1, 10 VS 9; *p* = 0.2	PLT > 100, 11 VS 13; *p* = 0.7	Grade II-IV, 9/20 VS 9/16; *p*: NS	NA
Hou, 2010, China [33]	12 (11–20) VS 11.5 (11–21)	19 (11–46) VS 19 (10–78), *p* > 0.05	NA	NA
Bernardo, 2011, Italy [15]	30 (17–42) VS 28 (13–44)	32 (14–85) VS 36 (18–91), *p*: NS	Grade I-IV, 5/13 VS 21/39; grade II-IV, 4/13 VS 16/39; grade I-II, 5/13 VS 11/39; grade III-IV, 0/13 VS 10/39	Lim, 0/10 VS 1/30; Ext, 0/10 VS 2/30
Lee, 2013, Korea [14]	19 (16–21) VS 24 (17–34), *p* = 0.03*	47 (33–80) VS 57 (41–100), *P* = 0.26	Grade I-IV, 5/7 VS 5/8; grade I-II, 4/7 VS 5/8; grade III-IV, 1/7 VS 0/8	Ext, 1/7 VS 3/6; *P* = 0.27
Wu, 2013a, China [34]	11 (7–13) VS 25 (19–39), *p* = 0.02*	32 (22–41) VS 69 (55–113), *p* = 0.01*	Grade I-IV, 2/5 VS 6/9; grade I-II, 2/5 VS 5/9; grade III-IV, 0/5 VS 1/9	Lim, 1/5 VS 3/9; Ext, 0/5 VS 1/9
Xiao, 2013, China [35]	12.143 ± 2.743 VS 20.5 ± 7.653	8.714 VS 19.500, *p* < 0.01*	NA	Overall, 0/7 VS 3/8
Wang, 2015, China [36]	17 (14–21) VS 21 (19–25), *p* = 0.007*	19 (18–22) VS 21 (20–26), *p* = 0.024*	Grade I-IV, 2/7 VS 3/7; grade I-II, 2/7 VS 2/7; grade II-IV, 2/7 VS 3/7; grade III-IV, 0/7 VS 1/7	Lim, 2/7 VS 2/7; Ext, 1/7 VS 3/7
Zhang, 2015, China [37]	12 (9–22) VS 15 (9–26), *p* = 0.041*	15 (9–38) VS 16 (11–46), *p* = 0.78	Grade I-IV, 11/22 VS 16/27; grade II-IV, 3/22 VS 5/27	Lim, 3/20 VS 5/27; Ext, 1/20 VS 9/27
Kang, 2017, China [38]	14 VS 15, *p* = 0.691	20 VS 19, *p* = 0.525	Grade I-IV, 15/34 VS 12/13; grade I-II, 9/34 VS 8/13; grade III-IV, 6/34 VS 4/13	Lim, 5/34 VS 2/13; Ext, 4/34 VS 2/13
Ghavamzad, 2017, Iran [39]	NA	27.2 (22–31) VS 36.6 (22–50), *p* = 0.26	Overall, 23/41 VS 19/29	Overall, 9/41 VS 11/29

MSCs+ VS MSCs−

*Lim* limited, *Ext* extensive, *NA* not available, *NS* not significant

*Statistically significant

**Table 3:** Outcomes (OS, TRM/NRM, RR) of included studies

Survival rates with a varied follow-up time					
Author, year	Intervention group, HSCT+MSC		Control group, HSCT		*P* value
	Follow-up, months	Survival	Follow-up, months	Survival	
Ball, 2007 [16]	Range 3–28	OS 72%	Range 32–110	OS 63%	Not reported
		RR 18%		RR 26%	Not reported
Daganzo, 2009 [30]	Median 7.4, range 1–22	OS 89%	Median 24, range 1–107	OS 53%	= 0.19
		RR 11% at 22 months		RR 13% at 60 months	Not reported
		DFS 71%		DFS 45%	Not reported
		TRM 11% (95% CI 2–71)		TRM 37% (95% CI 25–54)	Not reported
Wu, 2013b [28]	Median 16.5, range 11–27	OS 75%	Median 18.5, range 12–31	OS 67%	> 0.05
		RR 25%		RR 16.67%	Not reported
		TRM 25%		TRM 33%	> 0.05
Wu, 2013a [34]	Median 27, range 24–31	OS 80%	Not reported	OS 56%	= 0.58
		TRM 0%		TRM 22.2%	= 0.51
Survival measured at fixed measure points					
Author, year	Intervention group, HSCT+MSCs		Control group, HSCT		*P* value
	Measure time point, years	Survival	Measure time point, years	Survival	
Baron, 2010 [32]	1	OS 60%	1	OS 38%	= 0.1
		DFS 5%		DFS 0%	> 0.05
		TRM 10%		TRM 37%	= 0.02*
		RR 30%		RR 25%	= 0.9
Zhang, 2015 [37]	1	OS 72.7%	1	OS 85.2%	= 0.472
		RR 9.1%		RR 22.2%	= 0.396
		CRR 9.1%		CRR 33.3%	= 0.093*
Xiang, 2017 [13]	1	OS 87%	1	OS 75%	= 0.202
		CRR 16%		CRR 25%	= 0.351
Lee, 2013 [14]	2	OS 85.7%	2	OS 55.6%	= 0.15
Liu, 2011 [22]	2	OS 69.7%	2	OS 64.3%	= 0.737
		RR 12.8%		RR 9.3%	= 0.721
MacMillan, 2009 [31]	3	OS 75% (95% CI 45–100)	3	OS 46% (95% CI 26–66)	= 0.38
Ning, 2008 [24]	3	OS 40%	3	OS 66.7%	Not reported
		DFS 30%		DFS 66.7%	= 0.035*
		RR 60.0%		RR 20%	= 0.020*
Bernardo, 2011 [15]	3	OS 63% (95% CI 43–97)	3	OS 64% (95% CI 48–79)	NS
		DFS 67% (95% CI 41–94)		DFS 56% (95% CI 40–72)	NS
		TRM 8% (95% CI 1–51)		TRM 21% (95% CI 11–38)	NS
		RR 25% (95% CI 9–67)		RR 23% (95% CI 13–42)	NS
Kang, 2017 [38]	3	OS 70.6%	3	OS 23.1	= 0.004*
		DFS 52.9%		DFS 0%	= 0*
		TRM 11.8%		TRM 46.2%	= 0.017*
		RR 32.4%		RR 53.8%	= 0.199
Ghavamzadeh, 2017 [39]	3	OS 70%	3	OS 61%	= 0.78
		DFS 54%		DFS 61%	= 0.35
Mareika, 2016 [29]	> 3	OS (90 ± 9.5)%	> 3	OS (83 ± 10.7)%	Not reported
		RR 10%		RR 0%	Not reported

*OS* overall survival, *TRM/NRM* treatment-related mortality/non-relapse mortality, *RR* relapse rate, *DFS* disease free survival, *CRR* cumulative relapse rate, *NS* not significant

*Statistically significant

**Table 4:** Outcomes (immune reconstitution) of included studies

Study	Outcome	HSCT+MSCs group	HSCT group	Time points	*p* value
Ball et al. [16]	NK cells (× 10^9^/L)	0.497, 95% CI 0.347–0.646	0.252, 95% CI 0.173–0.33	28 days	= 0.02*
	WBC > 1 × 10^9^/L (days)	11.5, 95% CI 9.0–14.8	14.9, 95% CI 10.1–26.0	NA	= 0.009*
	NK and T cells	NA	NA	3 months	NS
Liu et al. [22]	WBC (days)	12 (10–21)	12 (10–23)	NA	NS
Ghavamzadeh et al. [33]	WBC > 0.5 × 10^9^/L (days)	17.7 (15–20)	17.7 (15–20)	NA	= 0.83
Mareika et al. [29]	(NK) cells (× 10^9^/L)	0.1 (0.02–0.23)	0.01 (0.002–0.06)	100 days	= 0.07
	B cells (× 10^9^/L)	0.18 (0.11–0.43)	0.07 (0.02–0.19)	100 days	= 0.05*
Lee et al. [14]	Lymphocyte subpopulations	NA	NA	28, 100, 180 and 365 days	NS
Gonzalo-Daganzo et al. [30]	Lymphocyte subpopulations	NA	NA	NA	NS
Baron et al. [32]	T cells (× 10^9^/L)	0.29 (0.00 4–0.54)	0.202 (0.041–0.886)	28 days	NS
Wu et al. [34]	Absolute lymphocyte count (× 10^9^/L)	0.305 (0.196–0. 445)	0.256 (0.150–0.368)	30 days	= 0.35
Xiang [13]	The levels of serum immunoglobulins IgA, IgG, IgM, and IgE			1, 3, and 6 months	< 0.05*
	The levels of T lymphocytes and B cells				

*NA* not available, *NS* not significant

*Statistically significant

### Methodological quality assessment

The methodological quality of the studies was critically assessed in collaboration between the first two authors. For the RCTs, the Cochrane Collaboration tool for assessing the risk of bias (RoB) in randomized trials was applied [40], and an adapted RoB form adjusted to fit the non-randomized design was used for the nRCTs [18]. Random sequence generation and allocation concealment were appraised for RCTs while comparisons between groups and criteria for selecting participants were evaluated for nRCTs [18]. All studies were appraised for blinding, selective reporting and incomplete outcome data with a three-point scale (low RoB, high RoB, or unclear RoB). Inconsistencies between investigators were resolved by discussion.

### Statistical analysis

This meta-analysis was performed with Review Manager (RevMan) software version 5.4. Considering the apparent heterogeneity, we performed separate meta-analyses for the 6 RCTs and 13 nRCTs. A two-sided *p* value ≤ 0.05 was considered statistically significant. For the outcome of engraftment, we chose to report standard mean differences (SMDs). As only median values were documented in most articles, except for studies of Xiang [13] and Xiao et al. [35], we used the range and total number to calculate the mean day of engraftment with the method described by Luo et al. [41]. For OS, the log hazard ratios (HRs) and standard errors were directly extracted from the studies, transformed from the presented *p* values and events, or indirectly converted from the displayed Kaplan–Meier curves [42]. We merged the log HRs and corresponding 95% confidence intervals (CIs) of each study using the generic inverse-variance method. Statistical heterogeneity across studies was evaluated using the chi-square-based *Q* test with a significant level of *p* <  0.1 and quantified with *I*^2^ statistic (*I*^2^ > 50% indicated high heterogeneity) [43]. If heterogeneity was not significant, a fixed-effect model was adopted for synthesis; otherwise, a random-effect model was employed [21]. Finally, subgroup analyses were conducted based on patients’ clinical characteristics, including type of disease, HLA matching, and average age.

## Results

### Search results and characteristics of the studies

As shown in Fig. 1 and Table 1, the titles and abstracts of 959 records were screened. Of these records, 41 publications were selected for full-text review and appraised for eligibility. Seven additional reports were retrieved by checking references cited in these articles. Finally, 19 studies, with 13 published in English and 6 in Chinese, met all preset inclusion criteria for this review [13–16, 22, 24, 27–39]. These studies comprised 16 full publications and 3 meeting abstracts. Ten were conducted in China, 5 in Europe, 1 in the USA, 2 in Iran, and 1 in Korea. Six of the 19 studies were RCTs with 234 participants, and the remaining 13 trials were sorted as nRCTs including 7 prospective controlled studies with 203 patients and 6 historical controlled studies investigating 291 participants. Ten trials assessed adolescents or children, seven examined adults, one trial involved patients aged 3 to 48 years old, and 1 trial did not mention the average age of participants. Two trials were conducted in patients with β-thalassaemia, one in patients with severe aplastic anemia (SAA), thirteen in patients with hematological malignancies, and the remaining 3 trials in patients with malignant or non-malignant hematonosis together. Six trials administered UCB HSCs; six trials delivered PB HSCs; four trials included patients who received UCB, PB, or BM HSCs; and 3 trials did not report the source of HSCs. Four trials infused HLA-identical HSCs, thirteen trials delivered HLA non-identical HSCs, and the remaining 2 trials did not mention this parameter. MSCs were extracted from BM in 9 trials and UCB in 8 trials, and two trials did not describe the source of MSCs. In the intervention arms, MSCs were administered at a dose of less than 1 × 10^6^ cells/kg in 4 trials, between 1 × 10^6^ and 5 × 10^6^ cells/kg in 11 trials, and more than 5 × 10^6^ cells/kg in 2 trials, and the other 2 trials did not provide information about the dose. The median durations of follow-up were within 1 year in 2 trials, between 1 and 2 years in 7 trials, between 2 and 3 years in 4 trials, and 3 years or more in 4 trials. In the remaining two trials, the durations of follow-up varied substantially between groups, namely, 3 to 28 and 7.4 (1–22) months in the MSC groups and 32 to 110 and 24 (1–107) months in the control groups, respectively.

All patients received intensive conditioning regimens, GVHD prophylaxis, and supportive care. Patients in the control groups underwent HSCT alone. Patients in the experimental groups received a co-infusion of MSCs with allogeneic HSCs, both of which were administrated within day “0” whenever allowed by the patient’s condition, otherwise within the next 24 h. The main outcomes were engraftment of platelets and neutrophils, and acute and chronic GVHD. The secondary outcomes included the relapse rate, overall survival, non-relapse mortality or treatment-related mortality, and immune reconstitution.

### Risk of bias analysis

**Fig. 2:**
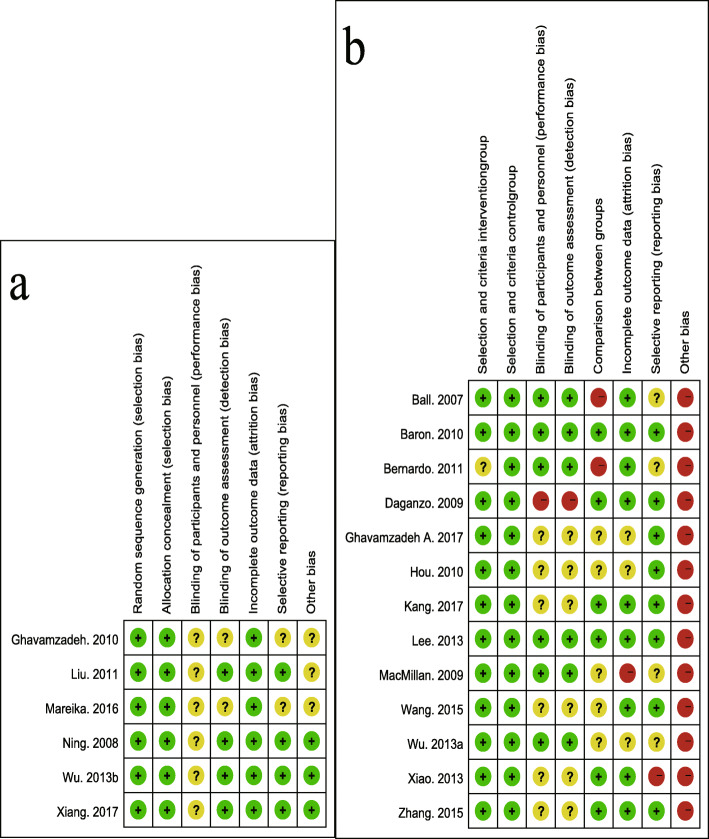
Risk of Bias (RoB) judgment. RoB assessments for the included **a**) six RCTs and **b** thirteen nRCTs. Green sign means low RoB, yellow sign means unclear RoB, red sign means high RoB

Figure 2 shows the results of the quality assessment for each study. No trial included more than 100 participants and 5 trials recruited fewer than 20 patients [14, 28, 34–36]. Most studies among the 6 RCTs had a low risk of bias, but the 13 nRCTs had a higher risk of bias according to the predefined methodological quality assessment tool.

### Engraftment

**Fig. 3:**
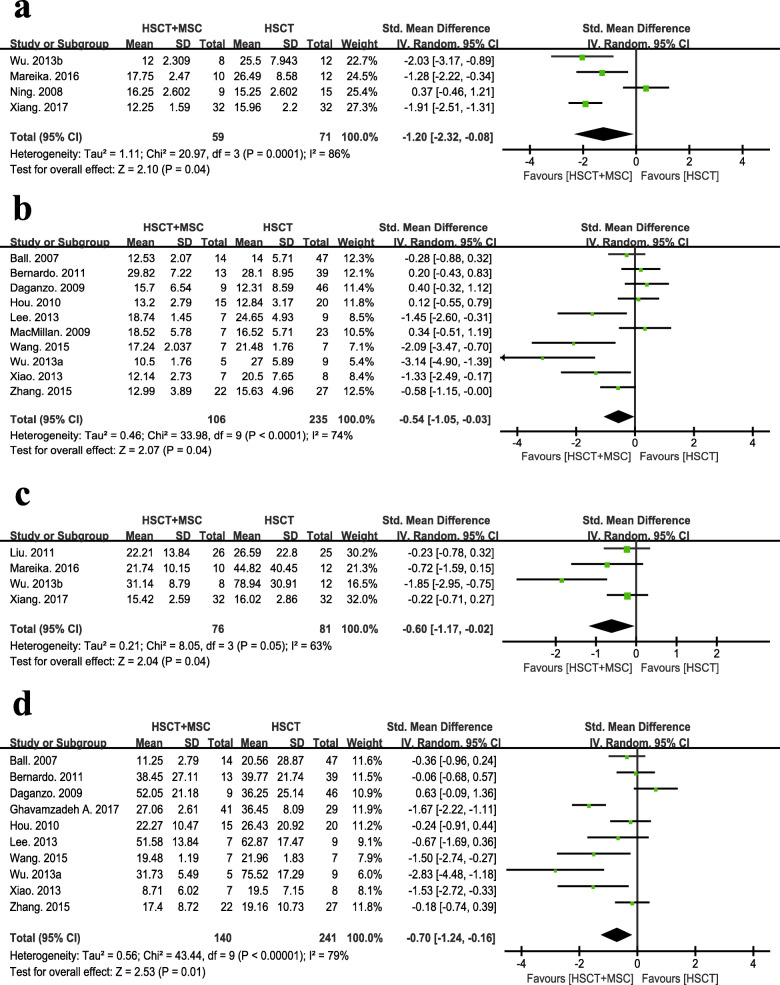
Assessment of engraftment between HSCT+MSC group and HSCT alone group. **a** Neutrophil engraftment in RCTs. **b** Neutrophil engraftment in nRCTs. **c** Platelet engraftment in RCTs. **d** Platelet engraftment in nRCTs

Four RCTs and 10 nRCTs provided sufficient information on the neutrophil engraftment that ranged from 10.5 to 29.82 mean days in the experimental group and from 12.31 to 28.1 mean days in the control group. We estimated the means and standard deviations (SD) from the given sample sizes, medians, and ranges [41]. The studies of Ghavamzadeh et al. [27] and Kang et al. [38] were not included in the meta-analysis of these results, as they only supplied the median values. Data from the study of Baron et al. [32] were excluded because a neutrophil count of ≥ 1.0 × 10^9^ cells/L was defined as the standard of neutrophil engraftment. The results from the studies of Liu et al. [22] and Ghavamzadeh et al. [39] were not included because they monitored white blood cell engraftment instead of neutrophil engraftment. Both meta-analysis of RCTs with 130 participants and nRCTs with 341 participants indicated that patients who received MSC co-infusion needed a shorter time to reach neutrophil recovery than the control group (4 RCTs: SMD − 1.20, 95% CI − 2.32 to − 0.08, *p* = 0.04, *I*^2^ = 86%; 10 nRCTs: SMD − 0.54, 95% CI − 1.05 to − 0.03, *p* = 0.04, *I*^2^ = 74%; Fig. 3a, b).

Regarding platelet engraftment, we did not include the studies of Ghavamzadeh et al. [27] and Kang et al. [38] due to a lack of usable information; data from the studies of Ning et al., Baron et al., and MacMillan et al. [24, 31, 32] were not included because they defined platelet counts of ≥ 100 × 10^9^ cells/L or ≥ 50 × 10^9^ cells/L as the standard of platelet recovery. As a result, only 4 RCTs and 10 nRCTs were selected for synthesis, and the mean engraftment time ranged from 8.71 to 52.05 days for the MSC co-infused groups and from 16.02 to 78.94 days for the control groups. Both RCTs with 157 participants and nRCTs with 381 participants revealed that the patients from the MSC co-infusion group experienced a faster recovery of platelets than patients from the HSCT alone group (4 RCTs: SMD − 0.60, 95% CI − 1.17 to − 0.02, *p* = 0.04, *I*^2^ = 63%; 10 nRCTs: SMD − 0.70, 95% CI − 1.24 to − 0.16, *p* = 0.01, *I*^2^ = 79%; Fig. 3c, d).

According to the funnel plots present in Additional file 3, publication bias was obvious for the outcomes of neutrophil and platelet engraftment. Meanwhile, statistical heterogeneity existed, as indicated by the Tau^2^, Chi^2^, and *I*^2^ tests (Fig. 3a–d). Thus, a random-effect model was adopted. Sensitivity was also assessed using leave-one-out analyses to estimate the relative contribution of each single study to the overall heterogeneity. When the study of Wu et al. [28] was abandoned, the score of *I*^2^ decreased sharply from 63 to 0% in the meta-analysis of PLT engraftment in RCTs, which may be because it was the only trail that used MSCs in excess of 5 × 10^6^ cells per kilogram; no significant changes in *I*^2^ values were observed in the other 3 meta-analyses.

In addition, we conducted subset analyses (details are shown in Additional file 4) according to the dose of MSCs, and all the studies were sorted into three groups. The study of Xiao et al. [35] was excluded because it failed to document the dosage of MSCs. These analyses showed that with the increasing of MSC dose, hematopoietic recovery was more effective, although the results of subsets of nRCTs with MSC doses ranging from 1 × 10^6^ to 5 × 10^6^ cells per kilogram were not obvious, which may be partially attributed to the potential confounders of the study design. However, as the number of included studies and sample size in some subgroups were limited, all these results should be interpreted with caution.

### GVHD

**Fig. 4:**
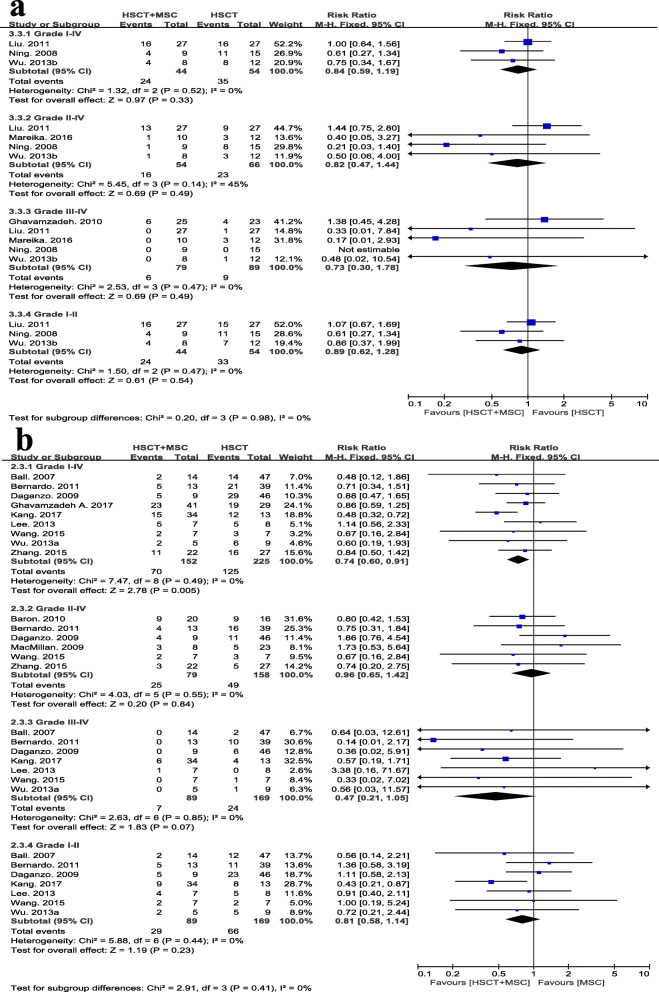
Assessment of overall and graded incidence of aGVHD between HSCT+MSC group and HSCT alone group. Analysis was performed for **a** RCTs and **b** nRCTs separately

The incidence of grade I-IV aGVHD was reported in 3 RCTs [22, 24, 28]. The study of Ghavamzadeh et al. [27] only provided the data on grade III-IV aGVHD; Mareika et al. [29] covered the occurrences of grade II-IV and grade III-IV aGVHD; the study of Xiang [13] was not included, as it did not report this outcome. Among the 13 nRCTs, nine studies reported the incidence of overall aGVHD; two [31, 32] provided only the data for grade II-IV aGVHD; and the remaining 2 studies [33, 35] did not mention this outcome. Regarding the incidence of grade I-IV aGVHD, no statistically significant differences were observed in the meta-analysis including 3 RCTs with 98 cases (RR 0.84, 95% CI 0.59 to 1.19, *p* = 0.33, *I*^2^ = 0%; Fig. 4a). In the meta-analysis including 9 nRCTs with 377 participants, patients treated with a MSC co-infusion had a significantly lower rate of grade I-IV aGVHD incidence than the control group (RR 0.74, 95% CI 0.60 to 0.91, *p* = 0.005, *I*^2^ = 0%; Fig. 4b). No difference was observed between groups in outcomes of grade II-IV, grade III-IV, and grade I-II aGVHD from either the meta-analysis of RCTs or with the meta-analysis of nRCTs (Fig 4a, b).

**Fig. 5:**
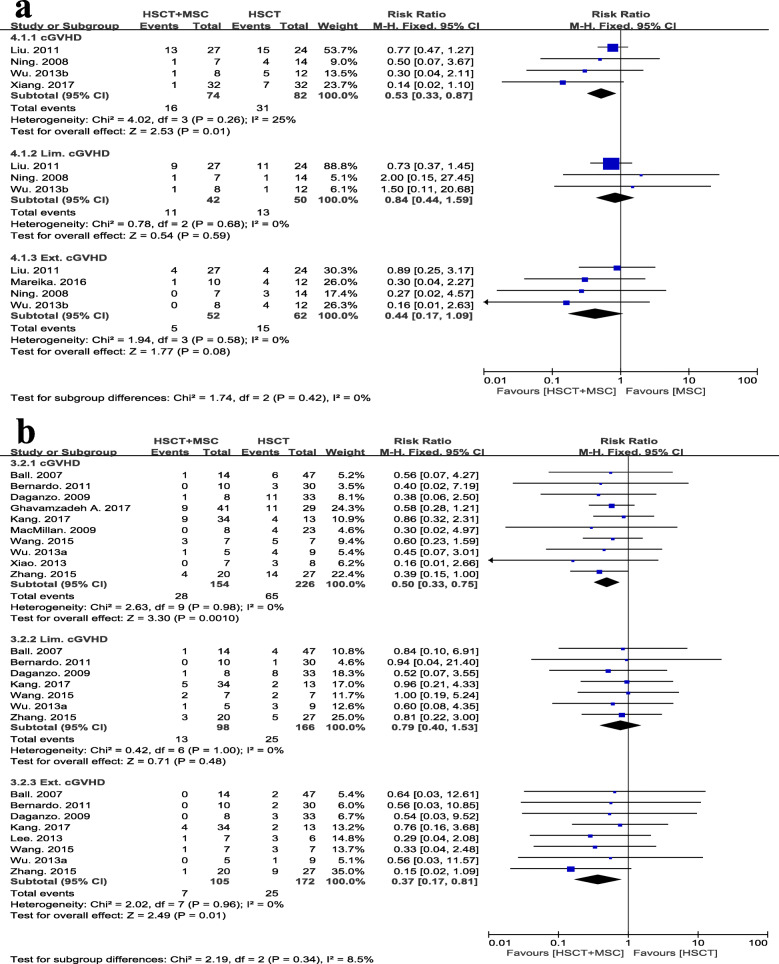
Assessment of overall and graded incidence of cGVHD between HSCT+MSC group and HSCT alone group. Analysis was performed for **a** RCTs and **b** nRCTs separately. Lim., limited; Ext., extensive

Among the 19 candidate studies, three [27, 32, 33] did not provide information about cGVHD incidence, and two [14, 29] presented only the incidence of extensive cGVHD without data on the number of people who developed limited cGVHD. As a result, 4 RCTs with 156 participants (RR 0.53, 95% CI 0.33 to 0.87, *p* = 0.01, *I*^2^ = 25%; Fig. 5a) and 10 nRCTs with 380 participants (RR 0.50, 95% CI 0.33 to 0.75, *p* = 0.001, *I*^2^ = 0%; Fig. 5b) were separately included in meta-analysis for the overall occurrence of cGVHD, and the results of both analyses of RCTs and nRCTs suggested that the MSC co-infusion significantly reduced the overall incidence of cGVHD. Specifically, regarding the outcome of limited cGVHD, no statistically significant difference was found between groups. However, patients in the MSC group had a lower risk of extensive cGVHD than patients in the control groups, as evidenced by the results of the meta-analysis with 8 nRCTs (RR 0.37, 95% CI 0.17, 0.81, *I*^2^ = 0%, *p* = 0.01; Fig. 5b); moreover, a slight trend was observed in the meta-analysis of 4 RCTs (RR 0.44, 95% CI 0.17, 1.09, *I*^2^ = 0%, *p* = 0.08; Fig. 5a).

Both funnel plots for aGVHD and cGVHD (Additional file 5) showed asymmetry, indicating the existence of publication bias. The heterogeneity was not statistically significant, as indicated by the Tau^2^, Chi^2^, and *I*^2^ tests.

### Overall survival

**Fig. 6:**
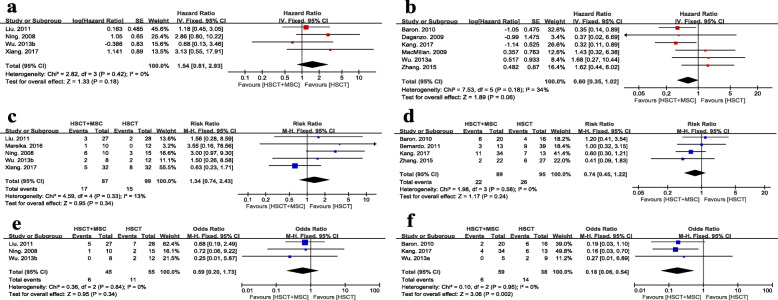
Assessment of OS, RR and TRM/NRM between HSCT+MSC group and HSCT alone group. **a** Forest plot of RCTs on overall survival. **b** Forest plot of nRCTs on overall survival. **c** Forest plot of RCTs on relapse rate. **d** Forest plot of nRCTs on relapse rate. **e** Forest plot of RCTs on non-relapse mortality. **f** Forest plot of nRCTs on non-relapse mortality

Fourteen trials estimated the effect of the MSC co-infusion on the overall survival of HSCT recipients, of which four studies [14–16, 39] were excluded because they failed to provide sufficient information that could be transformed into logHR and SE values. The outcomes of the meta-analyses of the remaining 4RCTs (164 participants) and 6 nRCTs (231 participants) (HR 1.54, 95% CI 0.81 to 2.93, *p* = 0.18, *I*^2^ = 0%, Fig. 6a; HR 0.60, 95% CI 0.35 to 1.02, *p* = 0.06, *I*^2^ = 34%, Fig. 6b; respectively) did not suggest a statistically significant difference in OS between the groups. Publication bias was not obviously visualized from the funnel plot of OS, which is shown in Additional file 6a. Heterogeneity was not statistically significant, as indicated by the Tau^2^, Chi^2^, and *I*^2^ tests.

### Relapse rate

Eleven studies reported the occurrence of relapse in both the MSC group and the control group, but studies of Ball et al. [16] and Gonzalo-Daganzo et al. [30] were excluded because of varying follow-up times between groups. Both the meta-analyses of 5 RCTs with 186 participants and 4 nRCTs with 184 participants (RR 1.34, 95% CI 0.74 to 2.43, *p* = 0.34, *I*^2^ = 13%, Fig. 6c; RR 0.74, 95% CI 0.45 to 1.22, *p* = 0.24, *I*^2^ = 0%, Fig. 6d; respectively) suggested no difference between patients who did or did not receive MSC co-transplantation. Apparent publication bias was observed from the funnel plot of RR (Additional file 6b), and heterogeneity was not statistically significant, as indicated by the Tau^2^, Chi^2^, and *I*^2^ tests.

### TRM/NRM

For the TRM/NRM, the results of meta-analyses varied between the 3 RCTs with 100 participants and the 3 nRCTs with 97 participants (OR 0.59, 95% CI 0.20 to 1.73, *p* = 0.34, *I*^2^ = 0%, Fig. 6e; OR 0.18, 95% CI 0.06 to 0.54, *p* = 0.002, *I*^2^ = 0%, Fig. 6f; respectively). However, none of the odds ratios (ORs) was greater than 1, and no statistically significant heterogeneity was observed. We concluded that a slight trend existed in which patients infused with MSCs may have a lower risk of TRM/NRM. The funnel plot was not applicable due to the small number of studies. The heterogeneity was not statistically significant, as indicated by the Tau^2^, Chi^2^, and *I*^2^ tests.

### Immune reconstitution

Multiple studies have investigated the effect of MSC co-transplantation in allo-HSCT on immune reconstitution. However, all these data from each trial were difficult to combine because of the use of various parameters and time points, and we only provide a qualitative description. In the study of Ball et al., the recovery time of NK cells at 28 days post transplantation was faster in recipients who received the MSC co-infusion than in the controls [16]. The study of Xiang [13] showed that the levels of lymphocyte subpopulations and immunoglobulins in patients treated with MSCs were significantly higher than those in the control group at the 1st, 3rd, and 6th months after transplantation. The other seven trials [14, 22, 29, 30, 32, 34, 39] did not observe differences in the lymphocyte recovery time after transplantation between the two groups (details are shown in Table 4).

### Analyses of patient subgroups

Finally, we conducted subgroup analyses based on the type of disease (malignant versus non-malignant), HLA matching (HLA-identical versus non-identical), and average age (children and adolescents versus adults) by combining all the RCTs and nRCTs together (detailed forest plots are presented in Additional files 7, 8, 9, 10, 11, 12, 13; summarized results are shown in Table 6).

For patients with malignancies, the risks of developing aGVHD (RR 0.77, 95% CI 0.63 to 0.95, *p* = 0.02, Fig. S7a), cGVHD (RR 0.51, 95% CI 0.35 to 0.75, *p* < 0.01, Fig. S8a), and NRM (OR 0.34, 95% CI 0.16 to 0.73, *p* < 0.01, Fig. S11a) were significantly reduced in the MSC co-transplantation group. For patients with non-malignant blood diseases, MSC co-transplantation accelerated hematopoietic recovery in terms of both neutrophil and platelet engraftment (SMD − 2.09, 95% CI − 3.47 to − 0.70, *p* < 0.01, Fig. S5a; SMD − 1.64, 95% CI − 2.15 to − 1.14, *p* < 0.01, Fig. S6a; respectively).

Recipients receiving HLA-identical HSCT benefited in terms of the outcome of cGVHD (RR 0.43, 95% CI 0.22 to 0.84, *p* = 0.01, Fig. S8b) from MSC co-transplantation but had a lower OS (HR 2.95, 95% CI 1.05 to 8.25, *p* = 0.04, Fig. S9b). For patients who received HLA non-identical HSCT, MSC co-transplantation exerted a significant positive effect on the outcomes of hematopoietic reconstitution (SMD − 0.63, 95% CI − 1.18 to − 0.07, *p* = 0.03, Fig. S5b; SMD − 0.50, 95% CI − 0.93 to − 0.07, *p* = 0.02, Fig. S6b; neutrophil and platelet engraftment, respectively) and significantly reduced the risk of developing GVHD (RR 0.76, 95% CI 0.61 to 0.94, *p* = 0.01, Fig. S7b; RR 0.56, 95% CI 0.39 to 0.81, *p* < 0.01, Fig. S8b; aGVHD and cGVHD respectively) and TRM/NRM (OR 0.31, 95% CI 0.14 to 0.68, *p* < 0.01, Fig. S11b).

Adults who received MSC co-transplantation showed a lower incidence of cGVHD (RR 0.54, 95% CI 0.36 to 0.81, *p* < 0.01, Fig. S8c) than those who did not. Among children and adolescents, MSC co-transplantation obviously improved hematopoietic reconstitution (SMD − 1.07, 95% CI − 1.84 to − 0.31, *p* < 0.01, Fig. S5c; SMD − 0.71, 95% CI − 1.22 to − 0.19, *p* < 0.01, Fig. S6c; neutrophil and platelet engraftment, respectively) and reduced the risk of developing GVHD (RR 0.64, 95% CI 0.47 to 0.87, *p* < 0.01, Fig. S7c; RR 0.43, 95% CI 0.23 to 0.84, *p* = 0.01, Fig. S8c; aGVHD and cGVHD, respectively) and TRM/NRM (OR 0.19, 95% CI 0.05 to 0.68, *p* = 0.01, Fig. S11c).

## Discussion

Allo-HSCT is the curative modality for many hematological diseases, and the optimization of the whole process is an important project. The main objective of our study was to determine whether MSC co-transplantation with allo-HSCT in patients with hematological conditions could improve transplantation outcomes. The results of our meta-analyses (shown in Table 5) indicated that MSC co-transplantation generally facilitates the engraftment of neutrophils and platelets and reduces the risk of cGVHD incidence but does not alter the risk of mortality or relapse, with a slightly positive trend towards reducing the risk of aGVHD incidence and NRM. These findings corroborated the findings of previous preclinical studies that MSCs, as progenitor cells of bone marrow stroma, enhanced HSC engraftment [44] and prevented lethal GVHD through immune modulation [45, 46] when co-transplanted with allo-HSCT in animal models. Based on the results of our subgroup analyses, co-transplantation of MSCs appears to be an optimal treatment strategy for children and adolescents who receive HLA non-identical HSCT. Notably, in the subgroup of HLA-identical transplants recipients, the OS rate was significantly lower in recipients of MSC co-infusion than in those without the MSC co-infusion, and both of the included studies were conducted in patients with hematological malignancies [13, 24]. Thus, we should be cautious about relapse when considering whether to use MSCs in patients with hematological malignancies who are receiving HAL-identical HSCT, although this evidence is based on limited studies (Table 6).

**Table 5:** Meta-analysis of the safety and efficacy of MSCs co-infused in allo-HSCT

Outcome	Study design	Number of Trials	Sample size	SMD/RR/OR/HR	95% CI	***P***	***I***^**2**^
ANC	RCTs	4	130	SMD, − 1.20	− 2.32, − 0.08	0.04*	86%^#^
	nRCTs	10	341	SMD, − 0.54	− 1.05, − 0.03	0.04*	74%^#^
PLT	RCTs	4	157	SMD, − 0.60	− 1.17, − 0.02	0.04*	63%^#^
	nRCTs	10	381	SMD, − 0.70	− 1.24, − 0.16	0.01*	79%^#^
aGVHD	RCTs	3	98	RR, 0.84	0.59, 1.19	0.33	0%
	nRCTs	9	377	RR, 0.74	0.60, 0.91	0.005*	0%
cGVHD	RCTs	4	156	RR, 0.53	0.33, 0.87	0.01*	25%
	nRCTs	10	380	RR, 0.50	0.33, 0.75	0.001*	0%
OS	RCTs	4	164	HR, 1.54	0.81, 2.93	0.18	0%
	nRCTs	6	231	HR, 0.60	0.35, 1.02	0.06	34%
RR	RCTs	5	186	RR, 1.34	0.74, 2.43	0.34	13%
	nRCTs	4	184	RR, 0.74	0.45, 1.22	0.24	0%
NRM	RCTs	3	100	OR, 0.59	0.20, 1.73	0.34	0%
	nRCTs	3	97	OR, 0.18	0.06, 0.54	0.002*	0%

*The difference was statistical significance

^#^Significant heterogeneity needed to conduct subgroup analysis

**Table 6:** Summarized results of the subgroup analysis

Outcome or subgroup	ANC				PLT				aGVHD				cGVHD				OS				RR				NRM			
	No. of studies	Sample size	SMD (95% CI)	***P***	No. of studies	Sample size	SMD (95% CI)	***P***	No. of studies	Sample size	RR (95% CI)	***P***	No. of studies	Sample size	RR (95% CI)	***P***	No. of studies	Sample size	HR (95% CI)	***P***	No. of studies	Sample size	RR (95% CI)	***P***	No. of studies	Sample size	OR (95% CI)	***P***
**Subtotal**	14	471	− 0.77 (− 1.29, − 0.26)	< 0.01*	14	538	− 0.66 (− 1.06, − 0.27)	< 0.01*	12	475	0.77 (0.64, 0.92)	< 0.01*	14	535	0.51 (0.37, 0.70)	< 0.01*	10	395	0.88 (0.59, 1.33)	0.54	9	370	0.96 (0.66, 1.41)	0.84	6	197	0.33 (0.16, 0.71)	< 0.01*
**Type of disease**																												
**Hematologic malignant**	10	367	− 0.54 (− 1.14, 0.06)	0.08	9	364	− 0.28 (− 0.61, 0.04)	0.09	8	316	0.77 (0.63, 0.95)	0.02*	9	361	0.51 (0.35, 0.75)	< 0.01*	9	381	0.85 (0.56, 1.30)	0.45	9	370	0.96 (0.66, 1.41)	0.84	5	183	0.34 (0.16, 0.73)	< 0.01*
**Nonmalignant disorders**	1	14	− 2.09 (− 3.47, − 0.70)	< 0.01*	2	84	− 1.64 (− 2.15, − 1.14)	< 0.01*	2	84	0.83 (0.58, 1.21)	0.34	2	84	0.58 (0.32, 1.06)	0.08	0	0	NA	NA	0	0	NA	NA	0	0	NA	NA
**Mixed**	3	90	− 1.39 (− 2.86, 0.07)	0.06	3	90	− 1.41 (− 2.79, − 0.03)	0.05*	2	75	0.53 (0.21, 1.33)	0.17	3	90	0.38 (0.11, 1.28)	0.12	1	14	1.68 (0.27, 10.44)	0.58	0	0	NA	NA	1	14	0.27 (0.01, 6.89)	0.43
**HLA matching**																												
**Identical**	2	88	− 0.79 (− 3.02, 1.45)	0.49	2	134	− 0.94 (− 2.36, 0.49)	0.2	2	94	0.79 (0.56, 1.11)	0.18	3	155	0.43 (0.22, 0.84)	0.01*	2	89	2.95 (1.05, 8.25)	0.04*	2	89	1.34 (0.29, 6.25)	0.71	1	25	0.72 (0.06, 9.22)	0.80
**Non-identical**	10	346	− 0.63 (− 1.18, − 0.07)	0.03*	10	367	− 0.50 (− 0.93, − 0.07)	0.02*	10	381	0.76 (0.61, 0.94)	0.01*	10	365	0.56 (0.39, 0.81)	< 0.01*	8	306	0.70 (0.45, 1.10)	0.12	6	259	0.81 (0.51, 1.28)	0.37	5	172	0.31 (0.14, 0.68)	< 0.01*
**Not reported**	2	37	− 1.30 (− 2.03, − 0.57)	< 0.01*	2	37	− 1.02 (− 1.77, − 0.26)	< 0.01*	0	0	NA	NA	1	15	0.16 (0.01, 2.66)	0.20	0	0	NA	NA	1	22	3.55 (0.16, 78.56)	0.42	0	0	NA	NA
**Average age**																												
**≤ 18 years**	8	279	− 1.07 (− 1.84, − 0.31)	< 0.01*	7	249	− 0.71 (− 1.22, − 0.19)	< 0.01*	6	209	0.64 (0.47, 0.87)	< 0.01*	7	276	0.43 (0.23, 0.84)	0.01*	5	175	0.94 (0.39, 2.22)	0.88	5	205	0.79 (0.49, 1.30)	0.36	3	81	0.19 (0.05, 0.68)	0.01*
**> 18 years**	6	192	− 0.37 (− 1.00, 0.26)	0.25	6	219	− 0.35 (− 0.86, 0.17)	0.19	5	196	0.85 (0.65, 1.12)	0.25	6	189	0.54 (0.36, 0.81)	< 0.01*	5	220	1.01 (0.44, 2.30)	0.98	4	165	1.25 (0.68, 2.31)	0.47	3	116	0.46 (0.18, 1.18)	0.11
**Not reported**	0	0	NA	NA	1	70	− 1.67 (− 2.22, − 1.11)	< 0.01*	1	70	0.86 (0.59, 1.25)	0.42	1	70	0.58 (0.28, 1.21)	0.15	0	0	NA	NA	0	0	NA	NA	0	0	NA	NA

In addition to the therapeutic effect of intensive chemoradiotherapy, the potent GVL effects mediated by donor immunity also attribute to the success of allo-HSCT in patients with hematological malignancies [47]. Compared with other HLA-matched transplants, patients receiving HLA-identical HSCT have a much lower incidence of rejection or GVHD and exhibit weaker GVL effects [47, 48], which might be further suppressed by infused MSCs, thus possibly leading to a higher risk of relapse [47]. The tumor-related effects of MSCs on hematological malignancies are not well understood [3]. Among multiple preclinical studies, some reported proliferative activity [49], while others suggested inhibitory effects [3]. For instance, MSCs were found to exert an antitumor effect on leukemic cells through extracellular vesicle, which contain various molecules, such as proteins, microRNA, mRNA, and siRNA, from their parental cells, and all these molecules can be transferred to cancer cells [3, 50]. Another study [49] reported a contradictory effect of human MSCs on tumor cell growth in vitro and in vivo. The animal study showed that human MSCs enhanced tumor formation and growth, which may be related to the increase in tumor vessel formation, while the in vitro study revealed that cocultured human MSCs inhibited the proliferation and induced the apoptosis of tumor cells [49]. Currently, the predominant hypothesis is that MSCs function as a “double-edged sword” through signaling pathways to suppress cancerous cell proliferation and apoptosis [3, 51].

A 20-year-old female patient with acute myeloid leukemia showed rapid engraftment and no acute or chronic GVHD after receiving haploidentical peripheral blood stem cells combined with MSCs [52]. The patient was doing well 31 months after the allo-HSCT, indicating an encouraging efficacy of MSCs co-transplanted with HLA-haploidentical HSCT in young people with blood malignancies. Recently, a multicenter clinical trial [53] also documented the efficacy and safety of infusing BM-MSCs combined with haplo-HSCT in children with SAA because this approach promoted hematopoietic implantation, protected against severe aGVHD and prevented cGVHD. All these clinical findings were consistent with our results.

A previous meta-analysis [18] that evaluated the role of MSC co-transplantation in allo-HSCT in 309 participants did not reveal any statistically significant effects. In addition to the 3 RCTs and 6 nRCTs included in the previous meta-analysis, we also included an additional 3 RCTs [13, 27, 29] and 7 nRCTs [30, 31, 35–39], with a total of 728 participants because of time reasons and the inclusion of Chinese articles. With much larger sample sizes and recently published studies, the results of our meta-analysis may be more reliable. In addition, our meta-analysis reported more outcomes, including PLT engraftment, OS, and RR. Furthermore, we performed subgroup analyses according to the patients’ disease, age, and HLA-matching. Two relevant meta-analyses [19, 54] pooled studies in which MSCs were co-transplanted with HSCs and MSCs were infused post the transplantation of HSCs, mainly to explore the prophylactic efficacy of MSCs for GVHD. Zhao et al. [54] categorized the studies of Liu et al. [22] and Xiang [13] into subgroup of after infusion, leaving only 2 trials [24, 28] in the co-infusion subgroup. Compared with results from this co-infusion subgroup analysis, our meta-analysis appears to be more powerful due to the adequate number of included studies. Wang et al. [19] did not observe a significant difference in the outcome of cGVHD (RR 0.52, 95% CI 0.24 to 1.14, *p* = 0.259, participants = 156) in the subgroup of MSC co-infused, which was possibly attributed to the adoption of the random effects model. With an *I*^2^ score of 25%, we employed a fixed-effects model for our study. Consequently, by analyzing the same 4 RCTs that were included in the meta-analysis by Wang, our results indicated that MSC co-infusion significantly reduced the incidence of cGVHD (RR 0.53, 95% CI 0.33 to 0.87, *p* = 0.01, participants = 156). Furthermore, our meta-analysis of 10 other nRCTs (RR 0.50, 95% CI 0.33 to 0.75, *p* < 0.01, participants = 380) yielded consistent results. In addition to cGVHD and OS, we evaluated more outcomes, including ANC and PLT engraftment, aGVHD, and immune recovery. Overall, regarding the safety and efficacy of MSCs co-transplanted in allo-HSCT recipients, our study provided more comprehensive and robust evidence than previous similar meta-analyses.

In addition to the patient’s condition, more knowledge of MSCs, such as the tissue source and immunogenicity of MSCs, dose, routes of delivery, and infusion timing of MSCs, should improve the overall therapeutic efficacy and potency of MSC co-transplantation in allo-HSCT. Although most of the clinical information published to date has been produced using culture-expanded marrow-derived MSCs, some information shows that MSCs from BM, UC, adipose tissue, and placenta possess a similar functional potential [9, 55–57], indicating that the antitumor effect of MSCs may not depend on their tissue source and origin [9]. Another single-cell RNA sequencing study of equine MSCs revealed both inter- and intra-source heterogeneity across different sources of MSCs, indicating that some MSC subgroups may have advantageous biological functions [58]. It was reported that UC-derived MSCs exerted superior prophylactic effects against cGVHD than did BM-MSCs [19]. However, even after multiple administrations, human allogeneic MSCs do not trigger a strong immune response that causes rejection [9]. Regardless of conventional graft-versus-host compatibility considerations, this ultimate immune rejection of donor MSCs does not affect the clinical efficacy of MSCs, as suggested by abundant preclinical and clinical studies in allogeneic MSCs [59].

MSCs exert their functions, such as proliferation, secreted factors, antitumor effects, and cytotoxicity, in a dose-dependent manner [60], suggesting that MSCs produce a better effect when administered at relatively high doses [19, 60]. According to previous studies, high doses of MSCs are inhibitory in mixed lymphocyte cultures, while low doses promote lymphocyte proliferation [48]. However, this dose-response effect may not be beneficial when the MSC dose is above a certain threshold [60, 61]. Numerous routes of MSC delivery to the human body have been used, including intramuscular or direct injection into tissues or organs [62, 63], intravenous (IV) infusion [63–65], intraarterial (IA) infusion [63, 66], and intra-bone marrow injection [67, 68]. Topical application of MSCs has been widely used in burn medicine and wound care [63]. In the field of transplantation, the IV injection of MSCs is the most common method to apply MSCs [63]. However, this approach might result in a large number of MSCs being trapped in the lungs and a limited number of cells reaching the arterial system [65]. In addition, IV infusion may facilitate the rapid clearance of clinical MSCs by innate host immune cells [63]. Researchers have questioned whether the remaining number of cells homing to the bone marrow could produce therapeutic effect. By inhibiting MSC CD49 or administrating 2 boluses instead of a single bolus, pulmonary MSC passages were increased significantly following IV infusion [65]. Considerations specific to IV delivery concern cells generating emboli or clots [63]. IA infusion of MSCs allows mass cells to home to the bone marrow to produce therapeutic effects, and improved engraftment efficiency has been observed in previous studies [69]. However, the IA delivery of MSCs for stroke is associated with a potential risk of cerebral infarction caused by microembolisms and decreased cerebral blood flow, according to a survey of published results [66]. Although the intra-bone marrow injection of whole BM cells (both pluripotent HSCs and MSCs) was thought to be a valuable strategy for allogeneic stem cell transplantation [67], no major conclusions have been reached regarding the optimal method for MSC delivery [63].

The timing and duration of MSC infusion is another important factor to consider when evaluating the therapeutic efficacy of MSCs in allo-HSCT. Our research shows that MSC co-transplantation significantly improved the engraftment of both neutrophils and platelets and reduced the risk of cGVHD incidence. Similar effects were also observed in the subgroup in which MSCs were infused after HSCT, as reported in a previous meta-analysis [54]. Although no significant beneficial effects of MSCs were observed in the subgroup in which MSCs were infused before HSCT [54], this is probably because both of the included studies were performed in patients with hematological malignancies along with the limited statistical effect caused by the small number of studies and sample sizes. Regarding the duration of the MSC infusion, some investigators have suggested that the infusion should last for 30 min or more [24, 34]. Another study reported that MSCs were infused at a rate of 5 mL/min [31]. Compared with fresh MSCs, MSCs subjected to freezing and thawing display increased activation of the instant blood-mediated inflammatory reaction and susceptibility to complement-mediated lysis, exerting smaller immunomodulatory effects [63, 70]. Thus, upon systemic infusion, fresh MSCs are more effective in the clinic than freeze-thawed MSCs [63].

Another important safety concern in MSC therapy is their procoagulant effects after blood contact, as suggested by a large number of in vitro studies [63]. Therefore, we must highlight the importance of monitoring thrombotic events after the MSC infusion, especially in patients who have acquired hypercoagulability or are at high risk of thrombosis due to their primary disease process [63]. In a word, one size does not fit all. The use of MSCs and the precise regimens of MSCs used in allo-HSCT depend on the specific clinical condition of patients. Further studies of MSC science and their therapeutic potential are needed. We conducted this study based on the assumption that MSC co-transplantation in allo-HSCT would be a highly effective treatment approach and/or an early therapeutic intervention with clear prognostic benefits for patients with hematological diseases, particularly hematological malignancies. In these cases, the inclusion of genetic information such as single nucleotide polymorphisms into the prediction and diagnosis of hematological malignancies may be of high clinical value. In addition to predicting the occurrence of such diseases and guiding the judgment of curative effect after treatment, the application of genetic data may also rapidly improve the accuracy of diagnosis and reduce the number of patients diagnosed with false negative [71].

Several limitations exist in this study. First, heterogeneity across studies, including the types and stages of diseases, sources and dosage of HSCs and MSCs, HLA matching, definitions of outcomes, varying follow-up times, and study designs, was notable, although we performed subgroup and sensitivity analyses to attempt to resolve the heterogeneity. Additionally, in the subgroups of non-malignant disorders and HLA-identical matching, no more than 3 trials were included in each meta-analysis. Second, publication bias was possible because of the 4 trials (ChiCTR-OCN-15006595, ChiCTR-IIR-16007806, NCT01092026; NCT00081055) [72–75], which were completed but we failed to find any related publications. Third, we restricted the language of the literature search to only Chinese and English, which may result in a failure to retrieve some potentially relevant studies. Fourth, 3 trials [27, 29, 39], which were published in the form of abstracts in poster sessions, did not provide us with sufficient information to fully assess the source of the bias. Last, the limitations of the *I*^2^ test must be considered. Although most of the *I*^2^ test scores were less than 50%, the *I*^2^ values are likely to be underestimated, particularly if a limited number of trials or a few events are assessed in a meta-analysis [76, 77].

## Conclusion

Collectively, the treatment strategy of an MSC co-infusion with allo-HSCT generally improved engraftment and reduced the risk of developing cGVHD, without increasing the risk of mortality or relapse. In terms of aGVHD and NRM, the effect of the MSC co-infusion was not quite significant. Specifically, the data obtained support the application of MSCs co-transplanted with HLA-nonidentical HSCT in children and young individuals. Since the effects of MSCs on blood malignancies are not well understood, we do not recommend the use of MSCs co-transplanted with HLA-identical HSCT in adult patients with hematological malignancies based on the currently available data. In this meta-analysis, the number of studies conducted on patients with hematological malignancies undergoing HLA-identical HSCT is limited. Hence, additional in-depth assessments of the safety and efficacy of MSC co-transplantation in this population are recommended to avoid harming patients.

## Supplementary Information

**Additional file 1: Table S1**. The Eligibility Criteria for included studies in systematic review.

**Additional file 2: Text S1**. Detailed search strategies.

**Additional file 3: Fig. S1**. Funnel plots of publication bias evaluating the effect of MSC co-infusion on (a) neutrophil engraftment and (b) platelet engraftment.

**Additional file 4: Fig. S2**. Subset analysis based on the dosage of MSCs for outcomes of neutrophil engraftment (a&b) and platelet engraftment (c&d).

**Additional file 5: Fig. S3**. Funnel plots of publication bias evaluating the effect of MSC co-infusion on (a) aGVHD and (b) cGVHD.

**Additional file 6: Fig. S4**. Funnel plots of publication bias evaluating the effect of MSC co-infusion on (a) OS and (b) RR.

**Additional file 7: Fig. S5**. Assessment of neutrophil engraftment in subgroup analysis according to (a) type of disease, (b) HLA matching and (c) average age.

**Additional file 8: Fig. S6**. Assessment of platelet engraftment in subgroup analysis according to (a) type of disease, (b) HLA matching and (c) average age.

**Additional file 9: Fig. S7**. Assessment of aGVHD in subgroup analysis according to (a) type of disease, (b) HLA matching and (c) average age.

**Additional file 10: Fig. S8**. Assessment of cGVHD in subgroup analysis according to (a) type of disease, (b) HLA matching and (c) average age.

**Additional file 11: Fig. S9**. Assessment of OS in subgroup analysis according to (a) type of disease, (b) HLA matching and (c) average age.

**Additional file 12: Fig. S10**. Assessment of RR in subgroup analysis according to (a) type of disease, (b) HLA matching and (c) average age.

**Additional file 13: Fig. S11**. Assessment of NRM in subgroup analysis according to (a) type of disease, (b) HLA matching and (c) average age.

## Data Availability

All supporting data and materials were included in the article and its additional files.

## Abbreviations

allo-HSCT: Allogeneic hematopoietic stem cell transplantation
HSCs: Hematopoietic stem cells
MSCs: Mesenchymal stem cells
PRISMA: Preferred Reporting Items for Systematic Reviews and Meta-Analysis
HLA: Human leukocyte antigen
GVL: Graft-versus-leukemia
BM: Bone marrow
UCB: Umbilical cord blood
SAA: Severe aplastic anemia
PB: Peripheral blood
aGVHD/cGVHD: Acute/chronic graft-versus-host disease
OS: Overall survival
RR: Relapse rate
NRM: Non-relapse mortality
RCTs: Randomized controlled trials
nRCTs: Non-randomized controlled trials
RoB: Risk of bias
SMD: Standardized mean difference
RR: Risk ratio
OR: Odds ratios
HR: Hazard ratio
SD: Standard deviation
CIs: Confidence intervals
ANC: Absolute neutrophil count
PLT: Platelet
DCs: Dendritic cells
NK cells: Natural killer cells
IV: Intravenous
IA: Intraarterial
