# Supplementary material for: Efficacy and safety of mesenchymal stem cells co-infusion in allogeneic hematopoietic stem cell transplantation: a systematic review and meta-analysis
Source: Stem Cell Res Ther. 2021 Apr 20;12:246. doi: 10.1186/s13287-021-02304-x (PMC8056684; doi:10.1186/s13287-021-02304-x)
Supplement: Supplementary file 1 — Additional file 1: Table S1. The Eligibility Criteria for included studies in systematic review. [file 13287_2021_2304_MOESM1_ESM.doc]

**Table S1. The Eligibility Criteria for included studies in systematic review**

| Criteria | Included | Excluded |
| --- | --- | --- |
| Population | Patients enrolled of all age who were diagnosed with hematological disease, and were in need of an allo-HSCT. | N/A |
| Intervention | Co-infusion of MSCs with HSCs, both of which were administrated within day ‘0’ whenever allowed by the patient’s condition, otherwise within the coming 24 h. All sources of stem cells as well as different donor relations (related and unrelated, matched and unmatched), of both MSCs and HSCs, could be applied. | Whereas studies using MSCs in other settings or in combination with other experimental cells and/or treatments were excluded. |
| Comparison | Receiving allo-HSCT alone, alternatively allo-HSCT plus placebo. | N/A |
| Outcomes | Articles were included if they mentioned to any of the following outcomes: (I) Engraftment, (II) Graft versus host disease (GVHD), (III) Relapse rate (RR), (IV) Overall survival (OS), (V) Treatment related mortality or non-relapse mortality (TRM/NRM), (VI) lymphocyte recovery. | N/A |
| Design | Randomized controlled trials or  non-randomized controlled trials (RCTs or  nRCTs respectively) | All other |
| Language | studies published in English or Chinese. | N/A |
