# Supplementary material for: Efficacy and safety of mesenchymal stem cells co-infusion in allogeneic hematopoietic stem cell transplantation: a systematic review and meta-analysis
Source: Stem Cell Res Ther. 2021 Apr 20;12:246. doi: 10.1186/s13287-021-02304-x (PMC8056684; doi:10.1186/s13287-021-02304-x)
Supplement: Supplementary file 2 — Additional file 2: Text S1. Detailed search strategies. [file 13287_2021_2304_MOESM2_ESM.doc]

**Text S1. Detail Search strategies**

**Part 1.** PubMed search strategy

**Part 2.** Web of Science search strategy

**Part 3.** Embase search strategy

**Part 4.** Cochrane Library search strategy

**Part 5.** SinoMed search strategy

**Part 6.** ClinicalTrials. gov search strategy

**Part 1.** PubMed search strategy

#1 (“Bone Marrow Transplantation”[Mesh]) OR (((((Grafting, Bone Marrow[Title/Abstract]) OR Bone Marrow Grafting[Title/Abstract]) OR Transplantation, Bone Marrow[Title/Abstract]) OR Bone Marrow Cell Transplantation[Title/Abstract]) OR Transplantation, Bone Marrow Cell[Title/Abstract]) 44898

#2 (((Stem Cell Transplantation, Hematopoietic[Title/Abstract]) OR Transplantation, Hematopoietic Stem Cell[Title/Abstract])) OR “Hematopoietic Stem Cell Transplantation”[Mesh] 44677

#3 #1 OR #2

#4 (“Mesenchymal Stem Cells”[Mesh]) OR (((((((Stem Cell*, Mesenchyma[Title/Abstract]) OR Mesenchymal Stromal Cell*[Title/Abstract]) OR Stromal Cell*, Mesenchymal[Title/Abstract]) OR Mesenchymal Progenitor Cell*[Title/Abstract]) OR

Progenitor Cell*, Mesenchyma[Title/Abstract]) OR Bone Marrow Stromal*[Title/Abstract]) OR Wharton* Jelly Cell*[Title/Abstract]) 49773

#5 #3 AND #4 1428

#6 #5 limit to human 1262

#7 #6 limit to Clinical trial or RCT 63

**Part 2.** Web of Science search strategy

#1 TOPIC: (“bone marrow transplantation”) OR TOPIC: (“Grafting, Bone Marrow”) OR TOPIC: (“bone marrow grafting”) OR TOPIC: (“transplantation, bone marrow”) OR TOPIC: (“bone marrow cell transplantation”) OR TOPIC: (“transplantation, bone marrow cell”) 154430

#2 TOPIC: (“Hematopoietic Stem Cell Transplantation”) OR TOPIC: (“Stem Cell Transplantation, Hematopoietic”) OR TOPIC: (“Transplantation, Hematopoietic Stem Cell”) 97501

#3 TOPIC: (“Mesenchymal Stem Cell*”) OR TOPIC: (Stem Cell*,Mesenchymal) OR TOPIC: (Mesenchymal Stromal Cell*) OR TOPIC: ( Stromal Cell*, Mesenchymal) OR TOPIC: (Mesenchymal Progenitor Cell*)OR TOPIC: (Progenitor Cell*, Mesenchymal) OR TOPIC: (Bone Marrow Stromal*) OR TOPIC: ( Wharton* Jelly Cell*) 143099

#4 #1 OR #2

#5 #3 AND #4 25241

#6 TOPIC:  (hematological disease) OR TOPIC:  (hematopathy) OR TOPIC:  (hematological disorder) OR TOPIC:  (hemic and lymphatic diseases) OR TOPIC:  (hematologic disease*) OR TOPIC: (hematologic neoplasms) OR TOPIC:  (hemopathy) OR TOPIC:  (blood disorder) OR TOPIC:  (haematologic disease*) OR TOPIC:  (haematologic neoplasms) OR TOPIC:  (haematological dis*) OR TOPIC:  (blood disease)

Databases= WOS, BIOSIS, KJD, MEDLINE, RSCI, SCIELO Timespan=All years

Search language=Auto

#7 #5 AND #6 11005

#8 limit to human 825

#9 limit to clinical trial or RCT 199

**Part 3.** Embase search strategy

#1 'bone marrow transplantation'/exp/mj OR 'bone marrow transplantation':ab,ti OR 'bone marrow cell transplantation':ab,ti OR 'transplantation, bone marrow':ab,ti OR 'bone marrow grafting':ab,ti OR 'grafting, bone marrow':ab,ti 48524

#2 'hematopoietic stem cell transplantation'/exp/mj OR 'stem cell transplantation, hematopoietic':ab,ti OR 'transplantation, hematopoietic stem cell':ab,ti 34075

#3 #1 AND #2 1856

#4 'mesenchymal stem cells'/exp/mj OR 'wharton* jelly cell*':ab,ti OR 'bone marrow stromal*':ab,ti OR 'progenitor cell*, mesenchymal':ab,ti OR 'mesenchymal progenitor cell*':ab,ti OR 'mesenchymal stromal cell*':ab,ti OR 'stem cell*,mesenchymal':ab,ti OR 'stromal cell*, mesenchymal':ab,ti 52452

#5 #3 AND #4 21

**Part 4.** Cochrane Library search strategy

#1 (bone marrow transplantation):ti,ab,kw 4563

#2 (Grafting, Bone Marrow):ti,ab,kw 121

#3 (Bone Marrow Grafting):ti,ab,kw 121

#4 (Transplantation, Bone Marrow Cell):ti,ab,kw 2754

#5 (Bone Marrow Cell Transplantation):ti,ab,kw 2754

#6 (Transplantation,Bone Marrow):ti,ab,kw 193

#7 #1OR#2OR#3OR#4OR#5OR#6 (4610)

#8 (Transplantation, Hematopoietic Stem Cell):ti,ab,kw 3526

#9 (hematopoietic stem cell transplantation):ti,ab,kw 3526

#10 (Transplantation, Hematopoietic Stem Cell):ti,ab,kw 3526

#11 #8OR#9OR#10 3526

#12 #7OR#11 7199

#13 (Mesenchymal Stem Cells):ti,ab,kw 1262

#14 (Stem Cell*,Mesenchymal):ti,ab,kw 0

#15 (Mesenchymal Stromal Cell*):ti,ab,kw 296

#16 (Stromal Cell*, Mesenchymal):ti,ab,kw 0

#17 (Mesenchymal Progenitor Cell*):ti,ab,kw 67

#18 (Progenitor Cell*, Mesenchymal):ti,ab,kw 0

#19 (Bone Marrow Stromal*):ti,ab,kw 233

#20 (Wharton* Jelly Cell*):ti,ab,kw 33

#21 #13OR#14OR#15OR#16OR#17OR#18OR#19OR#20 1474

#22 #21AND#12 368

-2 reviews 366

**Part 5.** SinoMed search strategy

#1 (("骨髓移植"[摘要:智能] OR "骨髓净化"[摘要:智能] OR "造血干细胞动员"[摘要:智能] OR "造血干细胞移植"[摘要:智能])

#2 ("间充质基质细胞"[摘要:智能] OR "间质干细胞"[摘要:智能] OR "间充质"[摘要:智能] OR "间质细胞"[摘要:智能] OR "间充质干细胞"[摘要:智能]))

#3 #1 AND #2

#4 #3 limit to ("循证文献"[文献类型] OR "临床试验"[文献类型] OR "病例报告"[文献类型] OR "随机对照试验"[文献类型]) 91

**Part 6.** ClinicalTrials. gov search strategy

#1 “Bone Marrow Transplantation” OR “Grafting, Bone Marrow” OR “Bone Marrow Grafting” OR “Transplantation, Bone Marrow” OR “Bone Marrow Cell Transplantation” OR “Transplantation, Bone Marrow Cell” OR “Stem Cell Transplantation, Hematopoietic” OR “Transplantation, Hematopoietic Stem Cell” OR “Hematopoietic Stem Cell Transplantation”

#2 “Mesenchymal Stem Cells” OR “Stem Cell*,Mesenchymal”OR “Mesenchymal Stromal Cell*”OR “Stromal Cell*, Mesenchymal”OR “Mesenchymal Progenitor Cell*” OR “Progenitor Cell*, Mesenchymal” OR “Bone Marrow Stromal*” OR “Wharton* Jelly Cell*”

#3 #1AND #2 212
