# Supplementary figures and images for: Efficacy and safety of mesenchymal stem cells co-infusion in allogeneic hematopoietic stem cell transplantation: a systematic review and meta-analysis
Source: Stem Cell Res Ther. 2021 Apr 20;12:246. doi: 10.1186/s13287-021-02304-x (PMC8056684; doi:10.1186/s13287-021-02304-x)

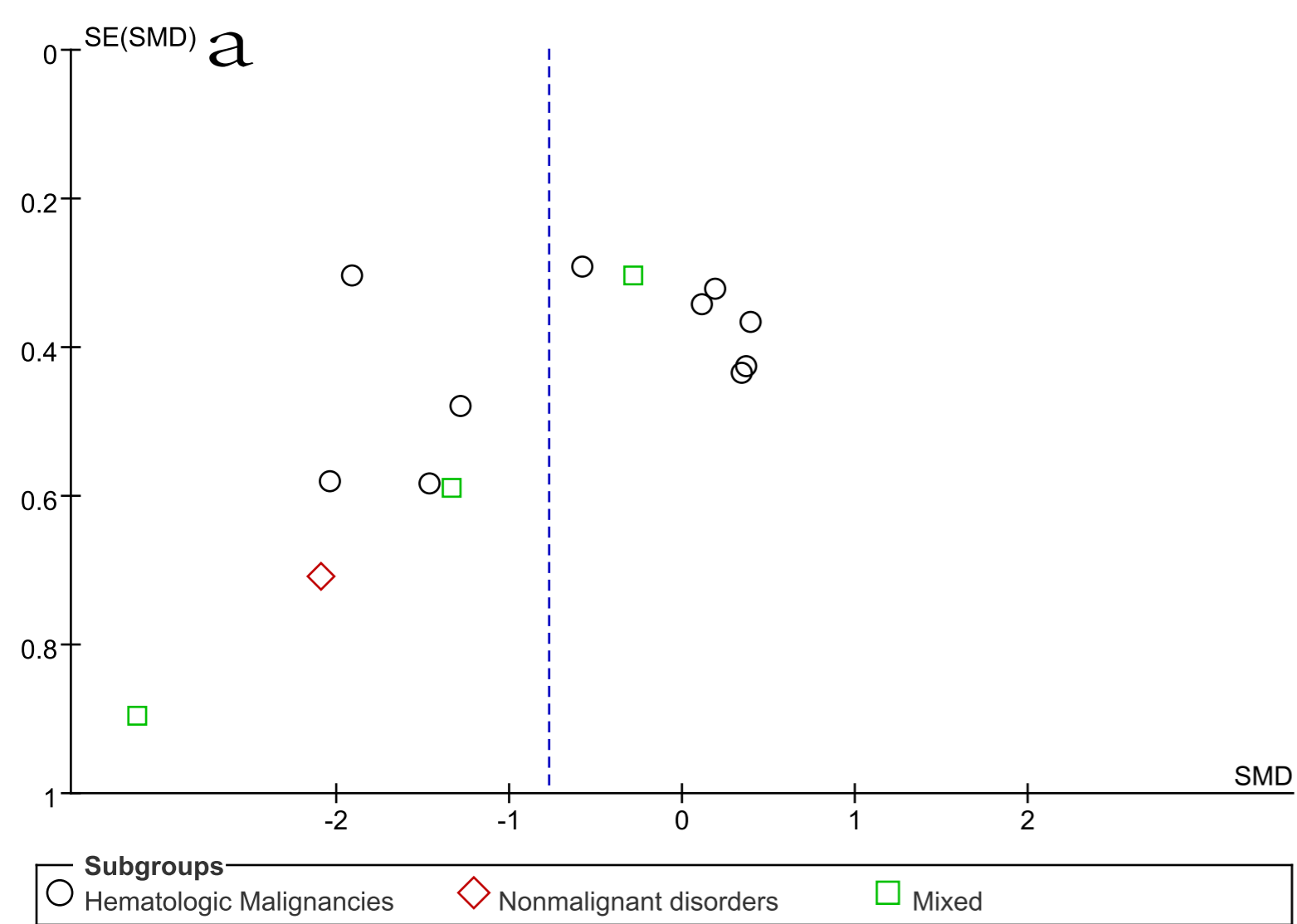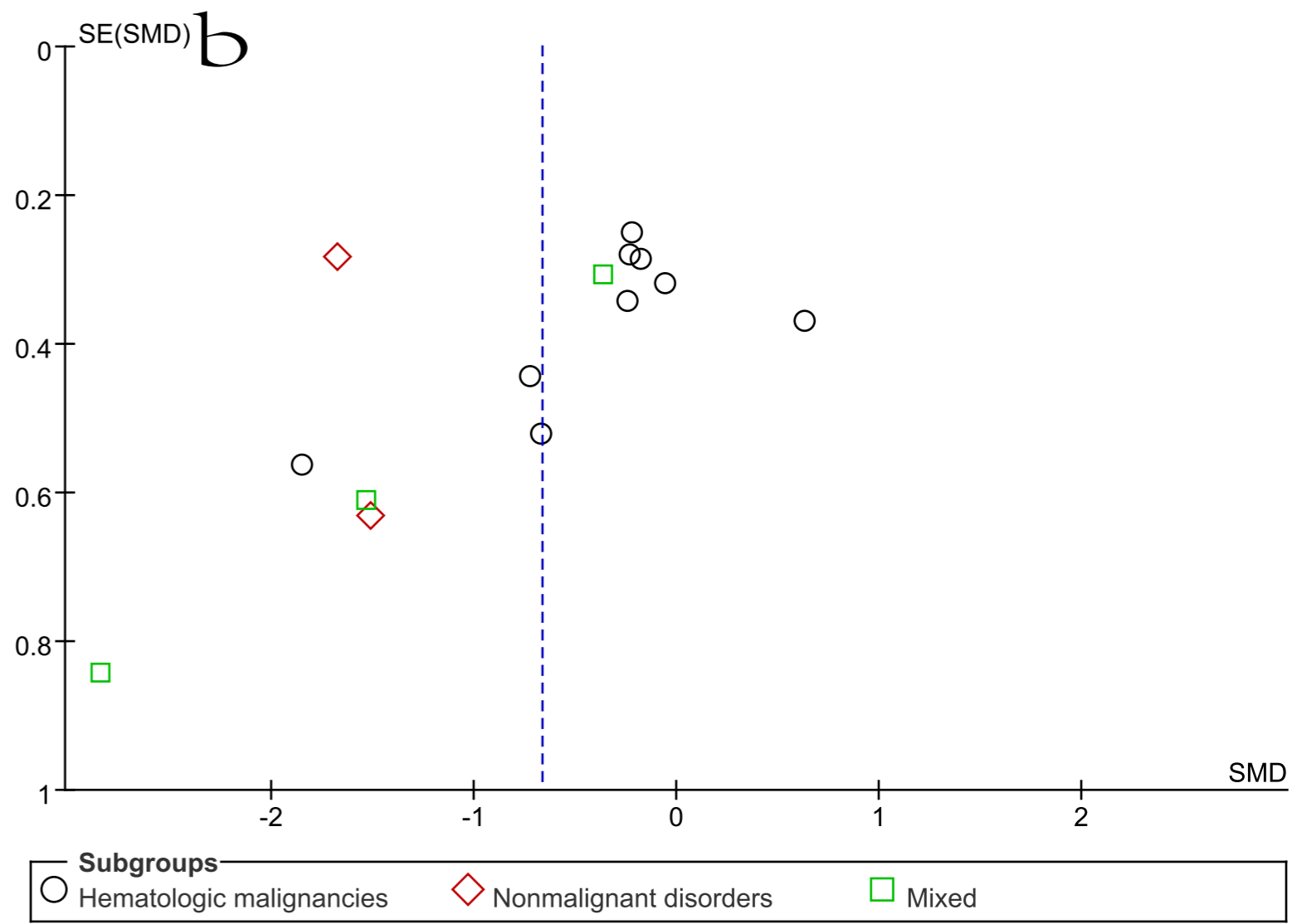

Supplement: Supplementary file 3 — Additional file 3: Fig. S1. Funnel plots of publication bias evaluating the effect of MSC co-infusion on (a) neutrophil engraftment and (b) platelet engraftment. [file 13287_2021_2304_MOESM3_ESM.pdf]

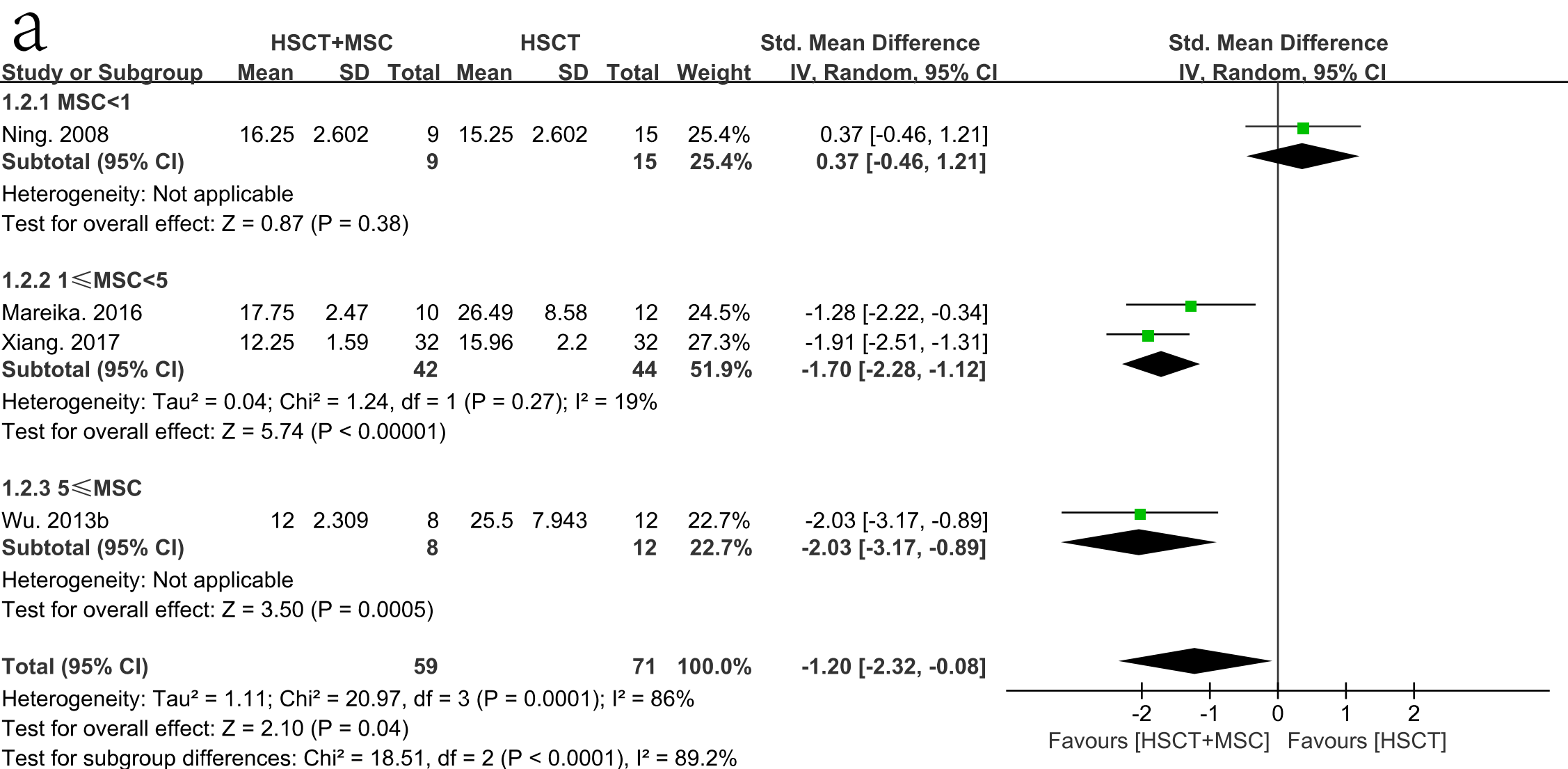

Supplement: Supplementary file 4 — Additional file 4: Fig. S2. Subset analysis based on the dosage of MSCs for outcomes of neutrophil engraftment (a&b) and platelet engraftment (c&d). [file 13287_2021_2304_MOESM4_ESM.pdf]

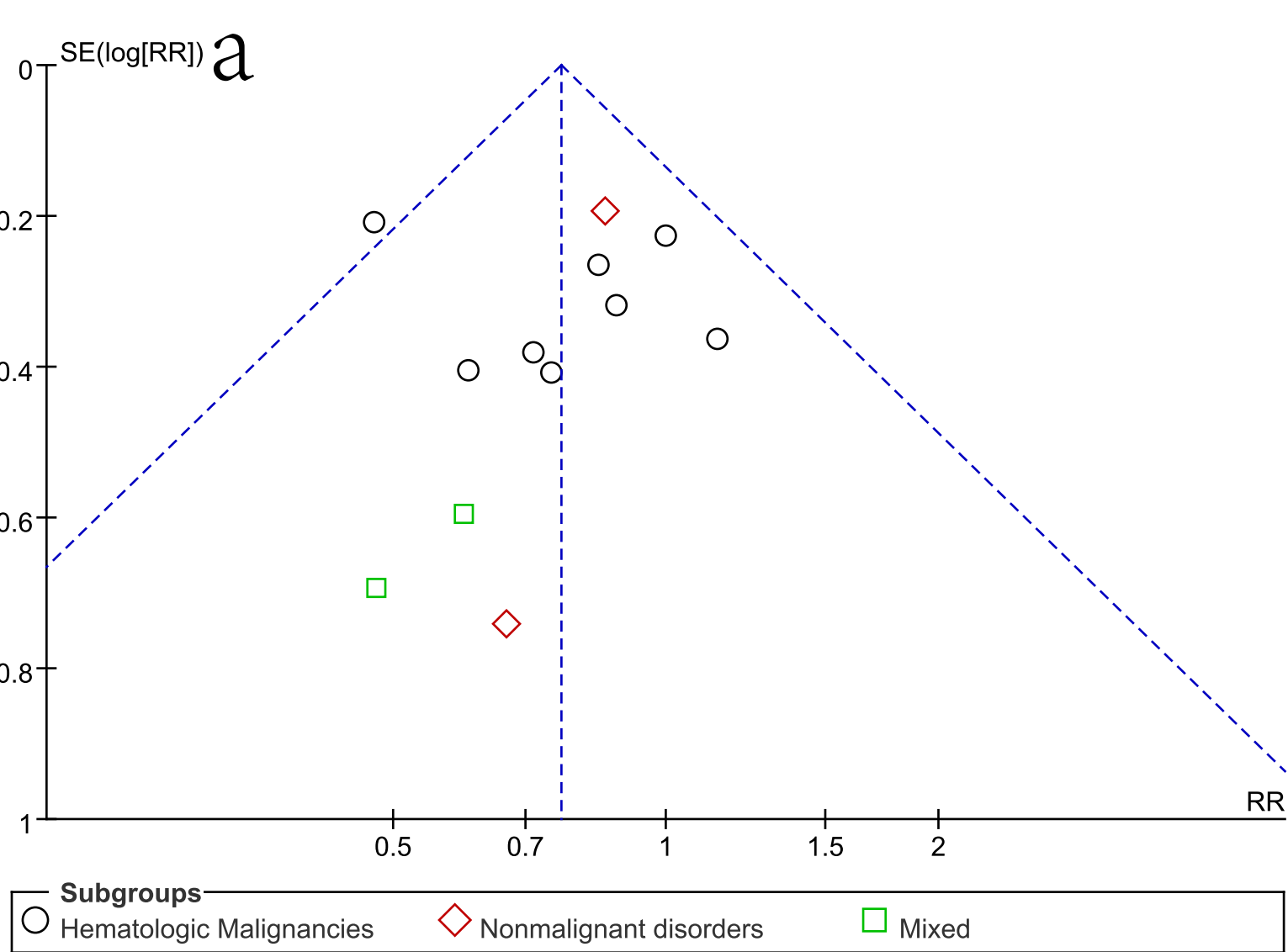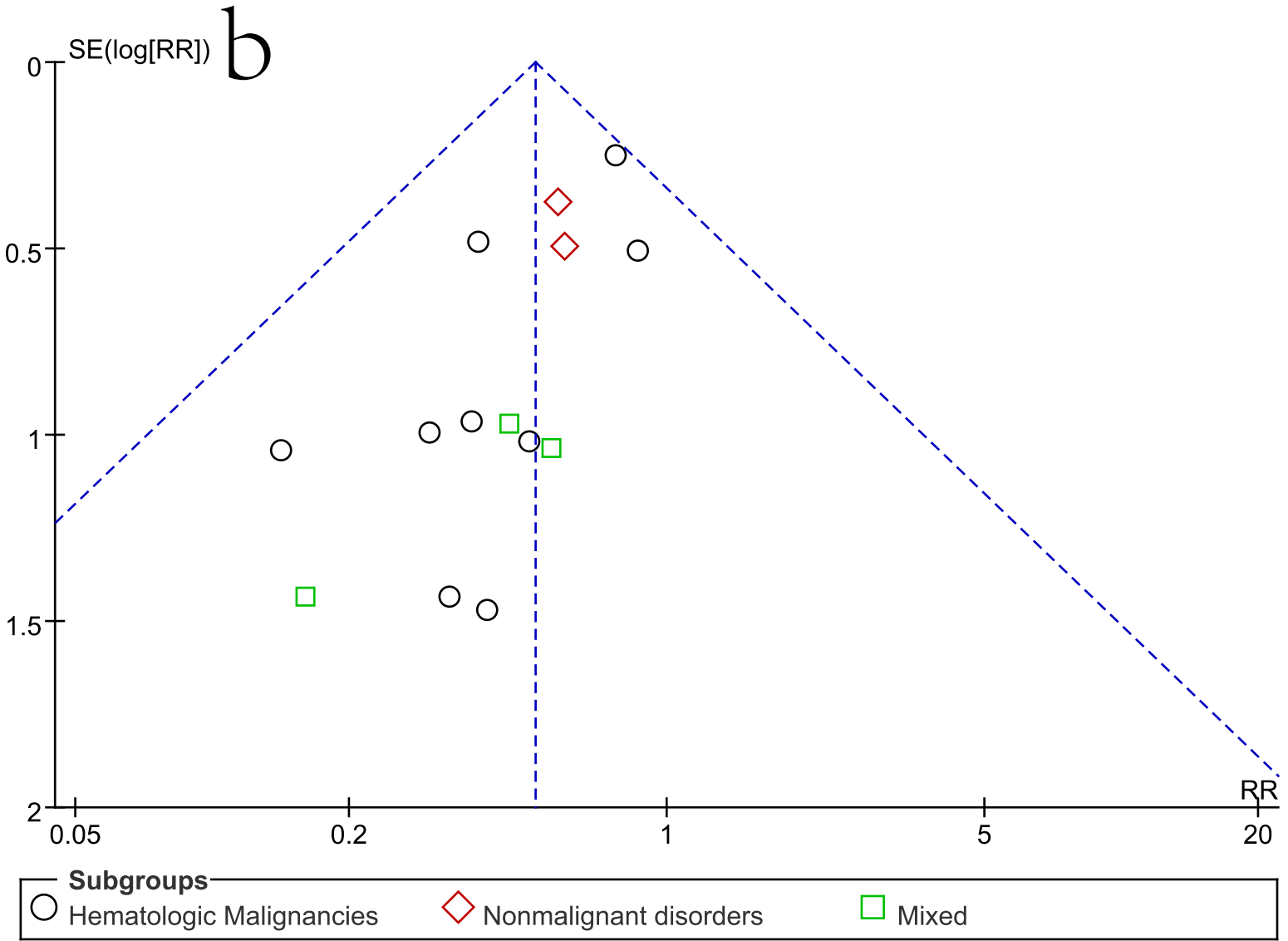

Supplement: Supplementary file 5 — Additional file 5: Fig. S3. Funnel plots of publication bias evaluating the effect of MSC co-infusion on (a) aGVHD and (b) cGVHD. [file 13287_2021_2304_MOESM5_ESM.pdf]

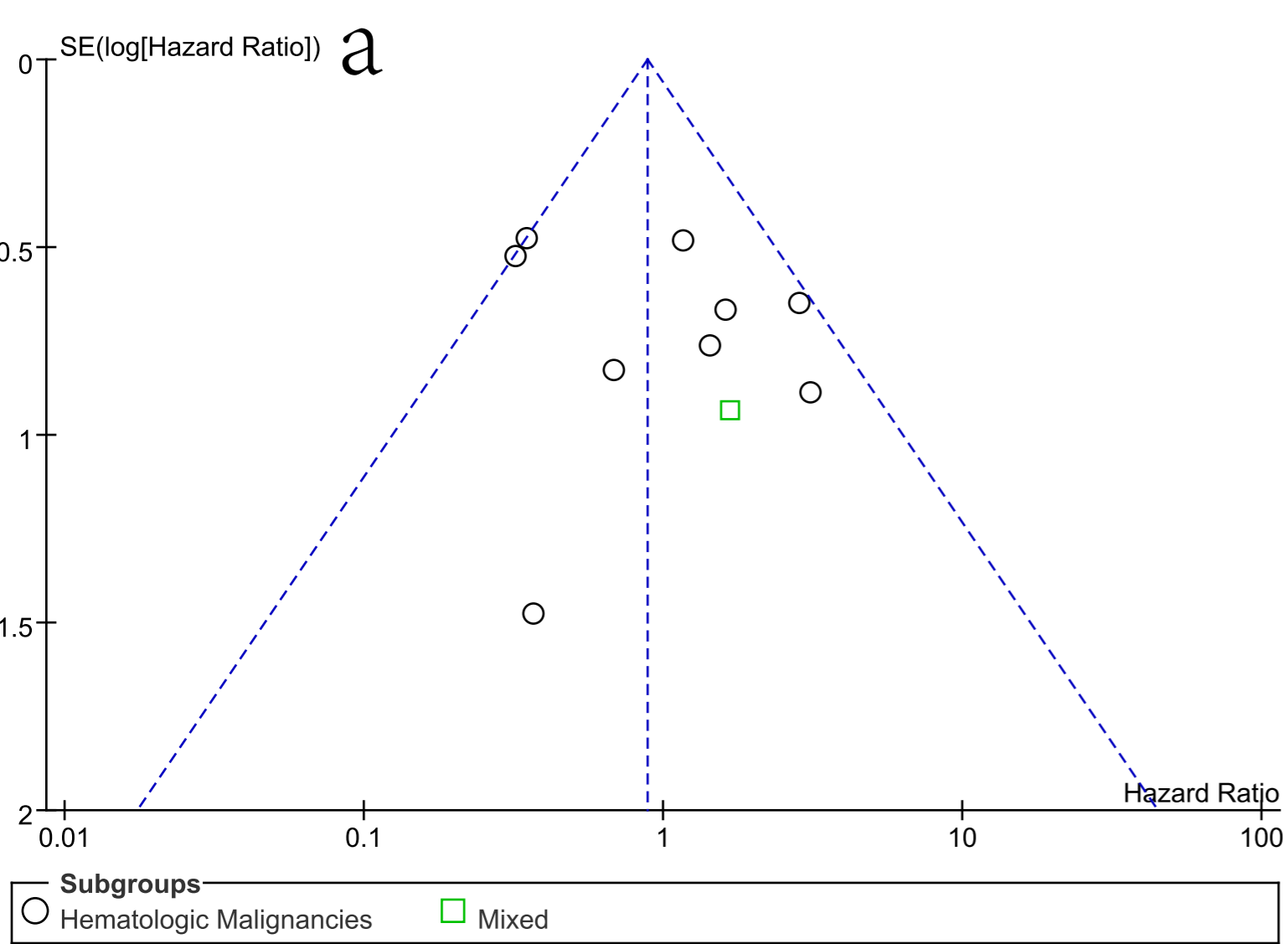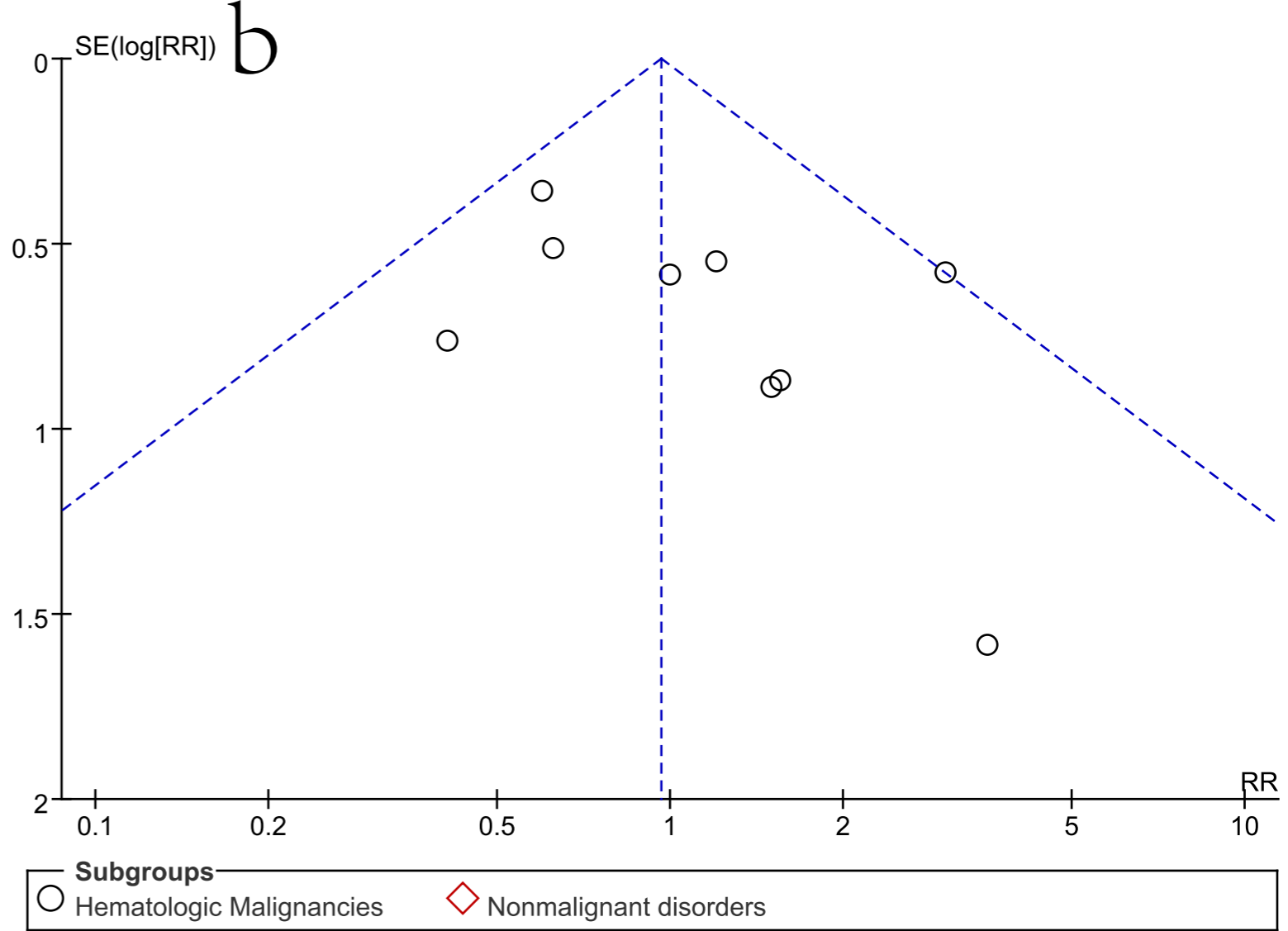

Supplement: Supplementary file 6 — Additional file 6: Fig. S4. Funnel plots of publication bias evaluating the effect of MSC co-infusion on (a) OS and (b) RR. [file 13287_2021_2304_MOESM6_ESM.pdf]

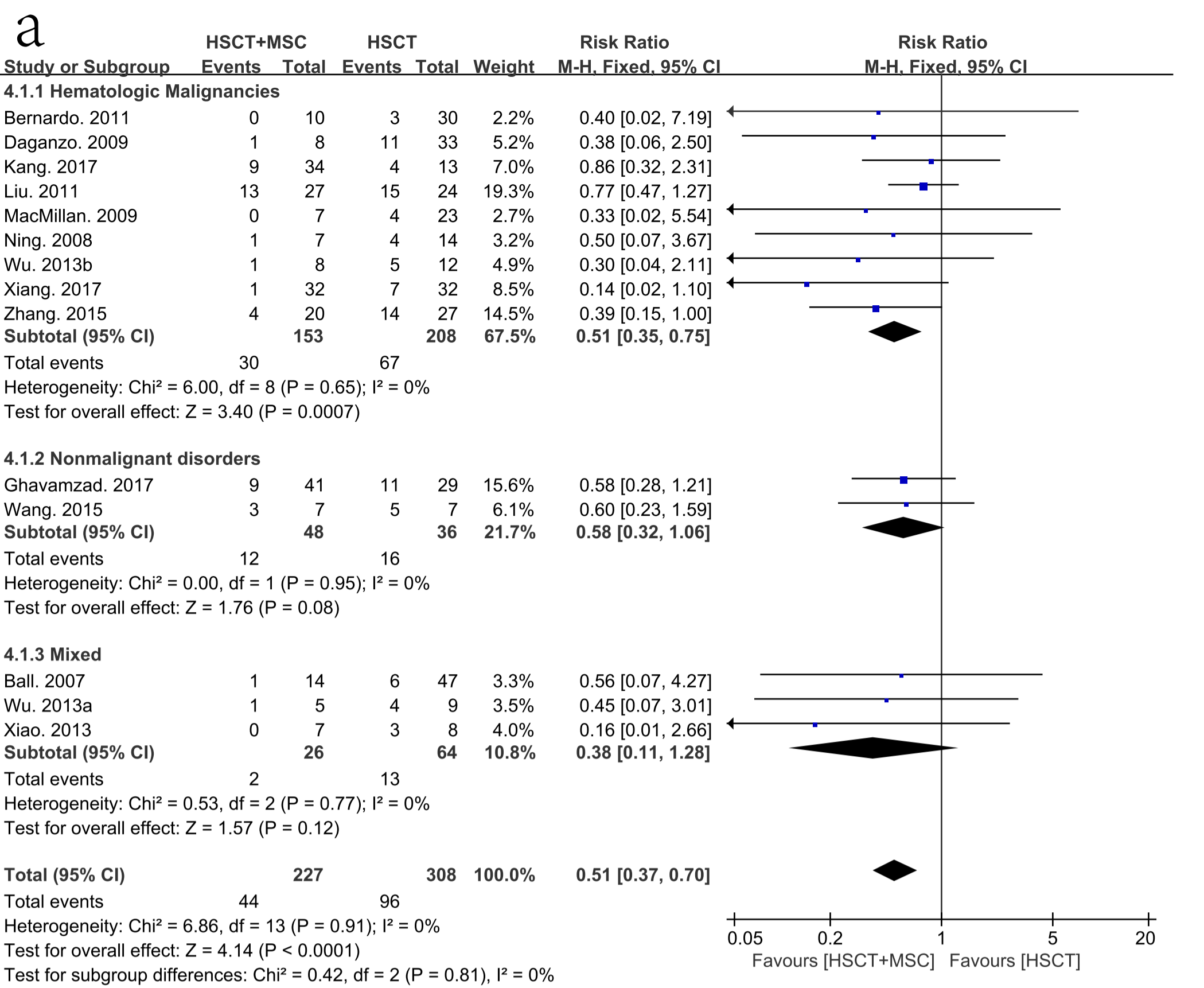

Supplement: Supplementary file 10 — Additional file 10: Fig. S8. Assessment of cGVHD in subgroup analysis according to (a) type of disease, (b) HLA matching and (c) average age. [file 13287_2021_2304_MOESM10_ESM.pdf]

a

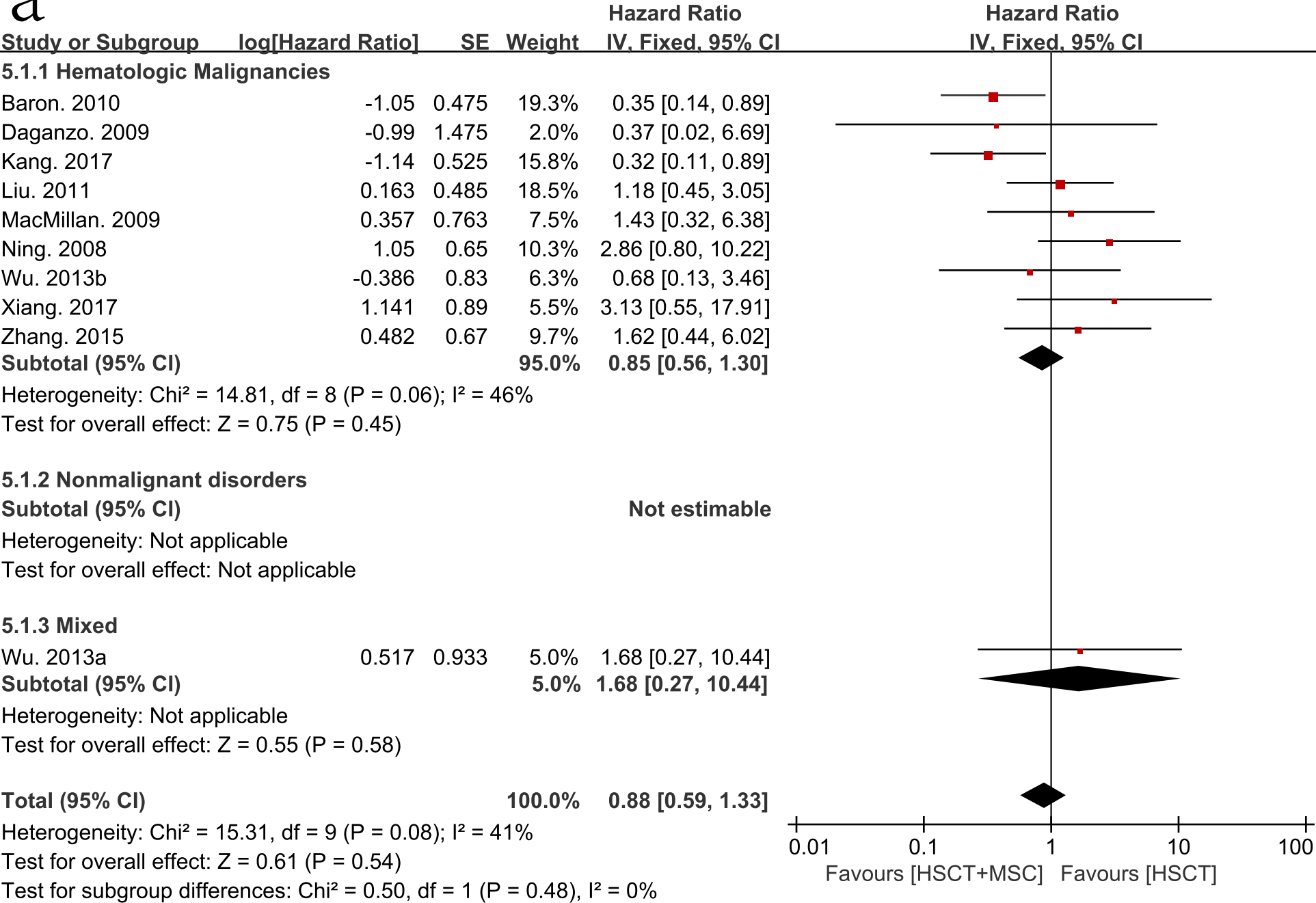

b

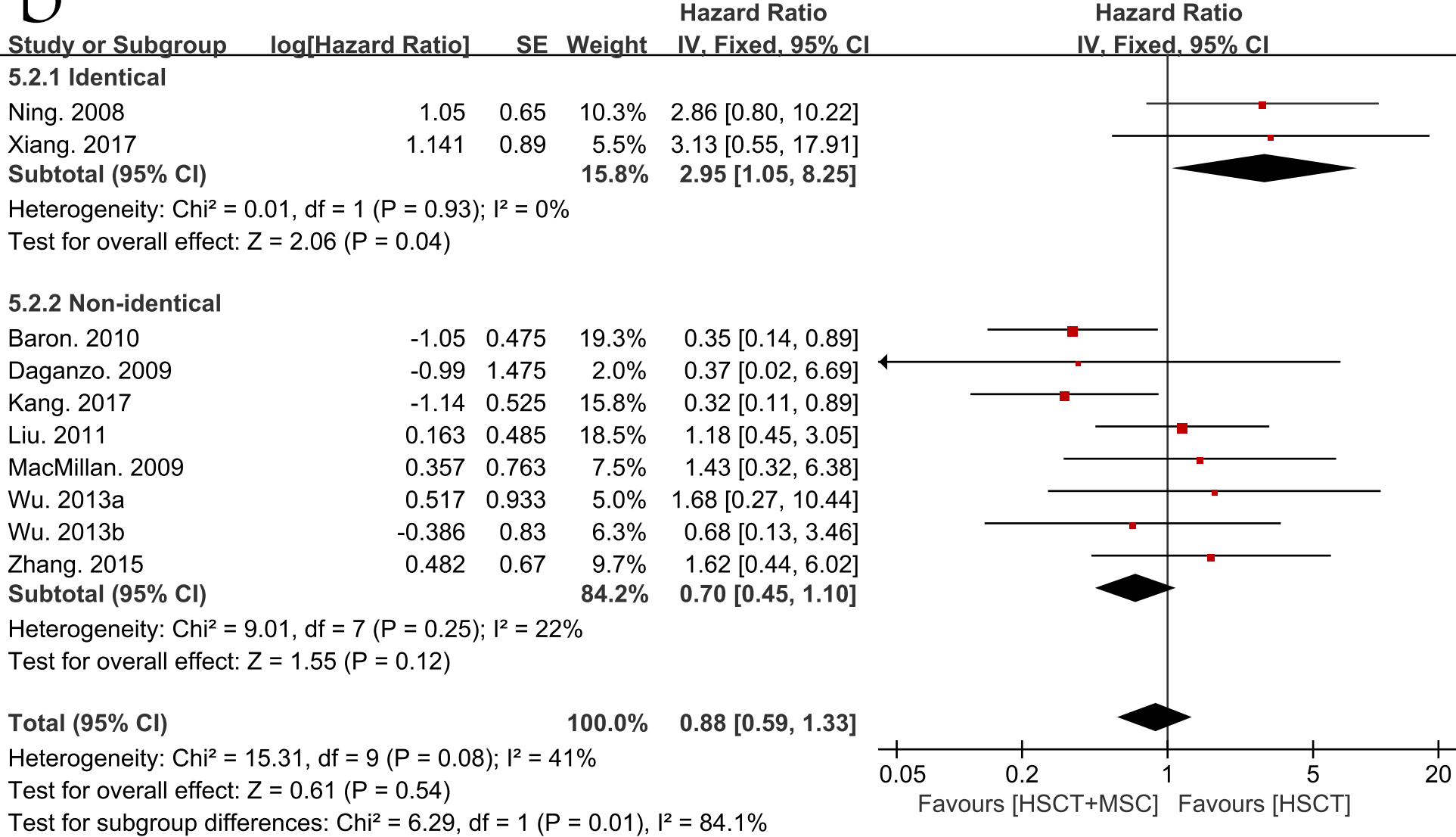

c

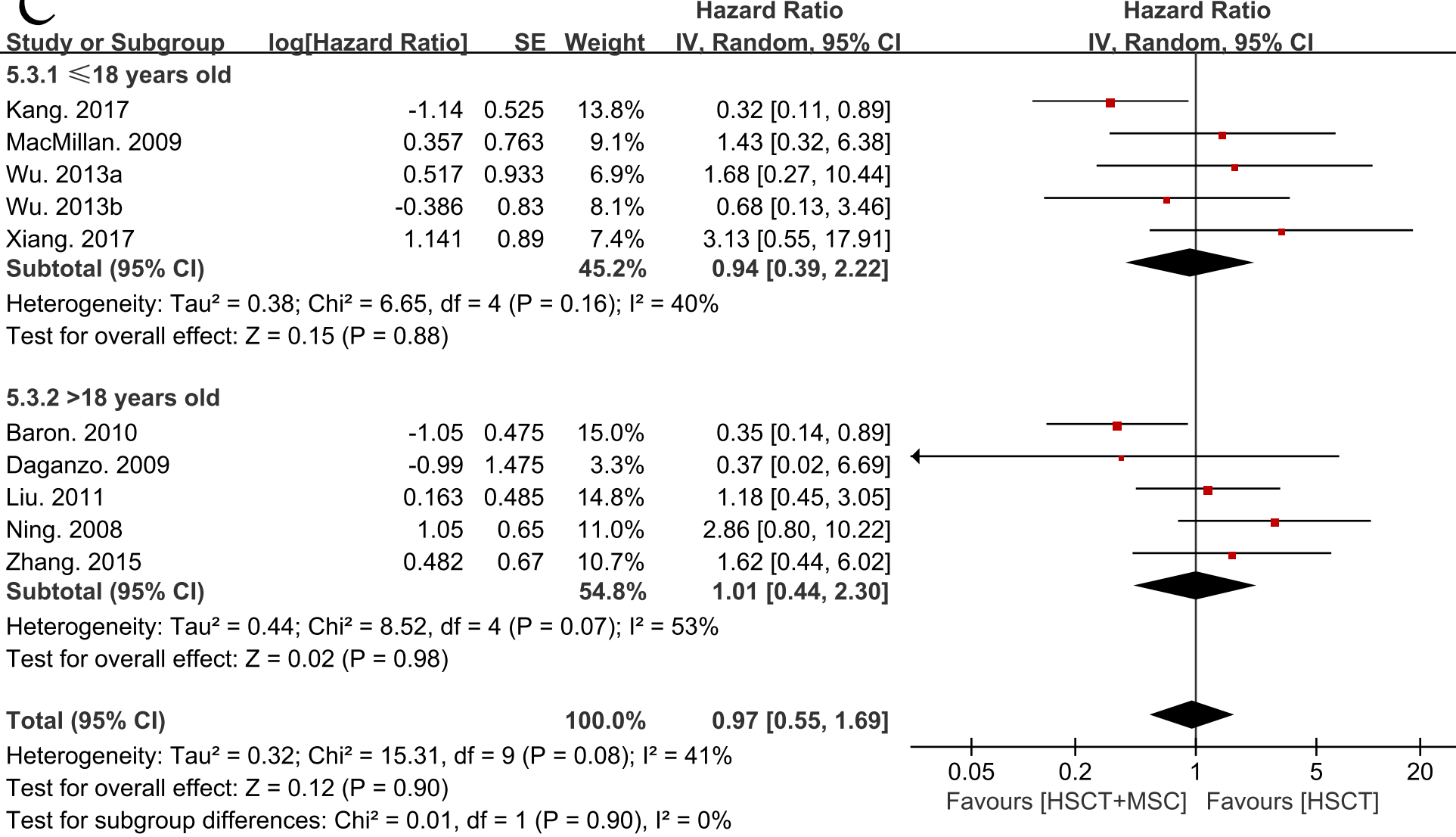

Supplement: Supplementary file 11 — Additional file 11: Fig. S9. Assessment of OS in subgroup analysis according to (a) type of disease, (b) HLA matching and (c) average age. [file 13287_2021_2304_MOESM11_ESM.pdf]

a

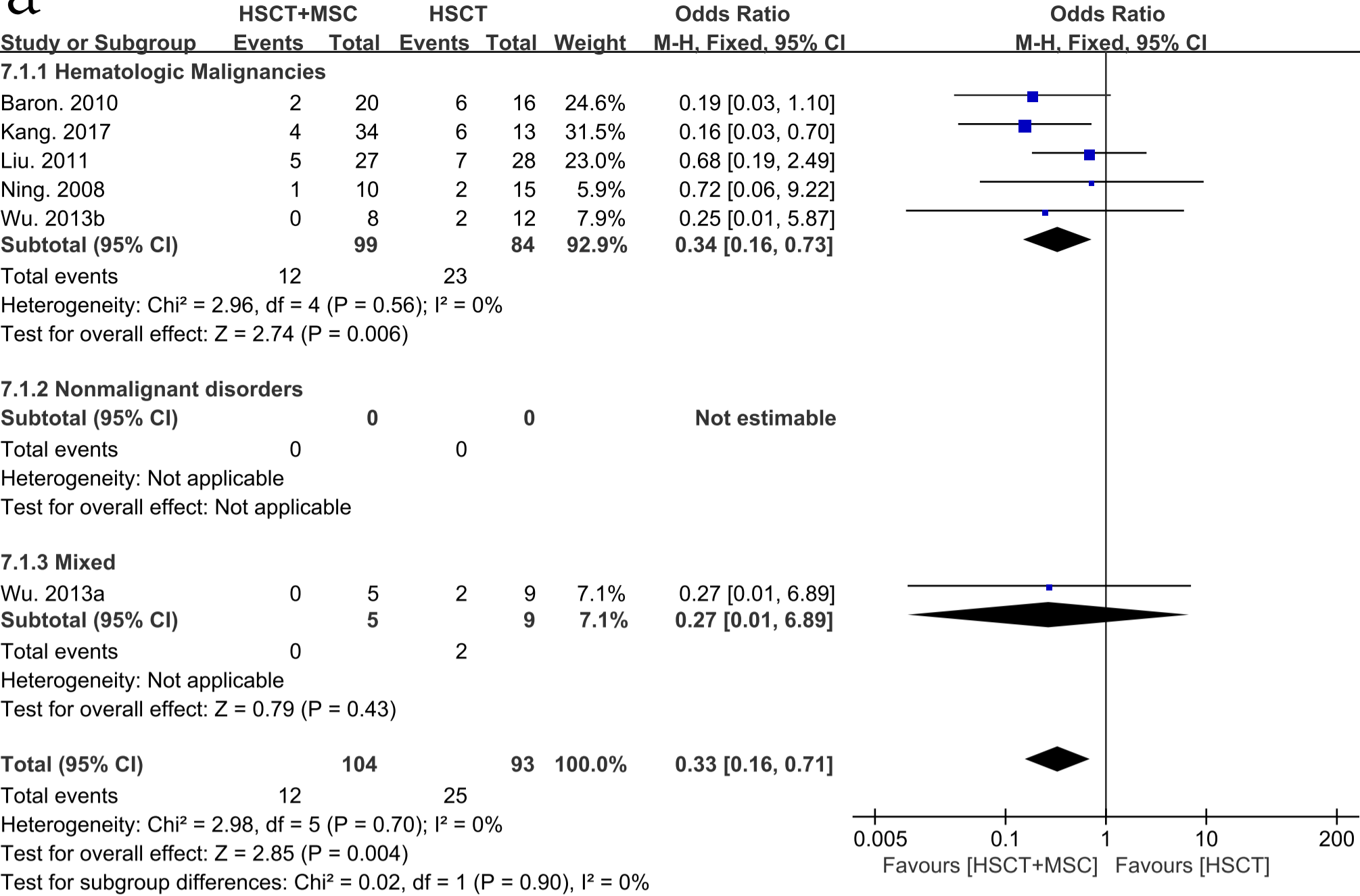

b

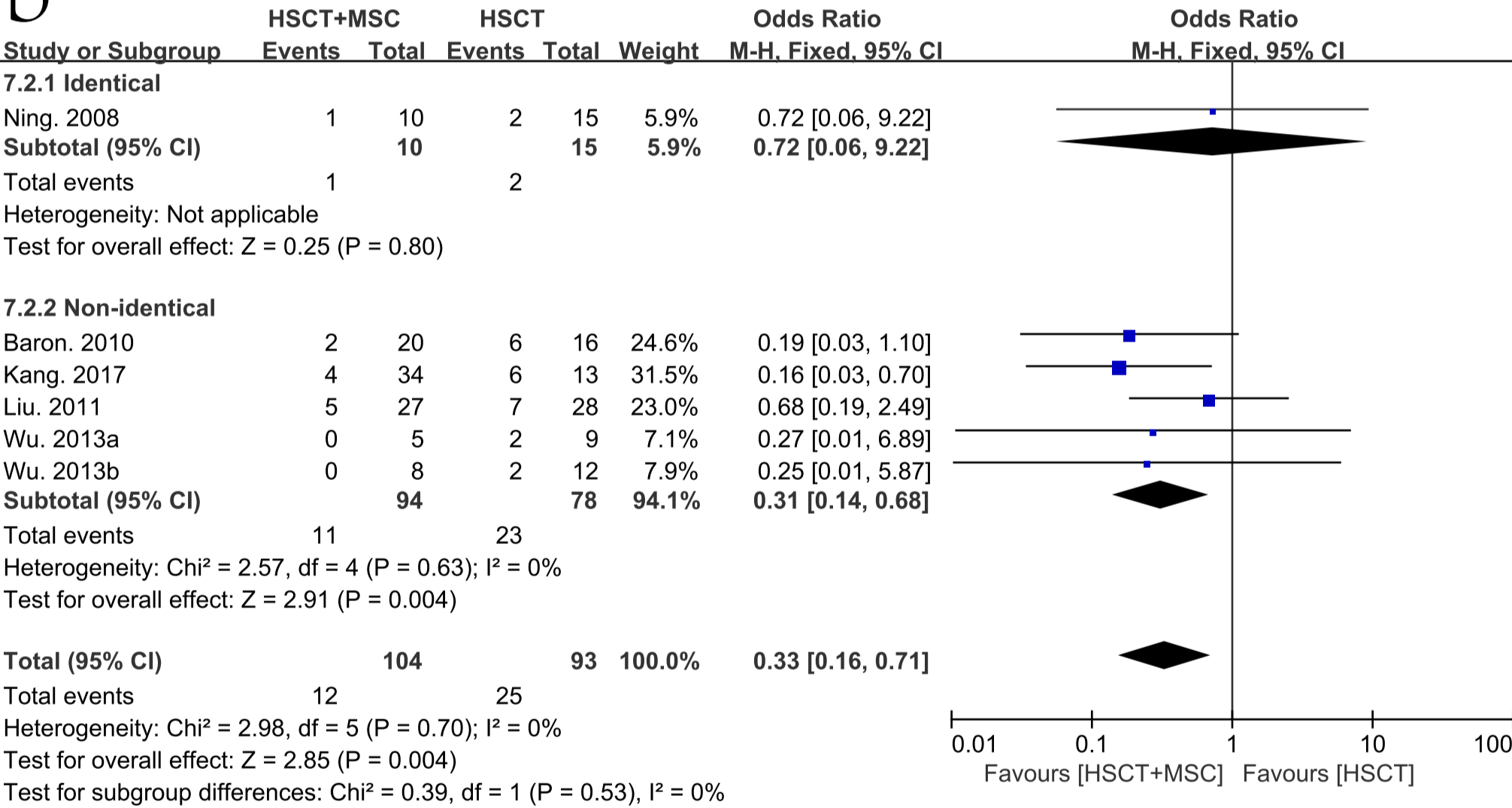

c

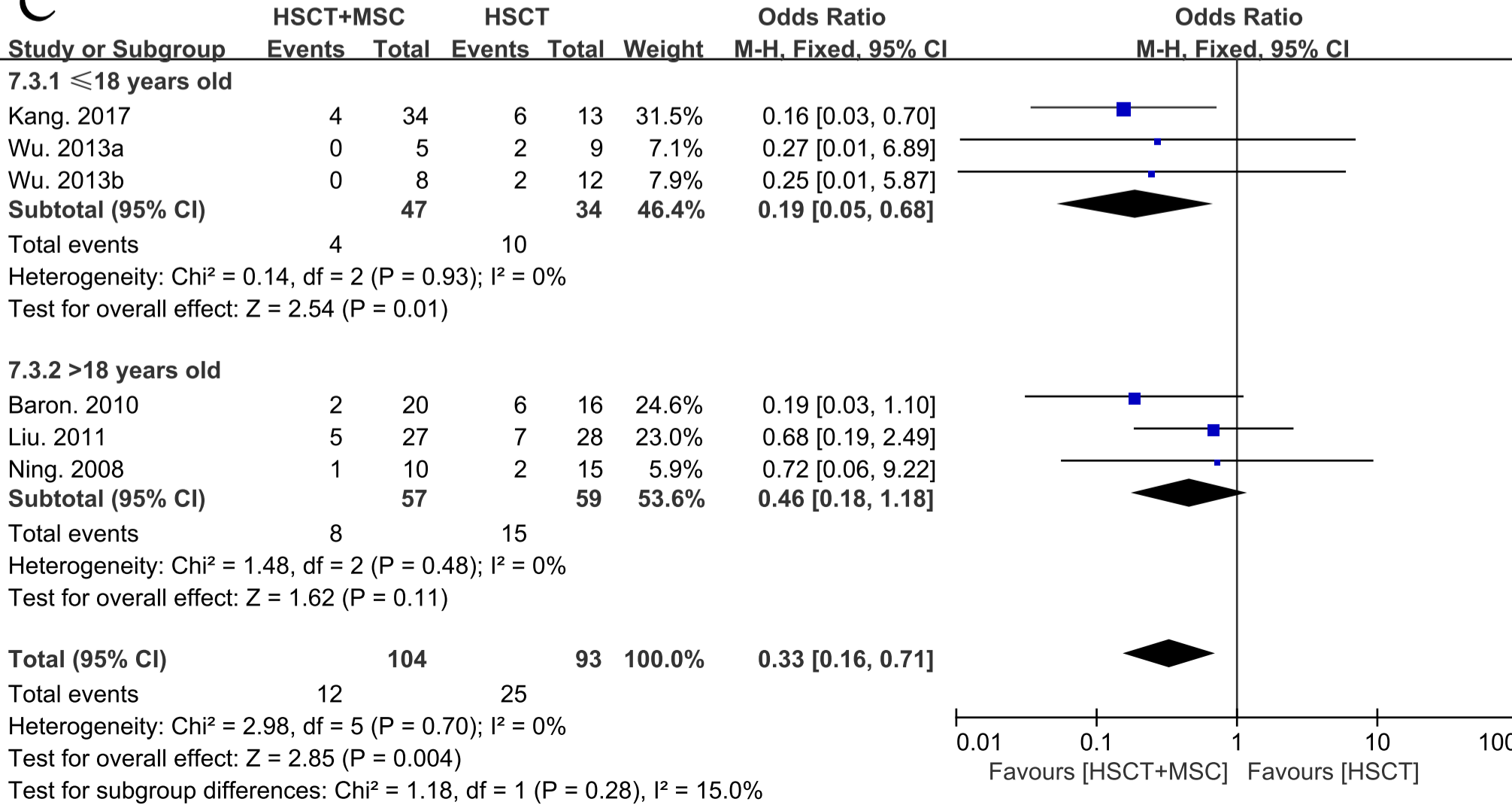

Supplement: Supplementary file 13 — Additional file 13: Fig. S11. Assessment of NRM in subgroup analysis according to (a) type of disease, (b) HLA matching and (c) average age. [file 13287_2021_2304_MOESM13_ESM.pdf]
