# Supplementary material for: Efficacy and safety of mesenchymal stem cells co-infusion in allogeneic hematopoietic stem cell transplantation: a systematic review and meta-analysis
Source: Stem Cell Res Ther. 2021 Apr 20;12:246. doi: 10.1186/s13287-021-02304-x (PMC8056684; doi:10.1186/s13287-021-02304-x)

a

Study or Subgroup	HSCT+MSC Events	HSCT+MSC Total	HSCT Events	HSCT Total	Weight	Risk Ratio M-H, Fixed, 95% CI
**3.1.1 Hematologic Malignancies**						
Bernardo. 2011	5	13	21	39	8.5%	0.71 [0.34, 1.51]
Daganzo. 2009	5	9	29	46	7.7%	0.88 [0.47, 1.65]
Kang. 2017	15	34	12	13	14.1%	0.48 [0.32, 0.72]
Lee. 2013	5	7	5	8	3.8%	1.14 [0.56, 2.33]
Liu. 2011	16	27	16	27	13.0%	1.00 [0.64, 1.56]
Ning. 2008	4	9	11	15	6.7%	0.61 [0.27, 1.34]
Wu. 2013b	4	8	8	12	5.2%	0.75 [0.34, 1.67]
Zhang. 2015	11	22	16	27	11.7%	0.84 [0.50, 1.42]
**Subtotal (95% CI)**		**129**		**187**	**70.8%**	**0.77 [0.63, 0.95]**
Total events	65		118			
Heterogeneity: Chi² = 8.45, df = 7 (P = 0.29); I² = 17%						
Test for overall effect: Z = 2.40 (P = 0.02)						
**3.1.2 Nonmalignant disorders**						
Ghavamzad. 2017	23	41	19	29	18.1%	0.86 [0.59, 1.25]
Wang. 2015	2	7	3	7	2.4%	0.67 [0.16, 2.84]
**Subtotal (95% CI)**		**48**		**36**	**20.5%**	**0.83 [0.58, 1.21]**
Total events	25		22			
Heterogeneity: Chi² = 0.11, df = 1 (P = 0.74); I² = 0%						
Test for overall effect: Z = 0.96 (P = 0.34)						
**3.1.3 Mixed**						
Ball. 2007	2	14	14	47	5.2%	0.48 [0.12, 1.86]
Wu. 2013a	2	5	6	9	3.5%	0.60 [0.19, 1.93]
**Subtotal (95% CI)**		**19**		**56**	**8.7%**	**0.53 [0.21, 1.33]**
Total events	4		20			
Heterogeneity: Chi² = 0.07, df = 1 (P = 0.80); I² = 0%						
Test for overall effect: Z = 1.36 (P = 0.17)						
**Total (95% CI)**		**196**		**279**	**100.0%**	**0.77 [0.64, 0.92]**
Total events	94		160			
Heterogeneity: Chi² = 9.39, df = 11 (P = 0.59); I² = 0%						
Test for overall effect: Z = 2.90 (P = 0.004)						
Test for subgroup differences: Chi² = 0.82, df = 2 (P = 0.67), I² = 0%						

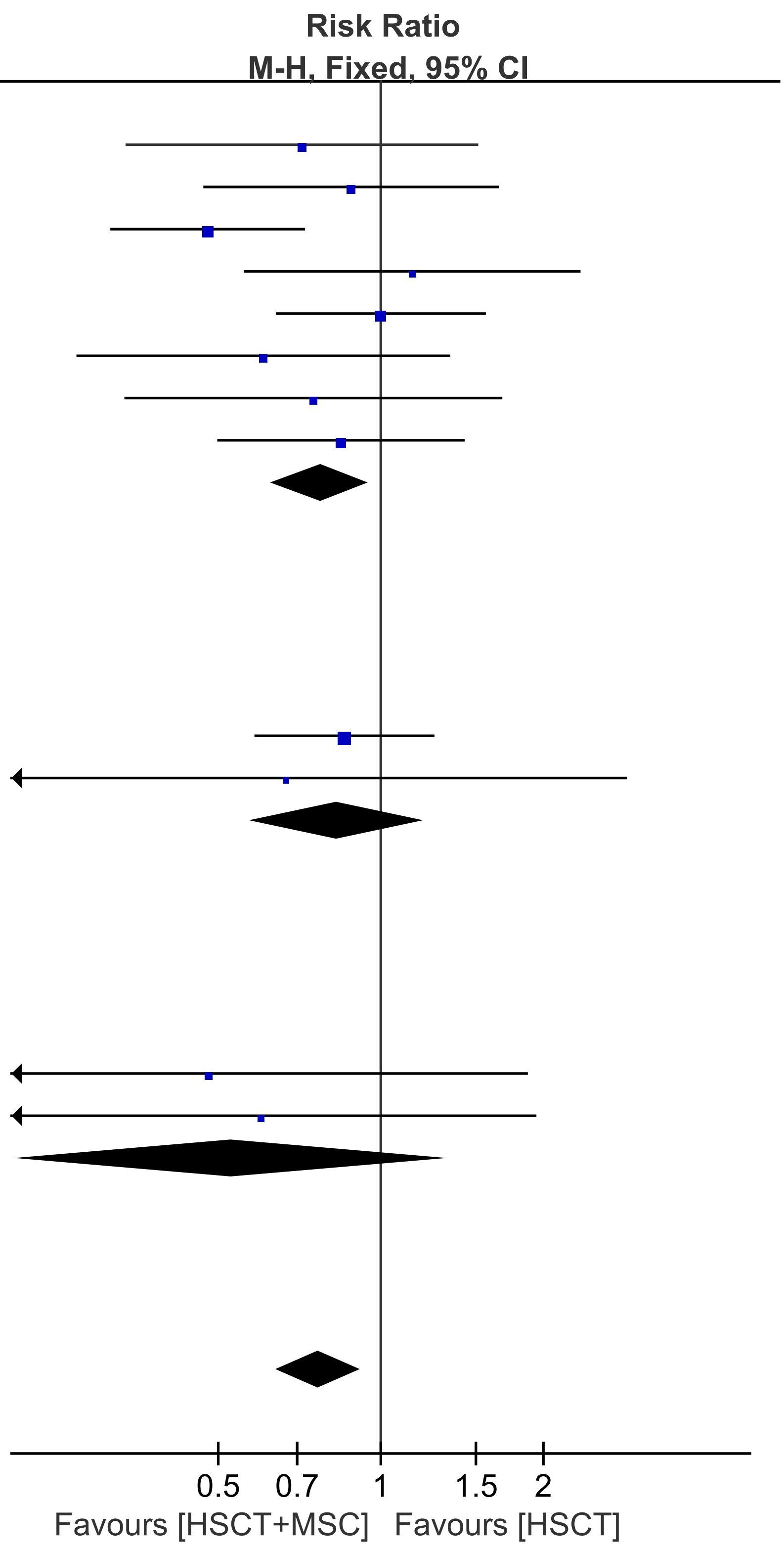

b

Study or Subgroup	HSCT+MSC Events	HSCT+MSC Total	HSCT Events	HSCT Total	Weight	Risk Ratio M-H, Fixed, 95% CI
**3.2.1 Identical**						
Ghavamzad. 2017	23	41	19	29	18.1%	0.86 [0.59, 1.25]
Ning. 2008	4	9	11	15	6.7%	0.61 [0.27, 1.34]
**Subtotal (95% CI)**		**50**		**44**	**24.8%**	**0.79 [0.56, 1.11]**
Total events	27		30			
Heterogeneity: Chi² = 0.61, df = 1 (P = 0.44); I² = 0%						
Test for overall effect: Z = 1.36 (P = 0.18)						
**3.2.2 Non-identical**						
Ball. 2007	2	14	14	47	5.2%	0.48 [0.12, 1.86]
Bernardo. 2011	5	13	21	39	8.5%	0.71 [0.34, 1.51]
Daganzo. 2009	5	9	29	46	7.7%	0.88 [0.47, 1.65]
Kang. 2017	15	34	12	13	14.1%	0.48 [0.32, 0.72]
Lee. 2013	5	7	5	8	3.8%	1.14 [0.56, 2.33]
Liu. 2011	16	27	16	27	13.0%	1.00 [0.64, 1.56]
Wang. 2015	2	7	3	7	2.4%	0.67 [0.16, 2.84]
Wu. 2013a	2	5	6	9	3.5%	0.60 [0.19, 1.93]
Wu. 2013b	4	8	8	12	5.2%	0.75 [0.34, 1.67]
Zhang. 2015	11	22	16	27	11.7%	0.84 [0.50, 1.42]
**Subtotal (95% CI)**		**146**		**235**	**75.2%**	**0.76 [0.61, 0.94]**
Total events	67		130			
Heterogeneity: Chi² = 8.68, df = 9 (P = 0.47); I² = 0%						
Test for overall effect: Z = 2.56 (P = 0.01)						
**Total (95% CI)**		**196**		**279**	**100.0%**	**0.77 [0.64, 0.92]**
Total events	94		160			
Heterogeneity: Chi² = 9.39, df = 11 (P = 0.59); I² = 0%						
Test for overall effect: Z = 2.90 (P = 0.004)						
Test for subgroup differences: Chi² = 0.04, df = 1 (P = 0.85), I² = 0%						

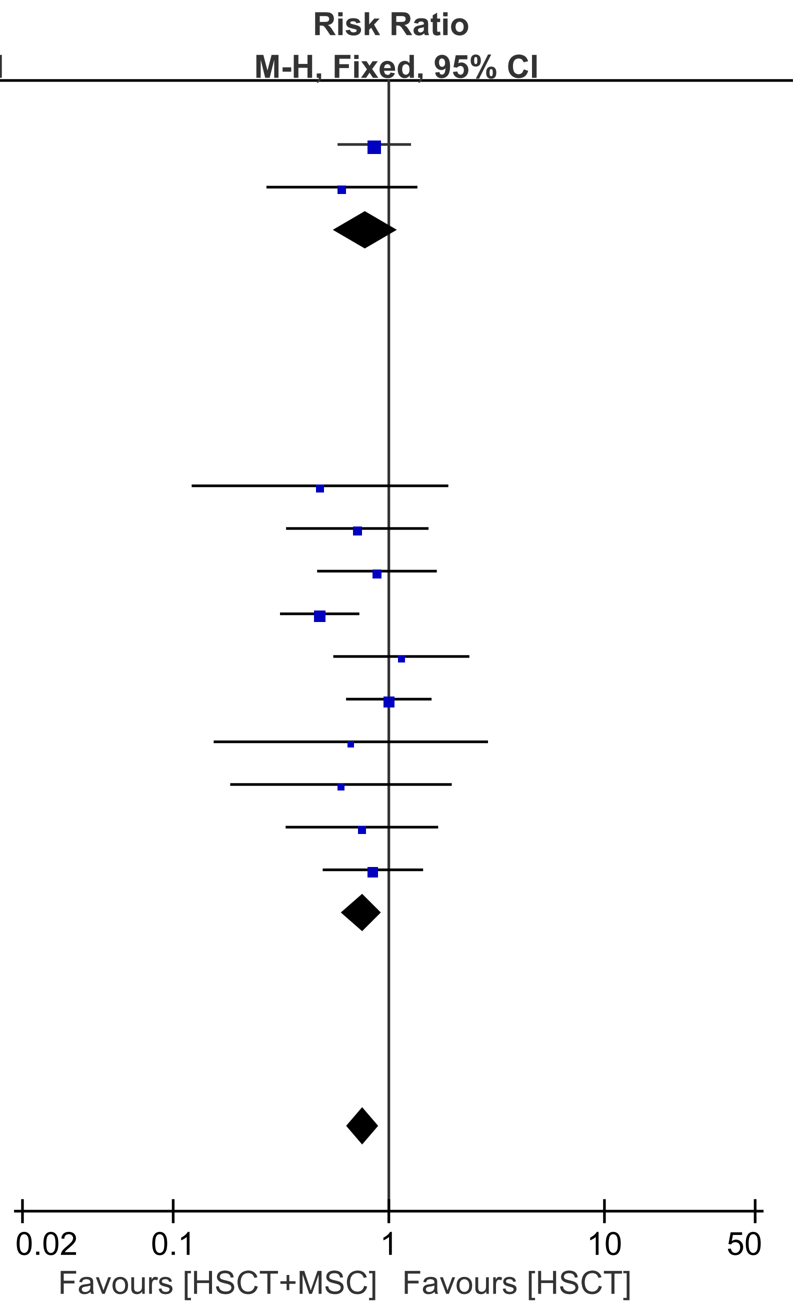

c

Study or Subgroup	HSCT+MSC Events	HSCT+MSC Total	HSCT Events	HSCT Total	Weight	Risk Ratio M-H, Fixed, 95% CI
**3.3.1 ≤18 years old**						
Ball. 2007	2	14	14	47	5.2%	0.48 [0.12, 1.86]
Bernardo. 2011	5	13	21	39	8.5%	0.71 [0.34, 1.51]
Kang. 2017	15	34	12	13	14.1%	0.48 [0.32, 0.72]
Lee. 2013	5	7	5	8	3.8%	1.14 [0.56, 2.33]
Wu. 2013a	2	5	6	9	3.5%	0.60 [0.19, 1.93]
Wu. 2013b	4	8	8	12	5.2%	0.75 [0.34, 1.67]
**Subtotal (95% CI)**		**81**		**128**	**40.4%**	**0.64 [0.47, 0.87]**
Total events	33		66			
Heterogeneity: Chi² = 4.90, df = 5 (P = 0.43); I² = 0%						
Test for overall effect: Z = 2.85 (P = 0.004)						
**3.3.2 >18 years old**						
Daganzo. 2009	5	9	29	46	7.7%	0.88 [0.47, 1.65]
Liu. 2011	16	27	16	27	13.0%	1.00 [0.64, 1.56]
Ning. 2008	4	9	11	15	6.7%	0.61 [0.27, 1.34]
Wang. 2015	2	7	3	7	2.4%	0.67 [0.16, 2.84]
Zhang. 2015	11	22	16	27	11.7%	0.84 [0.50, 1.42]
**Subtotal (95% CI)**		**74**		**122**	**41.5%**	**0.85 [0.65, 1.12]**
Total events	38		75			
Heterogeneity: Chi² = 1.34, df = 4 (P = 0.85); I² = 0%						
Test for overall effect: Z = 1.15 (P = 0.25)						
**3.3.3 Not Reported**						
Ghavamzad. 2017	23	41	19	29	18.1%	0.86 [0.59, 1.25]
**Subtotal (95% CI)**		**41**		**29**	**18.1%**	**0.86 [0.59, 1.25]**
Total events	23		19			
Heterogeneity: Not applicable						
Test for overall effect: Z = 0.80 (P = 0.42)						
**Total (95% CI)**		**196**		**279**	**100.0%**	**0.77 [0.64, 0.92]**
Total events	94		160			
Heterogeneity: Chi² = 9.39, df = 11 (P = 0.59); I² = 0%						
Test for overall effect: Z = 2.90 (P = 0.004)						
Test for subgroup differences: Chi² = 2.25, df = 2 (P = 0.32), I² = 11.3%						

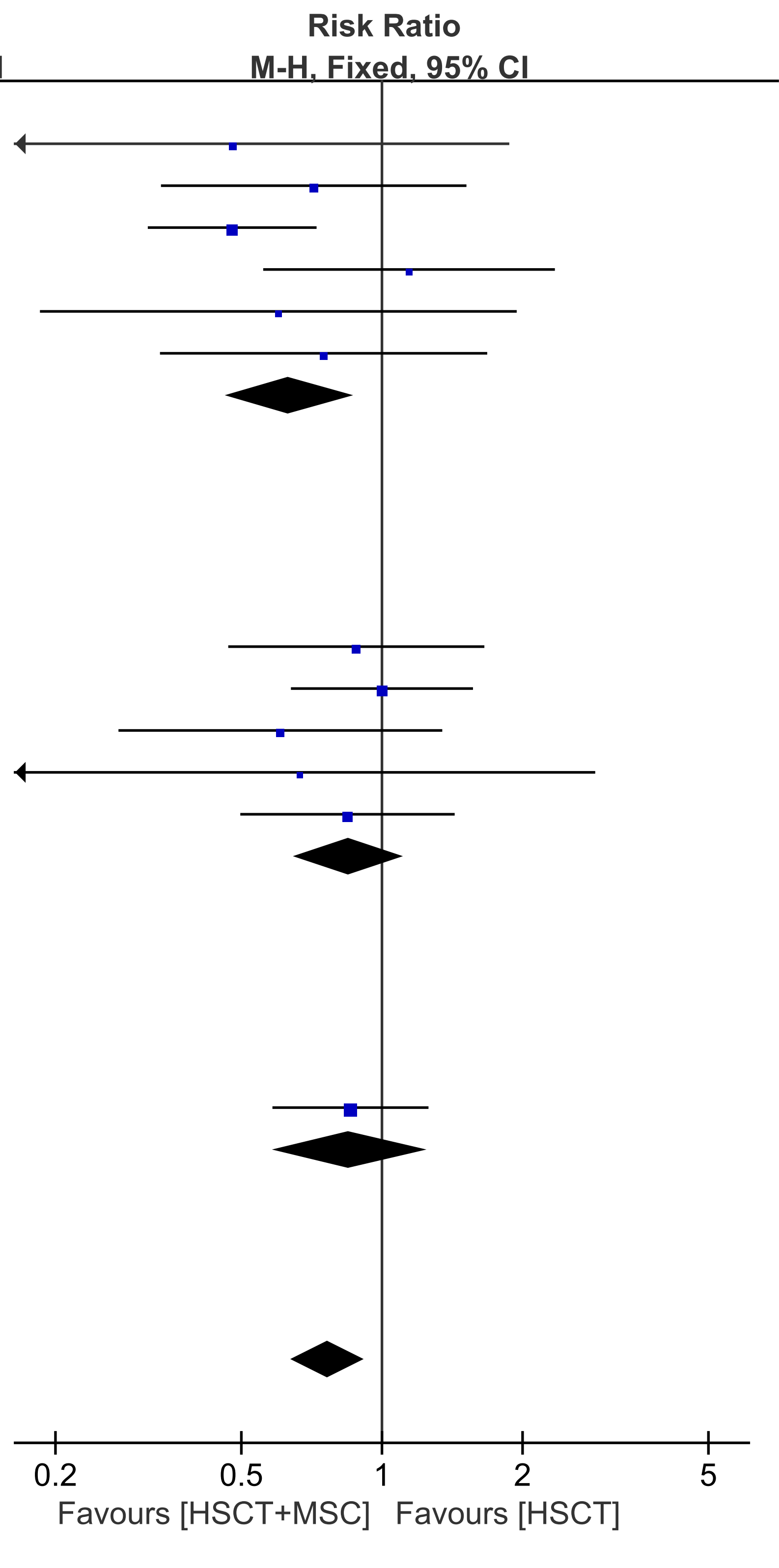

Supplement: Supplementary file 9 — Additional file 9: Fig. S7. Assessment of aGVHD in subgroup analysis according to (a) type of disease, (b) HLA matching and (c) average age. [file 13287_2021_2304_MOESM9_ESM.pdf]
