# Supplementary material for: Efficacy and safety of mesenchymal stem cells co-infusion in allogeneic hematopoietic stem cell transplantation: a systematic review and meta-analysis
Source: Stem Cell Res Ther. 2021 Apr 20;12:246. doi: 10.1186/s13287-021-02304-x (PMC8056684; doi:10.1186/s13287-021-02304-x)

a

Study or Subgroup	HSCT+MSC Events	HSCT+MSC Total	HSCT Events	HSCT Total	Weight	Risk Ratio M-H, Fixed, 95% CI
**6.1.1 Hematologic Malignancies**						
Baron. 2010	6	20	4	16	11.4%	1.20 [0.41, 3.54]
Bernardo. 2011	3	13	9	39	11.6%	1.00 [0.32, 3.15]
Kang. 2017	11	34	7	13	26.0%	0.60 [0.30, 1.21]
Liu. 2011	3	27	2	28	5.1%	1.56 [0.28, 8.59]
Mareika. 2016	1	10	0	12	1.2%	3.55 [0.16, 78.56]
Ning. 2008	6	10	3	15	6.2%	3.00 [0.97, 9.30]
Wu. 2013b	2	8	2	12	4.1%	1.50 [0.26, 8.58]
Xiang. 2017	5	32	8	32	20.6%	0.63 [0.23, 1.71]
Zhang. 2015	2	22	6	27	13.9%	0.41 [0.09, 1.83]
**Subtotal (95% CI)**		**176**		**194**	**100.0%**	**0.96 [0.66, 1.41]**
Total events	39		41			
Heterogeneity: Chi² = 8.98, df = 8 (P = 0.34); I² = 11%						
Test for overall effect: Z = 0.20 (P = 0.84)						
**6.1.2 Nonmalignant disorders**						
**Subtotal (95% CI)**		**0**		**0**		**Not estimable**
Total events	0		0			
Heterogeneity: Not applicable						
Test for overall effect: Not applicable						
**Total (95% CI)**		**176**		**194**	**100.0%**	**0.96 [0.66, 1.41]**
Total events	39		41			
Heterogeneity: Chi² = 8.98, df = 8 (P = 0.34); I² = 11%						
Test for overall effect: Z = 0.20 (P = 0.84)						
Test for subgroup differences: Not applicable						

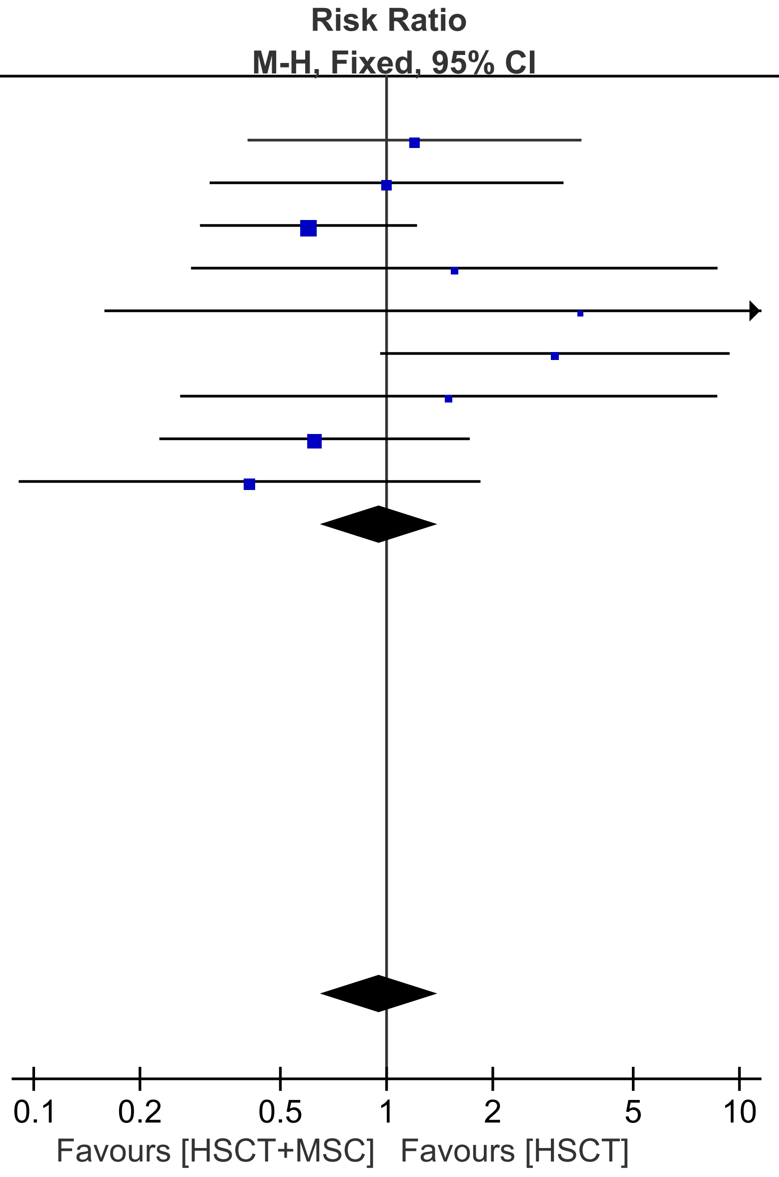

b

Study or Subgroup	HSCT+MSC Events	HSCT+MSC Total	HSCT Events	HSCT Total	Weight	Risk Ratio M-H, Random, 95% CI
**6.2.1 Identical**						
Ning. 2008	6	10	3	15	12.3%	3.00 [0.97, 9.30]
Xiang. 2017	5	32	8	32	15.1%	0.63 [0.23, 1.71]
**Subtotal (95% CI)**		**42**		**47**	**27.4%**	**1.34 [0.29, 6.25]**
Total events	11		11			
Heterogeneity: Tau² = 0.94; Chi² = 4.16, df = 1 (P = 0.04); I² = 76%						
Test for overall effect: Z = 0.37 (P = 0.71)						
**6.2.2 Non-identical**						
Baron. 2010	6	20	4	16	13.3%	1.20 [0.41, 3.54]
Bernardo. 2011	3	13	9	39	12.0%	1.00 [0.32, 3.15]
Kang. 2017	11	34	7	13	26.8%	0.60 [0.30, 1.21]
Liu. 2011	3	27	2	28	5.8%	1.56 [0.28, 8.59]
Wu. 2013b	2	8	2	12	5.6%	1.50 [0.26, 8.58]
Zhang. 2015	2	22	6	27	7.4%	0.41 [0.09, 1.83]
**Subtotal (95% CI)**		**124**		**135**	**70.8%**	**0.81 [0.51, 1.28]**
Total events	27		30			
Heterogeneity: Tau² = 0.00; Chi² = 3.20, df = 5 (P = 0.67); I² = 0%						
Test for overall effect: Z = 0.90 (P = 0.37)						
**6.2.3 Not Reported**						
Mareika. 2016	1	10	0	12	1.8%	3.55 [0.16, 78.56]
**Subtotal (95% CI)**		**10**		**12**	**1.8%**	**3.55 [0.16, 78.56]**
Total events	1		0			
Heterogeneity: Not applicable						
Test for overall effect: Z = 0.80 (P = 0.42)						
**Total (95% CI)**		**176**		**194**	**100.0%**	**0.96 [0.63, 1.46]**
Total events	39		41			
Heterogeneity: Tau² = 0.05; Chi² = 8.98, df = 8 (P = 0.34); I² = 11%						
Test for overall effect: Z = 0.20 (P = 0.84)						
Test for subgroup differences: Chi² = 1.18, df = 2 (P = 0.55), I² = 0%						

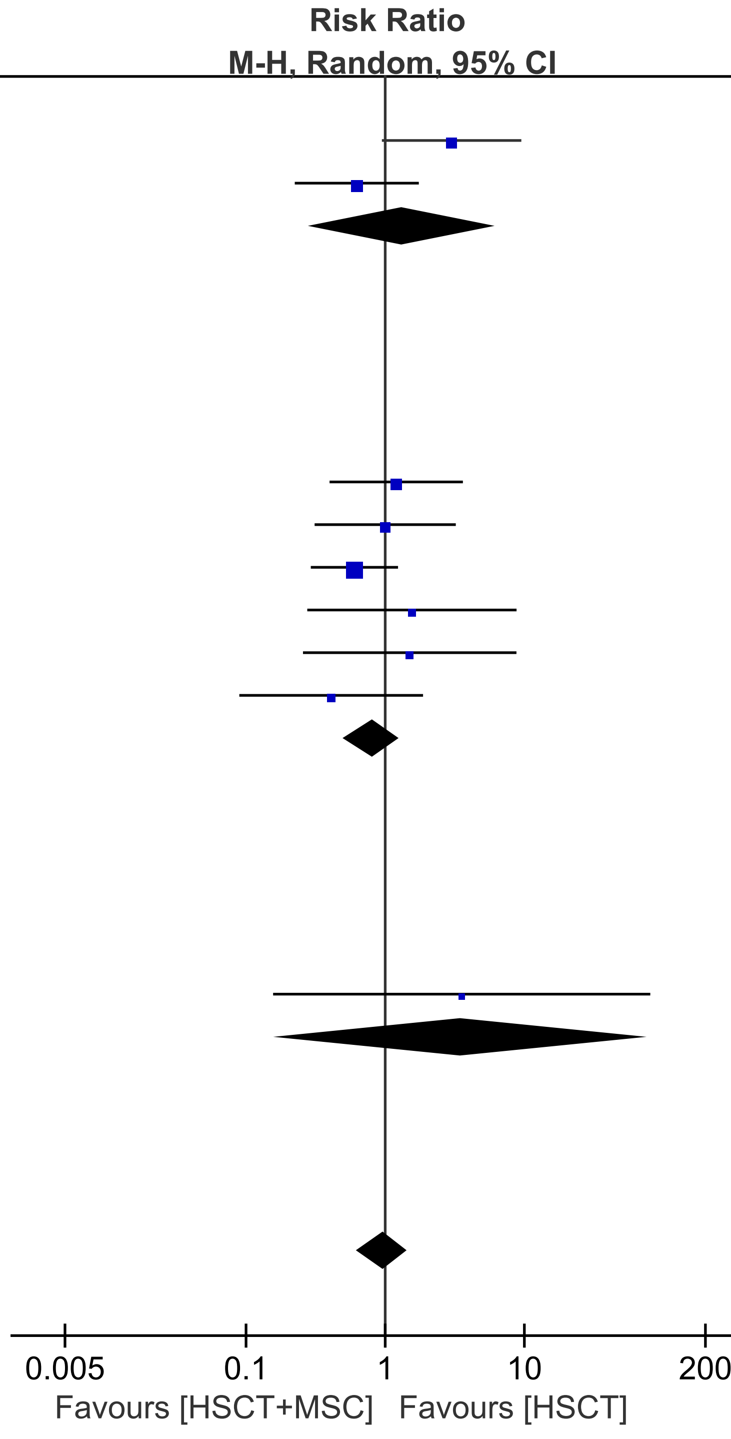

c

Study or Subgroup	HSCT+MSC Events	HSCT+MSC Total	HSCT Events	HSCT Total	Weight	Risk Ratio M-H, Fixed, 95% CI
**6.3.1 ≤18 years old**						
Bernardo. 2011	3	13	9	39	11.6%	1.00 [0.32, 3.15]
Kang. 2017	11	34	7	13	26.0%	0.60 [0.30, 1.21]
Mareika. 2016	1	10	0	12	1.2%	3.55 [0.16, 78.56]
Wu. 2013b	2	8	2	12	4.1%	1.50 [0.26, 8.58]
Xiang. 2017	5	32	8	32	20.6%	0.63 [0.23, 1.71]
**Subtotal (95% CI)**		**97**		**108**	**63.5%**	**0.79 [0.49, 1.30]**
Total events	22		26			
Heterogeneity: Chi² = 2.39, df = 4 (P = 0.66); I² = 0%						
Test for overall effect: Z = 0.92 (P = 0.36)						
**6.3.2 >18 years old**						
Baron. 2010	6	20	4	16	11.4%	1.20 [0.41, 3.54]
Liu. 2011	3	27	2	28	5.1%	1.56 [0.28, 8.59]
Ning. 2008	6	10	3	15	6.2%	3.00 [0.97, 9.30]
Zhang. 2015	2	22	6	27	13.9%	0.41 [0.09, 1.83]
**Subtotal (95% CI)**		**79**		**86**	**36.5%**	**1.25 [0.68, 2.31]**
Total events	17		15			
Heterogeneity: Chi² = 4.50, df = 3 (P = 0.21); I² = 33%						
Test for overall effect: Z = 0.73 (P = 0.47)						
**Total (95% CI)**		**176**		**194**	**100.0%**	**0.96 [0.66, 1.41]**
Total events	39		41			
Heterogeneity: Chi² = 8.98, df = 8 (P = 0.34); I² = 11%						
Test for overall effect: Z = 0.20 (P = 0.84)						
Test for subgroup differences: Chi² = 1.31, df = 1 (P = 0.25), I² = 23.4%						

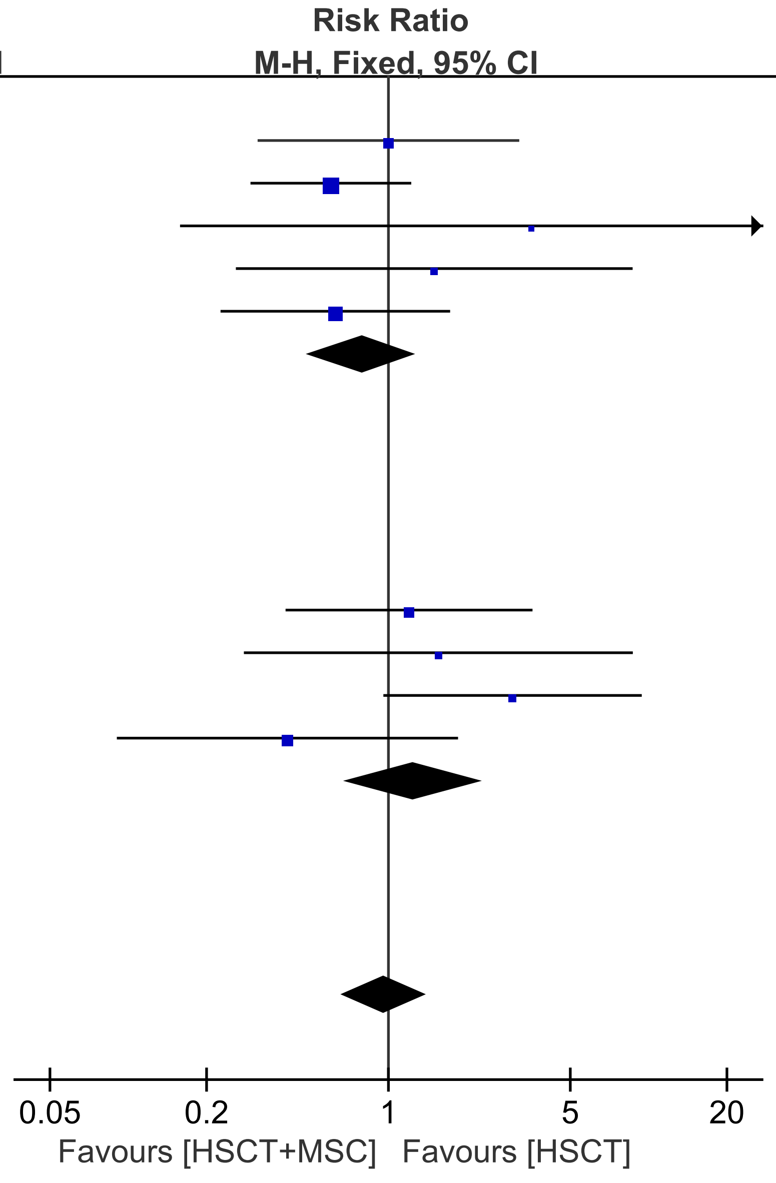

Supplement: Supplementary file 12 — Additional file 12: Fig. S10. Assessment of RR in subgroup analysis according to (a) type of disease, (b) HLA matching and (c) average age. [file 13287_2021_2304_MOESM12_ESM.pdf]
