# Supplementary material for: Efficacy and safety of mesenchymal stem cells co-infusion in allogeneic hematopoietic stem cell transplantation: a systematic review and meta-analysis
Source: Stem Cell Res Ther. 2021 Apr 20;12:246. doi: 10.1186/s13287-021-02304-x (PMC8056684; doi:10.1186/s13287-021-02304-x)

a

Study or Subgroup	HSCT+MSC Mean	HSCT+MSC SD	HSCT+MSC Total	HSCT Mean	HSCT SD	HSCT Total	Weight	Std. Mean Difference IV, Random, 95% CI
**1.2.1 Hematologic Malignancies**								
Bernardo. 2011	29.82	7.22	13	28.1	8.95	39	8.2%	0.20 [-0.43, 0.83]
Daganzo. 2009	15.7	6.54	9	12.31	8.59	46	7.9%	0.40 [-0.32, 1.12]
Hou. 2010	13.2	2.79	15	12.84	3.17	20	8.0%	0.12 [-0.55, 0.79]
Lee. 2013	18.74	1.45	7	24.65	4.93	9	6.4%	-1.45 [-2.60, -0.31]
MacMillan. 2009	18.52	5.78	7	16.52	5.71	23	7.4%	0.34 [-0.51, 1.19]
Mareika. 2016	17.75	2.47	10	26.49	8.58	12	7.1%	-1.28 [-2.22, -0.34]
Ning. 2008	16.25	2.602	9	15.25	2.602	15	7.5%	0.37 [-0.46, 1.21]
Wu. 2013b	12	2.309	8	25.5	7.943	12	6.4%	-2.03 [-3.17, -0.89]
Xiang. 2017	12.25	1.59	32	15.96	2.2	32	8.3%	-1.91 [-2.51, -1.31]
Zhang. 2015	12.99	3.89	22	15.63	4.96	27	8.3%	-0.58 [-1.15, -0.00]
**Subtotal (95% CI)**			**132**			**235**	**75.4%**	**-0.54 [-1.14, 0.06]**
Heterogeneity: Tau² = 0.77; Chi² = 55.66, df = 9 (P < 0.00001); I² = 84%								
Test for overall effect: Z = 1.77 (P = 0.08)								
**1.2.2 Nonmalignant disorders**								
Wang. 2015	17.24	2.037	7	21.48	1.76	7	5.6%	-2.09 [-3.47, -0.70]
**Subtotal (95% CI)**			**7**			**7**	**5.6%**	**-2.09 [-3.47, -0.70]**
Heterogeneity: Not applicable								
Test for overall effect: Z = 2.94 (P = 0.003)								
**1.2.3 Mixed**								
Ball. 2007	12.53	2.07	14	14	5.71	47	8.2%	-0.28 [-0.88, 0.32]
Wu. 2013a	10.5	1.76	5	27	5.89	9	4.5%	-3.14 [-4.90, -1.39]
Xiao. 2013	12.14	2.73	7	20.5	7.65	8	6.3%	-1.33 [-2.49, -0.17]
**Subtotal (95% CI)**			**26**			**64**	**19.1%**	**-1.39 [-2.86, 0.07]**
Heterogeneity: Tau² = 1.31; Chi² = 10.44, df = 2 (P = 0.005); I² = 81%								
Test for overall effect: Z = 1.86 (P = 0.06)								
**Total (95% CI)**			**165**			**306**	**100.0%**	**-0.77 [-1.29, -0.26]**
Heterogeneity: Tau² = 0.75; Chi² = 71.56, df = 13 (P < 0.00001); I² = 82%								
Test for overall effect: Z = 2.93 (P = 0.003)								
Test for subgroup differences: Chi² = 4.57, df = 2 (P = 0.10), I² = 56.2%								

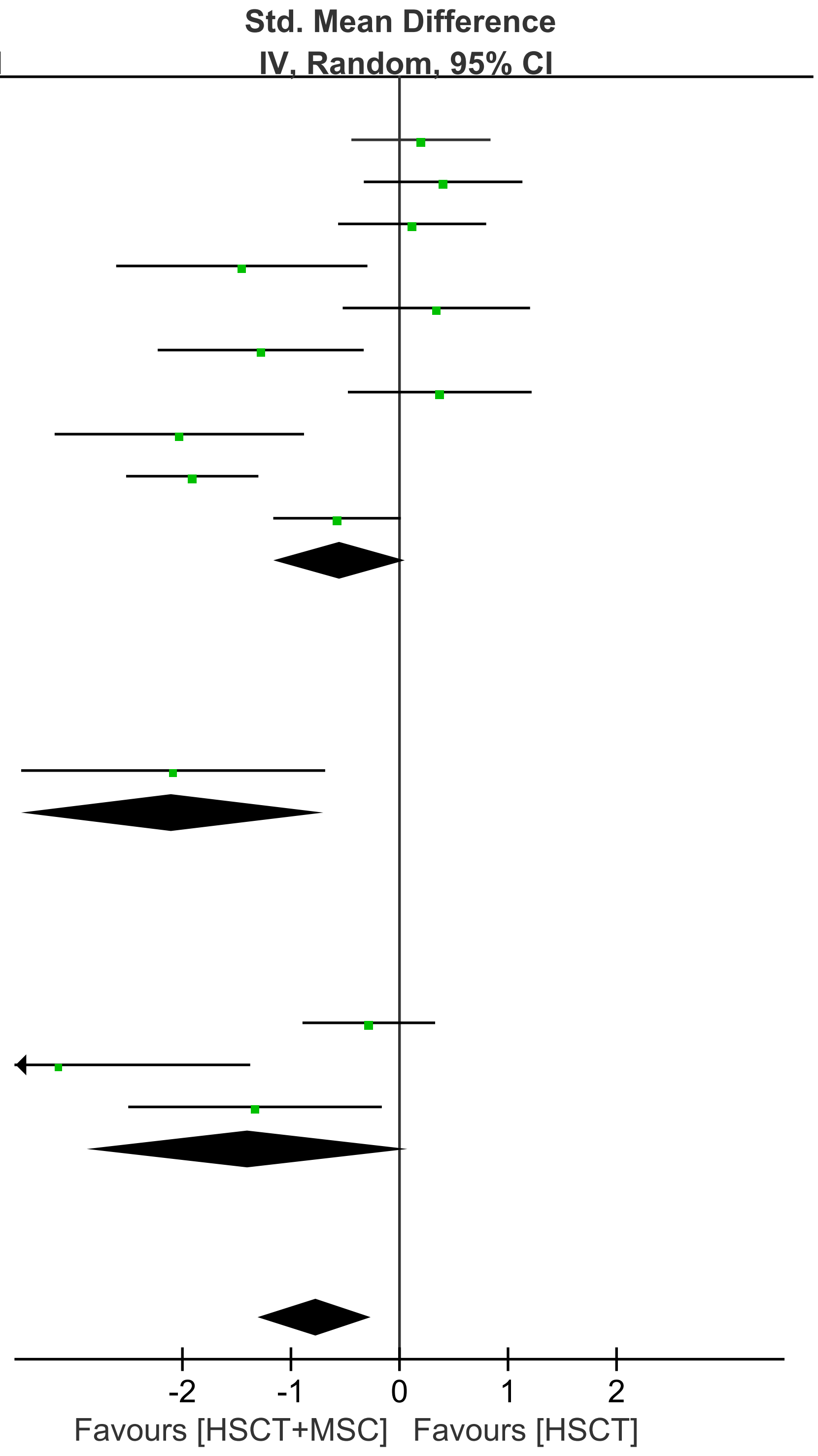

b

Study or Subgroup	HSCT+MSC Mean	HSCT+MSC SD	HSCT+MSC Total	HSCT Mean	HSCT SD	HSCT Total	Weight	Std. Mean Difference IV, Random, 95% CI
**1.3.1 Identical**								
Ning. 2008	16.25	2.602	9	15.25	2.602	15	7.5%	0.37 [-0.46, 1.21]
Xiang. 2017	12.25	1.59	32	15.96	2.2	32	8.3%	-1.91 [-2.51, -1.31]
**Subtotal (95% CI)**			**41**			**47**	**15.7%**	**-0.79 [-3.02, 1.45]**
Heterogeneity: Tau² = 2.46; Chi² = 18.98, df = 1 (P < 0.0001); I² = 95%								
Test for overall effect: Z = 0.69 (P = 0.49)								
**1.3.2 Non-identical**								
Ball. 2007	12.53	2.07	14	14	5.71	47	8.2%	-0.28 [-0.88, 0.32]
Bernardo. 2011	29.82	7.22	13	28.1	8.95	39	8.2%	0.20 [-0.43, 0.83]
Daganzo. 2009	15.7	6.54	9	12.31	8.59	46	7.9%	0.40 [-0.32, 1.12]
Hou. 2010	13.2	2.79	15	12.84	3.17	20	8.0%	0.12 [-0.55, 0.79]
Lee. 2013	18.74	1.45	7	24.65	4.93	9	6.4%	-1.45 [-2.60, -0.31]
MacMillan. 2009	18.52	5.78	7	16.52	5.71	23	7.4%	0.34 [-0.51, 1.19]
Wang. 2015	17.24	2.037	7	21.48	1.76	7	5.6%	-2.09 [-3.47, -0.70]
Wu. 2013a	10.5	1.76	5	27	5.89	9	4.5%	-3.14 [-4.90, -1.39]
Wu. 2013b	12	2.309	8	25.5	7.943	12	6.4%	-2.03 [-3.17, -0.89]
Zhang. 2015	12.99	3.89	22	15.63	4.96	27	8.3%	-0.58 [-1.15, -0.00]
**Subtotal (95% CI)**			**107**			**239**	**70.8%**	**-0.63 [-1.18, -0.07]**
Heterogeneity: Tau² = 0.57; Chi² = 39.77, df = 9 (P < 0.00001); I² = 77%								
Test for overall effect: Z = 2.23 (P = 0.03)								
**1.3.3 Not Reported**								
Mareika. 2016	17.75	2.47	10	26.49	8.58	12	7.1%	-1.28 [-2.22, -0.34]
Xiao. 2013	12.14	2.73	7	20.5	7.65	8	6.3%	-1.33 [-2.49, -0.17]
**Subtotal (95% CI)**			**17**			**20**	**13.4%**	**-1.30 [-2.03, -0.57]**
Heterogeneity: Tau² = 0.00; Chi² = 0.00, df = 1 (P = 0.95); I² = 0%								
Test for overall effect: Z = 3.50 (P = 0.0005)								
**Total (95% CI)**			**165**			**306**	**100.0%**	**-0.77 [-1.29, -0.26]**
Heterogeneity: Tau² = 0.75; Chi² = 71.56, df = 13 (P < 0.00001); I² = 82%								
Test for overall effect: Z = 2.93 (P = 0.003)								
Test for subgroup differences: Chi² = 2.08, df = 2 (P = 0.35), I² = 4.0%								

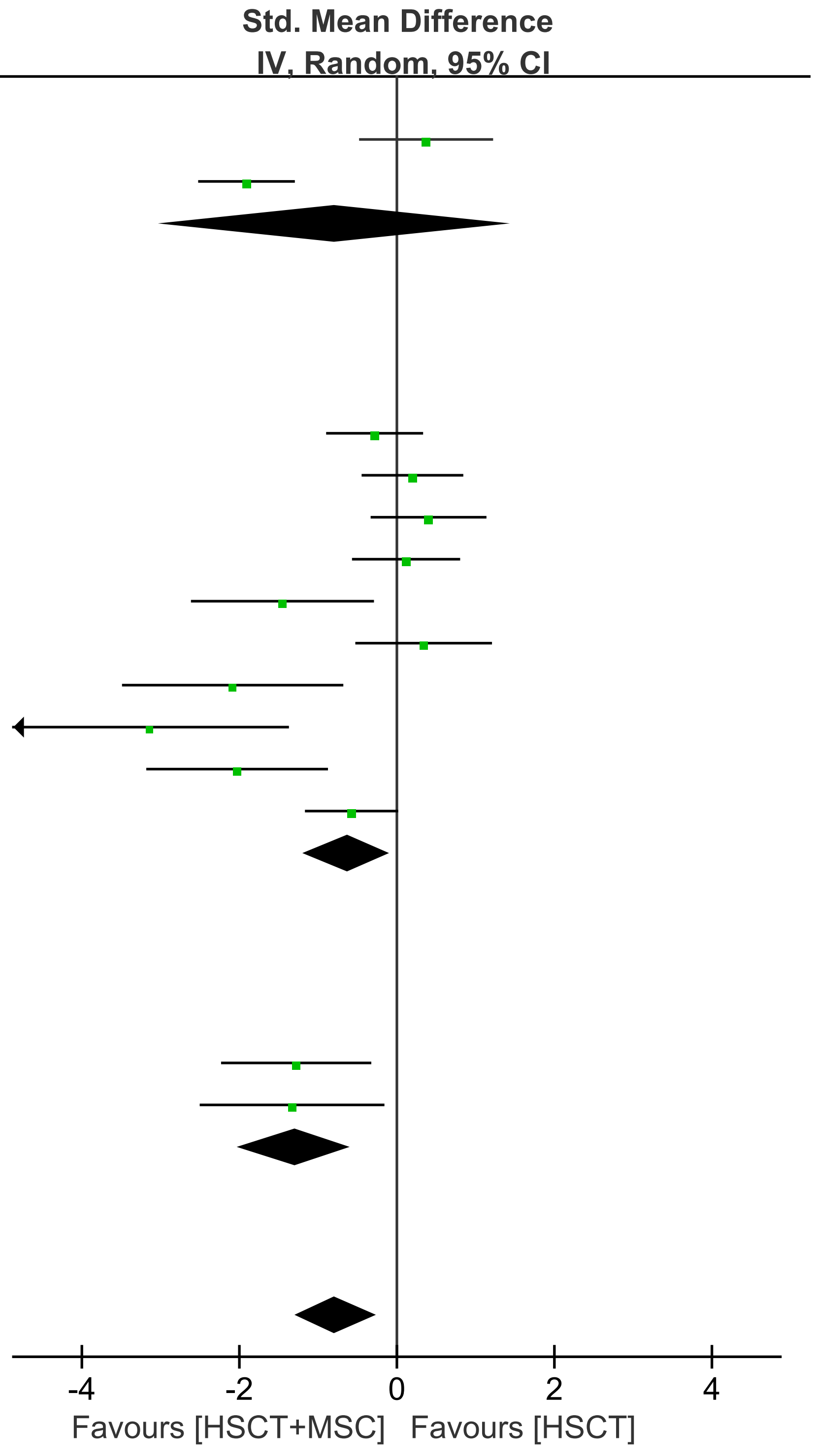

c

Study or Subgroup	HSCT+MSC Mean	HSCT+MSC SD	HSCT+MSC Total	HSCT Mean	HSCT SD	HSCT Total	Weight	Std. Mean Difference IV, Random, 95% CI
**1.4.1 ≤18 years old**								
Ball. 2007	12.53	2.07	14	14	5.71	47	8.2%	-0.28 [-0.88, 0.32]
Bernardo. 2011	29.82	7.22	13	28.1	8.95	39	8.2%	0.20 [-0.43, 0.83]
Lee. 2013	18.74	1.45	7	24.65	4.93	9	6.4%	-1.45 [-2.60, -0.31]
MacMillan. 2009	18.52	5.78	7	16.52	5.71	23	7.4%	0.34 [-0.51, 1.19]
Mareika. 2016	17.75	2.47	10	26.49	8.58	12	7.1%	-1.28 [-2.22, -0.34]
Wu. 2013a	10.5	1.76	5	27	5.89	9	4.5%	-3.14 [-4.90, -1.39]
Wu. 2013b	12	2.309	8	25.5	7.943	12	6.4%	-2.03 [-3.17, -0.89]
Xiang. 2017	12.25	1.59	32	15.96	2.2	32	8.3%	-1.91 [-2.51, -1.31]
**Subtotal (95% CI)**			**96**			**183**	**56.4%**	**-1.07 [-1.84, -0.31]**
Heterogeneity: Tau² = 0.98; Chi² = 46.23, df = 7 (P < 0.00001); I² = 85%								
Test for overall effect: Z = 2.74 (P = 0.006)								
**1.4.2 >18 years old**								
Daganzo. 2009	15.7	6.54	9	12.31	8.59	46	7.9%	0.40 [-0.32, 1.12]
Hou. 2010	13.2	2.79	15	12.84	3.17	20	8.0%	0.12 [-0.55, 0.79]
Ning. 2008	16.25	2.602	9	15.25	2.602	15	7.5%	0.37 [-0.46, 1.21]
Wang. 2015	17.24	2.037	7	21.48	1.76	7	5.6%	-2.09 [-3.47, -0.70]
Xiao. 2013	12.14	2.73	7	20.5	7.65	8	6.3%	-1.33 [-2.49, -0.17]
Zhang. 2015	12.99	3.89	22	15.63	4.96	27	8.3%	-0.58 [-1.15, -0.00]
**Subtotal (95% CI)**			**69**			**123**	**43.6%**	**-0.37 [-1.00, 0.26]**
Heterogeneity: Tau² = 0.42; Chi² = 17.72, df = 5 (P = 0.003); I² = 72%								
Test for overall effect: Z = 1.15 (P = 0.25)								
**Total (95% CI)**			**165**			**306**	**100.0%**	**-0.77 [-1.29, -0.26]**
Heterogeneity: Tau² = 0.75; Chi² = 71.56, df = 13 (P < 0.00001); I² = 82%								
Test for overall effect: Z = 2.93 (P = 0.003)								
Test for subgroup differences: Chi² = 1.93, df = 1 (P = 0.16), I² = 48.2%								

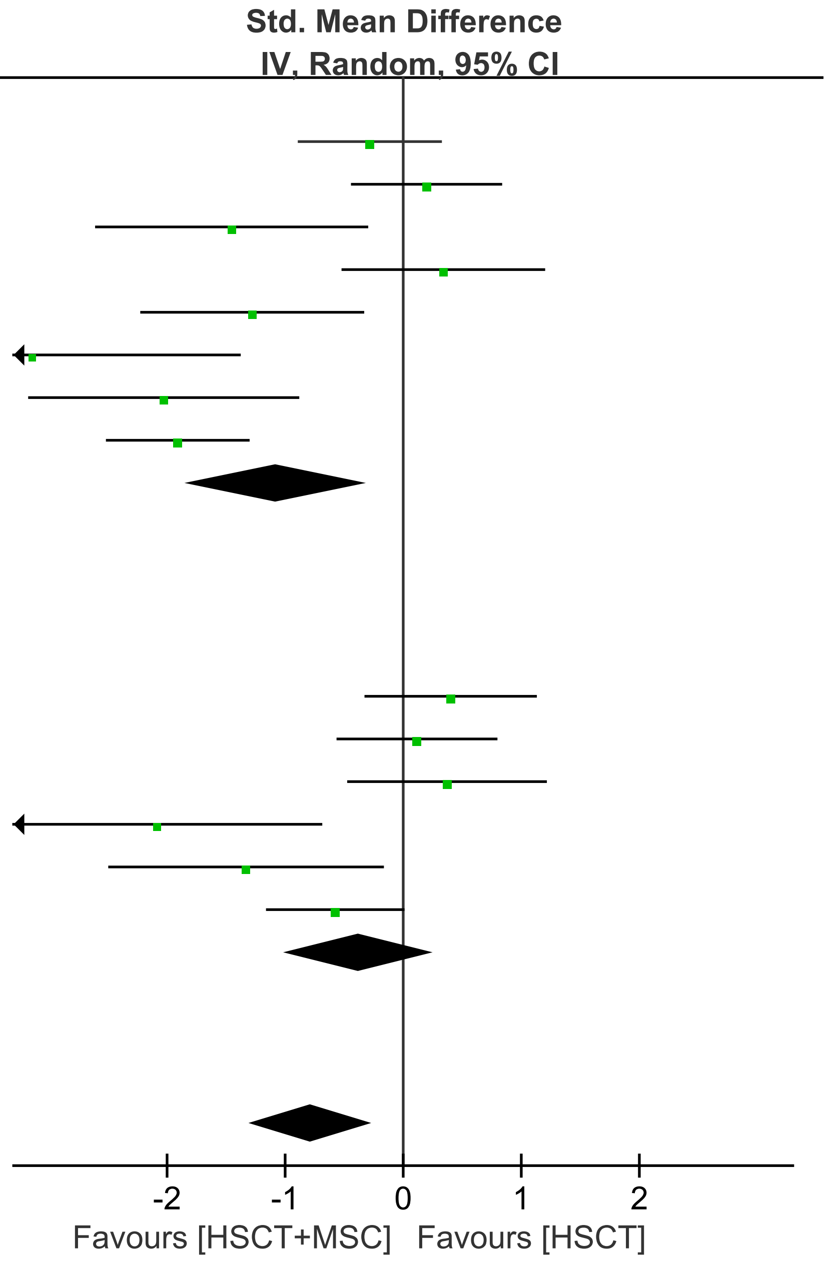

Supplement: Supplementary file 7 — Additional file 7: Fig. S5. Assessment of neutrophil engraftment in subgroup analysis according to (a) type of disease, (b) HLA matching and (c) average age. [file 13287_2021_2304_MOESM7_ESM.pdf]
