# Supplementary material for: Efficacy and safety of mesenchymal stem cells co-infusion in allogeneic hematopoietic stem cell transplantation: a systematic review and meta-analysis
Source: Stem Cell Res Ther. 2021 Apr 20;12:246. doi: 10.1186/s13287-021-02304-x (PMC8056684; doi:10.1186/s13287-021-02304-x)

a

	HSCT+MSC			HSCT				Std. Mean Difference
Study or Subgroup	Mean	SD	Total	Mean	SD	Total	Weight	IV, Random, 95% CI
**2.1.1 Hematologic malignancies**								
Bernardo. 2011	38.45	27.11	13	39.77	21.74	39	8.2%	-0.06 [-0.68, 0.57]
Daganzo. 2009	52.05	21.18	9	36.25	25.14	46	7.7%	0.63 [-0.09, 1.36]
Hou. 2010	22.27	10.47	15	26.43	20.92	20	8.0%	-0.24 [-0.91, 0.44]
Lee. 2013	51.58	13.84	7	62.87	17.47	9	6.1%	-0.67 [-1.69, 0.36]
Liu. 2011	22.21	13.84	26	26.59	22.8	25	8.6%	-0.23 [-0.78, 0.32]
Mareika. 2016	21.74	10.15	10	44.82	40.45	12	6.9%	-0.72 [-1.59, 0.15]
Wu. 2013b	31.14	8.79	8	78.94	30.91	12	5.8%	-1.85 [-2.95, -0.75]
Xiang. 2017	15.42	2.59	32	16.02	2.86	32	8.9%	-0.22 [-0.71, 0.27]
Zhang. 2015	17.4	8.72	22	19.16	10.73	27	8.6%	-0.18 [-0.74, 0.39]
**Subtotal (95% CI)**			**142**			**222**	**68.8%**	**-0.28 [-0.61, 0.04]**
Heterogeneity: Tau² = 0.12; Chi² = 16.02, df = 8 (P = 0.04); I² = 50%								
Test for overall effect: Z = 1.70 (P = 0.09)								
**2.1.2 Nonmalignant disorders**								
Ghavamzad. 2017	27.06	2.61	41	36.45	8.09	29	8.6%	-1.67 [-2.22, -1.11]
Wang. 2015	19.48	1.19	7	21.96	1.83	7	5.2%	-1.50 [-2.74, -0.27]
**Subtotal (95% CI)**			**48**			**36**	**13.8%**	**-1.64 [-2.15, -1.14]**
Heterogeneity: Tau² = 0.00; Chi² = 0.06, df = 1 (P = 0.81); I² = 0%								
Test for overall effect: Z = 6.36 (P < 0.00001)								
**2.1.3 Mixed**								
Ball. 2007	11.25	2.79	14	20.56	28.87	47	8.4%	-0.36 [-0.96, 0.24]
Wu. 2013a	31.73	5.49	5	75.52	17.29	9	3.7%	-2.83 [-4.48, -1.18]
Xiao. 2013	8.71	6.02	7	19.5	7.15	8	5.3%	-1.53 [-2.72, -0.33]
**Subtotal (95% CI)**			**26**			**64**	**17.4%**	**-1.41 [-2.79, -0.03]**
Heterogeneity: Tau² = 1.14; Chi² = 9.30, df = 2 (P = 0.010); I² = 78%								
Test for overall effect: Z = 2.00 (P = 0.05)								
**Total (95% CI)**			**216**			**322**	**100.0%**	**-0.66 [-1.06, -0.27]**
Heterogeneity: Tau² = 0.40; Chi² = 51.82, df = 13 (P < 0.00001); I² = 75%								
Test for overall effect: Z = 3.28 (P = 0.001)								
Test for subgroup differences: Chi² = 20.61, df = 2 (P < 0.0001), I² = 90.3%								

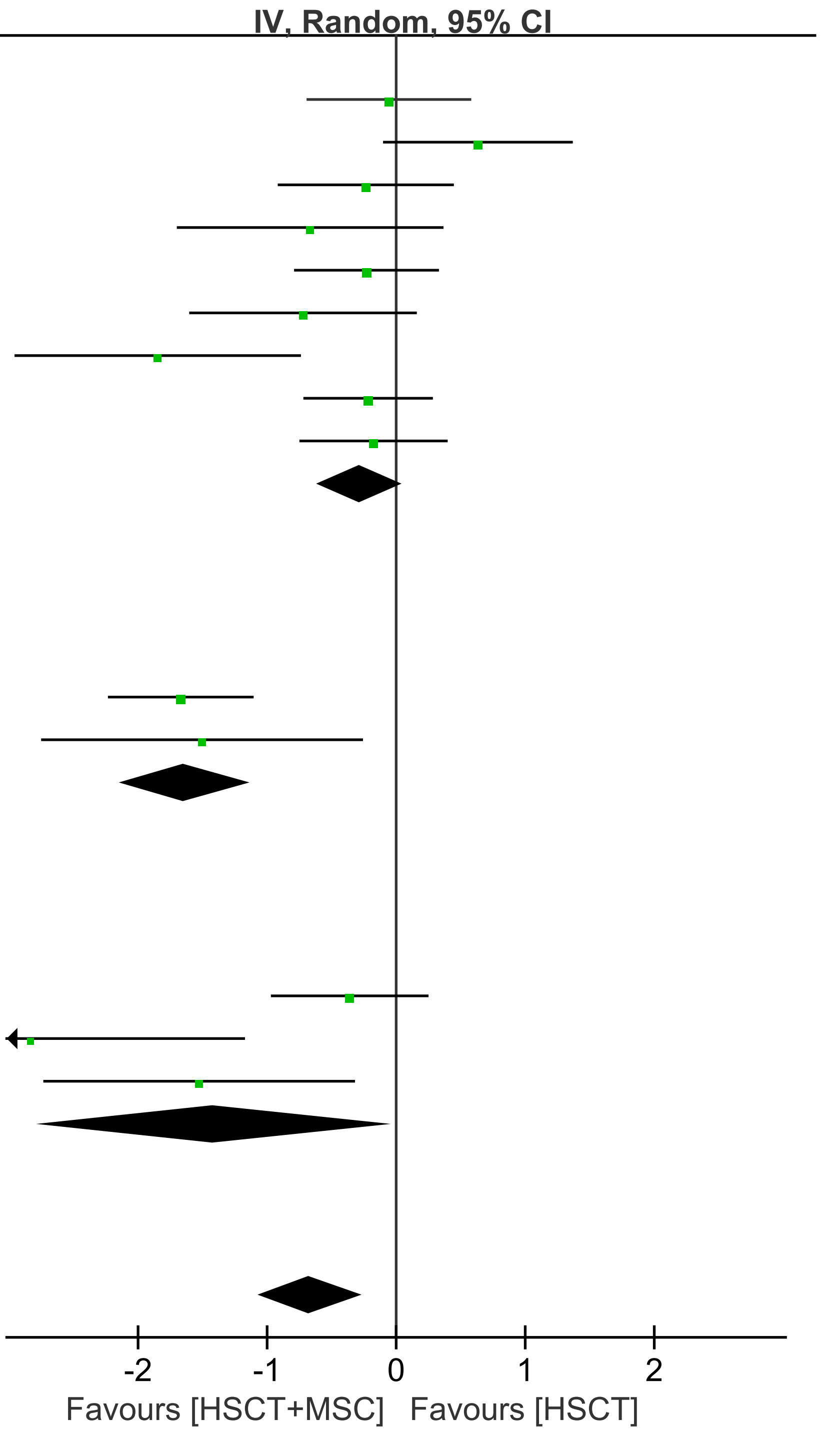

b

	HSCT+MSC			HSCT				Std. Mean Difference
Study or Subgroup	Mean	SD	Total	Mean	SD	Total	Weight	IV, Random, 95% CI
**2.2.1 Identical**								
Ghavamzad. 2017	27.06	2.61	41	36.45	8.09	29	8.6%	-1.67 [-2.22, -1.11]
Xiang. 2017	15.42	2.59	32	16.02	2.86	32	8.9%	-0.22 [-0.71, 0.27]
**Subtotal (95% CI)**			**73**			**61**	**17.5%**	**-0.94 [-2.36, 0.49]**
Heterogeneity: Tau² = 0.98; Chi² = 14.75, df = 1 (P = 0.0001); I² = 93%								
Test for overall effect: Z = 1.29 (P = 0.20)								
**2.2.2 Non-identical**								
Ball. 2007	11.25	2.79	14	20.56	28.87	47	8.4%	-0.36 [-0.96, 0.24]
Bernardo. 2011	38.45	27.11	13	39.77	21.74	39	8.2%	-0.06 [-0.68, 0.57]
Daganzo. 2009	52.05	21.18	9	36.25	25.14	46	7.7%	0.63 [-0.09, 1.36]
Hou. 2010	22.27	10.47	15	26.43	20.92	20	8.0%	-0.24 [-0.91, 0.44]
Lee. 2013	51.58	13.84	7	62.87	17.47	9	6.1%	-0.67 [-1.69, 0.36]
Liu. 2011	22.21	13.84	26	26.59	22.8	25	8.6%	-0.23 [-0.78, 0.32]
Wang. 2015	19.48	1.19	7	21.96	1.83	7	5.2%	-1.50 [-2.74, -0.27]
Wu. 2013a	31.73	5.49	5	75.52	17.29	9	3.7%	-2.83 [-4.48, -1.18]
Wu. 2013b	31.14	8.79	8	78.94	30.91	12	5.8%	-1.85 [-2.95, -0.75]
Zhang. 2015	17.4	8.72	22	19.16	10.73	27	8.6%	-0.18 [-0.74, 0.39]
**Subtotal (95% CI)**			**126**			**241**	**70.2%**	**-0.50 [-0.93, -0.07]**
Heterogeneity: Tau² = 0.31; Chi² = 28.04, df = 9 (P = 0.0009); I² = 68%								
Test for overall effect: Z = 2.27 (P = 0.02)								
**2.2.3 Not Reported**								
Mareika. 2016	21.74	10.15	10	44.82	40.45	12	6.9%	-0.72 [-1.59, 0.15]
Xiao. 2013	8.71	6.02	7	19.5	7.15	8	5.3%	-1.53 [-2.72, -0.33]
**Subtotal (95% CI)**			**17**			**20**	**12.2%**	**-1.02 [-1.77, -0.26]**
Heterogeneity: Tau² = 0.04; Chi² = 1.13, df = 1 (P = 0.29); I² = 12%								
Test for overall effect: Z = 2.62 (P = 0.009)								
**Total (95% CI)**			**216**			**322**	**100.0%**	**-0.66 [-1.06, -0.27]**
Heterogeneity: Tau² = 0.40; Chi² = 51.82, df = 13 (P < 0.00001); I² = 75%								
Test for overall effect: Z = 3.28 (P = 0.001)								
Test for subgroup differences: Chi² = 1.50, df = 2 (P = 0.47), I² = 0%								

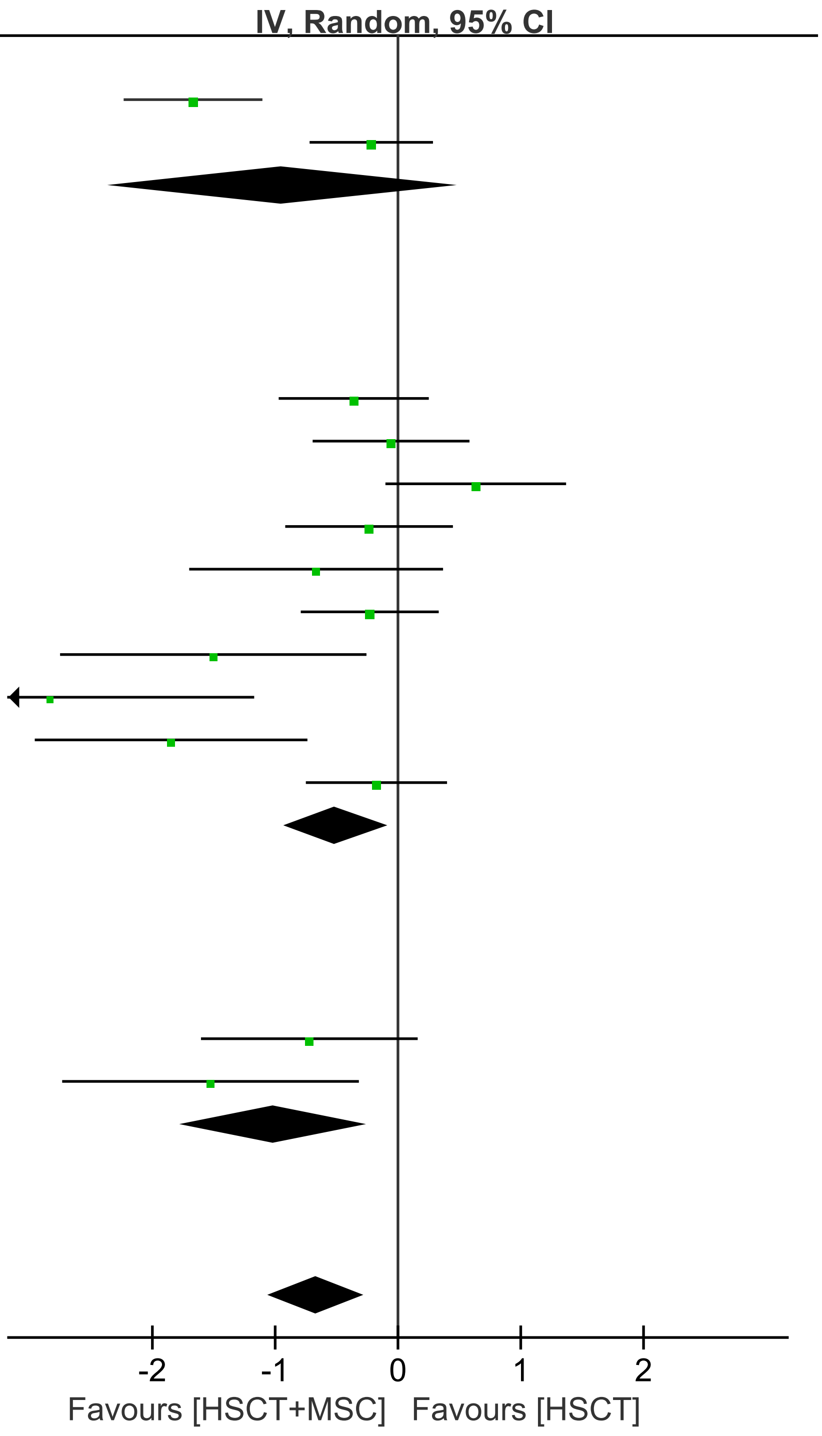

c

	HSCT+MSC			HSCT				Std. Mean Difference
Study or Subgroup	Mean	SD	Total	Mean	SD	Total	Weight	IV, Random, 95% CI
**2.3.1 ≤18 years old**								
Ball. 2007	11.25	2.79	14	20.56	28.87	47	8.4%	-0.36 [-0.96, 0.24]
Bernardo. 2011	38.45	27.11	13	39.77	21.74	39	8.2%	-0.06 [-0.68, 0.57]
Lee. 2013	51.58	13.84	7	62.87	17.47	9	6.1%	-0.67 [-1.69, 0.36]
Mareika. 2016	21.74	10.15	10	44.82	40.45	12	6.9%	-0.72 [-1.59, 0.15]
Wu. 2013a	31.73	5.49	5	75.52	17.29	9	3.7%	-2.83 [-4.48, -1.18]
Wu. 2013b	31.14	8.79	8	78.94	30.91	12	5.8%	-1.85 [-2.95, -0.75]
Xiang. 2017	15.42	2.59	32	16.02	2.86	32	8.9%	-0.22 [-0.71, 0.27]
**Subtotal (95% CI)**			**89**			**160**	**48.0%**	**-0.71 [-1.22, -0.19]**
Heterogeneity: Tau² = 0.28; Chi² = 17.17, df = 6 (P = 0.009); I² = 65%								
Test for overall effect: Z = 2.70 (P = 0.007)								
**2.3.2 >18 years old**								
Daganzo. 2009	52.05	21.18	9	36.25	25.14	46	7.7%	0.63 [-0.09, 1.36]
Hou. 2010	22.27	10.47	15	26.43	20.92	20	8.0%	-0.24 [-0.91, 0.44]
Liu. 2011	22.21	13.84	26	26.59	22.8	25	8.6%	-0.23 [-0.78, 0.32]
Wang. 2015	19.48	1.19	7	21.96	1.83	7	5.2%	-1.50 [-2.74, -0.27]
Xiao. 2013	8.71	6.02	7	19.5	7.15	8	5.3%	-1.53 [-2.72, -0.33]
Zhang. 2015	17.4	8.72	22	19.16	10.73	27	8.6%	-0.18 [-0.74, 0.39]
**Subtotal (95% CI)**			**86**			**133**	**43.4%**	**-0.35 [-0.86, 0.17]**
Heterogeneity: Tau² = 0.25; Chi² = 14.07, df = 5 (P = 0.02); I² = 64%								
Test for overall effect: Z = 1.31 (P = 0.19)								
**2.3.3 Not Reported**								
Ghavamzad. 2017	27.06	2.61	41	36.45	8.09	29	8.6%	-1.67 [-2.22, -1.11]
**Subtotal (95% CI)**			**41**			**29**	**8.6%**	**-1.67 [-2.22, -1.11]**
Heterogeneity: Not applicable								
Test for overall effect: Z = 5.90 (P < 0.00001)								
**Total (95% CI)**			**216**			**322**	**100.0%**	**-0.66 [-1.06, -0.27]**
Heterogeneity: Tau² = 0.40; Chi² = 51.82, df = 13 (P < 0.00001); I² = 75%								
Test for overall effect: Z = 3.28 (P = 0.001)								
Test for subgroup differences: Chi² = 12.35, df = 2 (P = 0.002), I² = 83.8%								

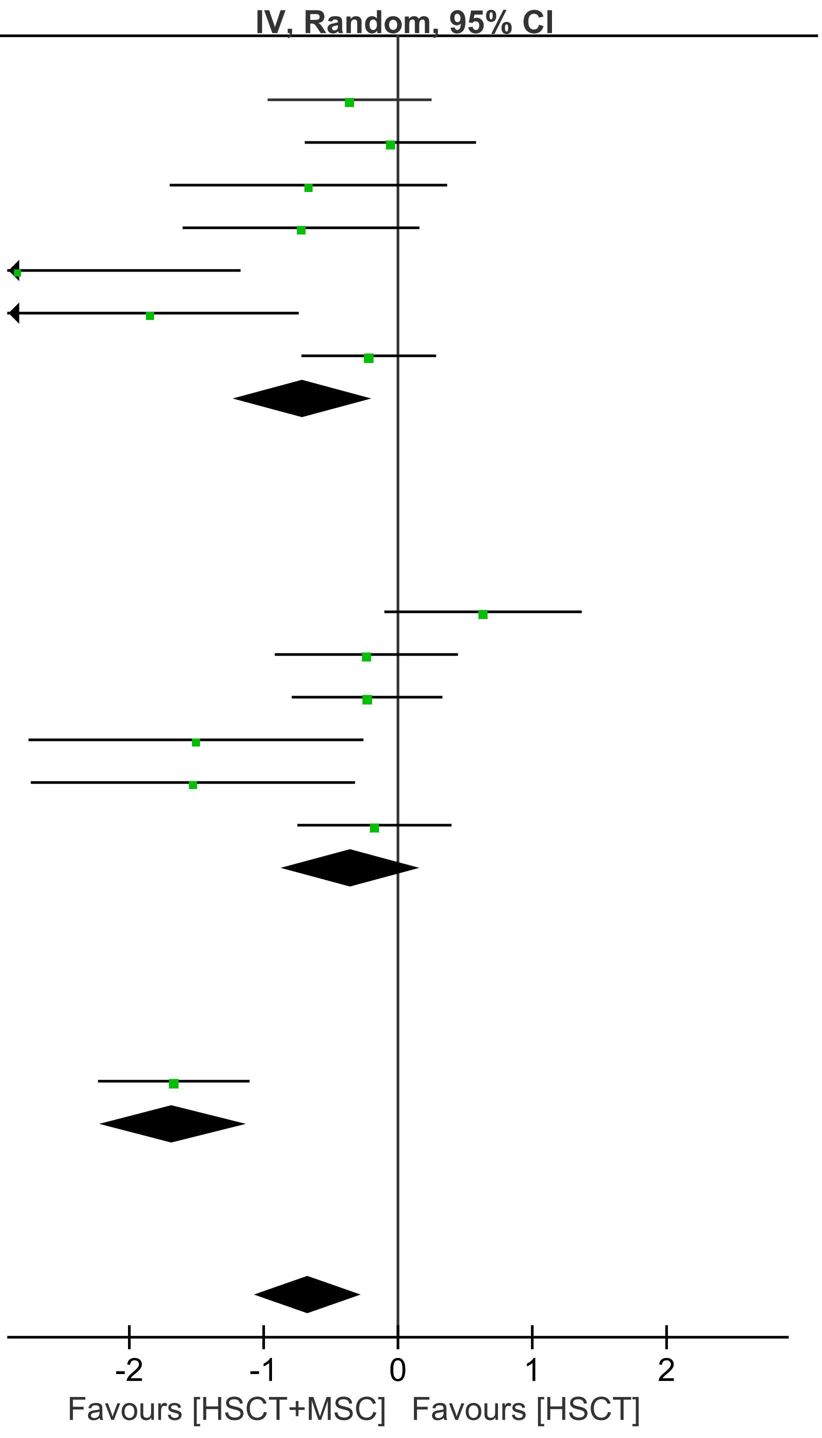

Supplement: Supplementary file 8 — Additional file 8: Fig. S6. Assessment of platelet engraftment in subgroup analysis according to (a) type of disease, (b) HLA matching and (c) average age. [file 13287_2021_2304_MOESM8_ESM.pdf]
